# Investigation of Substrate Specificity in 5‐Keto‐4‐Deoxy‐Glucarate Dehydratase and 2‐Keto‐3‐Deoxy‐L‐Arabinonate Dehydratase

**DOI:** 10.1002/cbic.70472

**Published:** 2026-07-24

**Authors:** Kathrin Hörnschemeyer, Esther Pruna, Sílvia Osuna, Mikael Boden, Volker Sieber

**Affiliations:** ^1^ Chemistry of Biogenic Resources Technical University of Munich Straubing Germany; ^2^ Institut de Química Computacional i Catàlisi University of Girona Girona Spain; ^3^ ICREA Barcelona Spain; ^4^ School of Chemistry and Molecular Biosciences University of Queensland St Lucia Queensland Australia

**Keywords:** bioinformatics, enzyme catalysis, protein engineering

## Abstract

5‐Keto‐4‐deoxy glucarate dehydratase and 2‐keto‐3‐deoxy‐L‐arabinonate dehydratase are members of the dihydrodipicolinate synthase superfamily of aldolase class I enzymes. The enzymes are part of the oxidative nonphosphorylated metabolic pathways for the production of 2‐ketoglutarate from glucuronic acid and arabinose, respectively. They carry out a C4 dehydration on their substrates, 5‐keto‐4‐deoxy glucarate and 2‐keto‐3‐deoxy‐L‐arabinonate, and produce the same product, ketoglutarate semialdehyde. While the enzymes are similar in structure and reaction mechanism, they exhibit a high substrate specificity and have only been reported to be active on their native substrate. In order to investigate the high substrate specificity, we aimed to generate promiscuous variants of both enzymes that can accept multiple substrates and investigate the evolutionary development of the substrate specificity in the superfamily. Ancestral sequence reconstruction and GFN2‐xTB calculations were used to develop candidates for further analysis of substrate scope. Both approaches yielded multiple promiscuous dehydratases with activity on both substrates.

## Graphical abstract

5‐Keto‐4‐deoxy glucarate dehydratase and 2‐keto‐3‐deoxy‐L‐arabinonate dehydratase share many similarities like structure, reaction mechanism, and their generated product, but exhibit very high substrate specificity. Here, we explore the evolutionary development of this specificity in the context of the enzyme superfamily and attempt to find promiscuous dehydratases via ancestral sequence reconstruction and enzyme engineering.

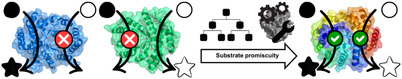

## 1 Introduction

The dihydrodipicolinate synthase (DHDPS) superfamily, also known as the N‐acetylneuraminate lyase (NAL) superfamily, is a widely available enzyme family. It is part of the aldolase class I enzymes, and the members contain the characteristic TIM (β/α)_8_‐barrel structure and a catalytic lysine, which forms a Schiff base with the substrate [1, 2]. Well‐known members of the superfamily are DHDPS, NAL, 2‐keto‐3‐deoxy gluconate aldolase (KdgA) or trans‐o‐hydroxybenzylidenepyruvate hydratase‐aldolase (CHBPH), which all catalyze the C—C bond formation or cleavage of a pyruvate and a variable acceptor substrate [1, 3].

**SCHEME 1:**
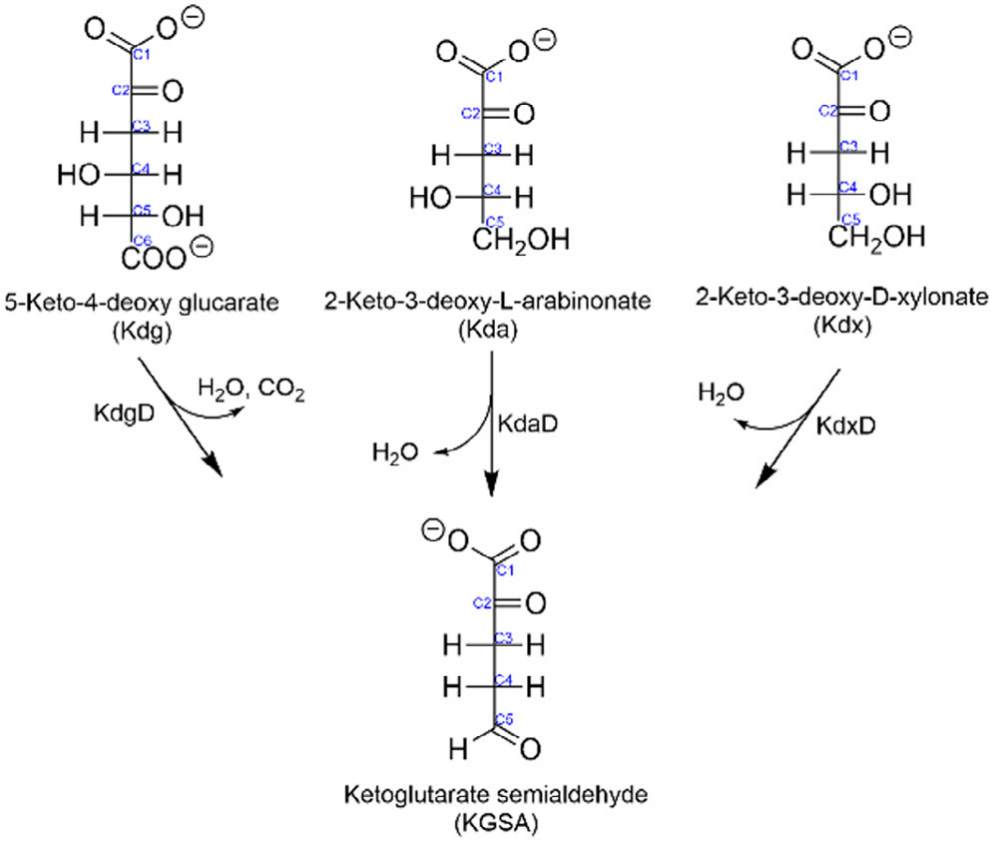
Reaction overview of 5‐keto‐4‐deoxy glucarate dehydratase (KdgD, EC 4.2.1.41), 2‐keto‐3‐deoxy‐L‐arabinonate dehydratase (KdaD; EC 4.2.1.43), and 2‐keto‐3‐deoxy‐D‐xylonate dehydratase (KdxD; EC 4.2.1.141). All three enzymes perform a dehydration on their substrate to produce 2‐keto glutarate semialdehyde. KdgD also performs a decarboxylation [5, 6]. The carbon chain of the chemical structures annotated in blue.

There are, however, two subgroups annotated as members of this superfamily that do not perform an aldol condensation, but instead a dehydration reaction. 5‐keto‐4‐deoxy glucarate dehydratases (KdgD; EC 4.2.1.41) and 2‐keto‐3‐deoxy‐L‐arabinonate dehydratase (KdaD; EC 4.2.1.43) are part of the nonphosphorylated pathways for the production of 2‐keto glutarate (2 KG). KdgD takes part in the conversion of hexuronic acids like glucuronic acid or galacturonic acid, where it catalyzes the conversion of 5‐keto‐4‐deoxy glucarate (Kdg) to ketoglutarate semialdehyde (KGSA), the direct precursor of 2 KG (Scheme 1). In addition to the dehydration, a decarboxylation occurs, going from a C6 sugar acid to a C5 molecule [4, 5]. KdaD, on the contrary, is a part of the Weimberg pathway starting from arabinose. It catalyzes the reaction analogous to KdgD, producing KGSA from 2‐keto‐3‐deoxy‐L‐arabinonate (Kda), but without the additional decarboxylation [6]. Similar to the pathway from arabinose to 2 KG is the pathway from xylose to 2 KG. However, the 2‐keto‐3‐deoxy‐D‐xylonate dehydratase (KdxD; EC 4.2.1.141), which performs the analogous reaction to KdgD and KdaD in this pathway, is not part of the DHDPS superfamily, but instead is annotated as a member of the fumarylacetoacetate hydrolase family [7].

While the reactions of KdgD and KdaD are different from the rest of the superfamily, they are very similar to each other. Both catalytic mechansims follow the same steps and only differ by a decarboxylation in the KdgD reaction that is replaced with a proton abstraction in KdaD. Despite all similarities, these enzymes are highly selective for their native substrate. This stems the question of how the superfamily evolved to have two subgroups of enzymes that perform an almost identical reaction on highly similar substrates but show no activity on the substrate of the other enzyme. Since both enzyme groups are part of the same superfamily, it can be assumed that they developed from a shared ancestor. The question we asked is whether the ancestor has a promiscuous function toward both substrates or if one of the activities developed from the other one. A last possibility would be that both activities developed separately, which would be indicated by a shared ancestors with no activity toward either but a third substrate.

Here, we wanted to investigate the evolutionary development of the DHDPS superfamily, especially of the KdgD and KdaD subgroups, as well as find a promiscuous dehydratase with a broader substrate scope to perform the reaction of Kdg, Kda, and maybe even Kdx. To achieve this, two approaches were taken. The superfamily and its evolutionary development were analyzed using ancestral sequence reconstruction [8, 9] to find ancestors of KdgD and KdaD and analyze them for their substrate specificity. Additionally, computational methods were employed to determine active site mutations in KdgD from *Acinetobacter baylyi* (*Ab*KdgD; Uniprot Q6FFQ1) and KdaD from *Cupriavidus necator* (*Cn*KdaD; Uniprot Q0K4R0) in order to generate a promiscuous variant or to interconvert the activities.

## 2 Results and Discussion

### 2.1 Ancestral Sequence Reconstruction

**FIGURE 1:**
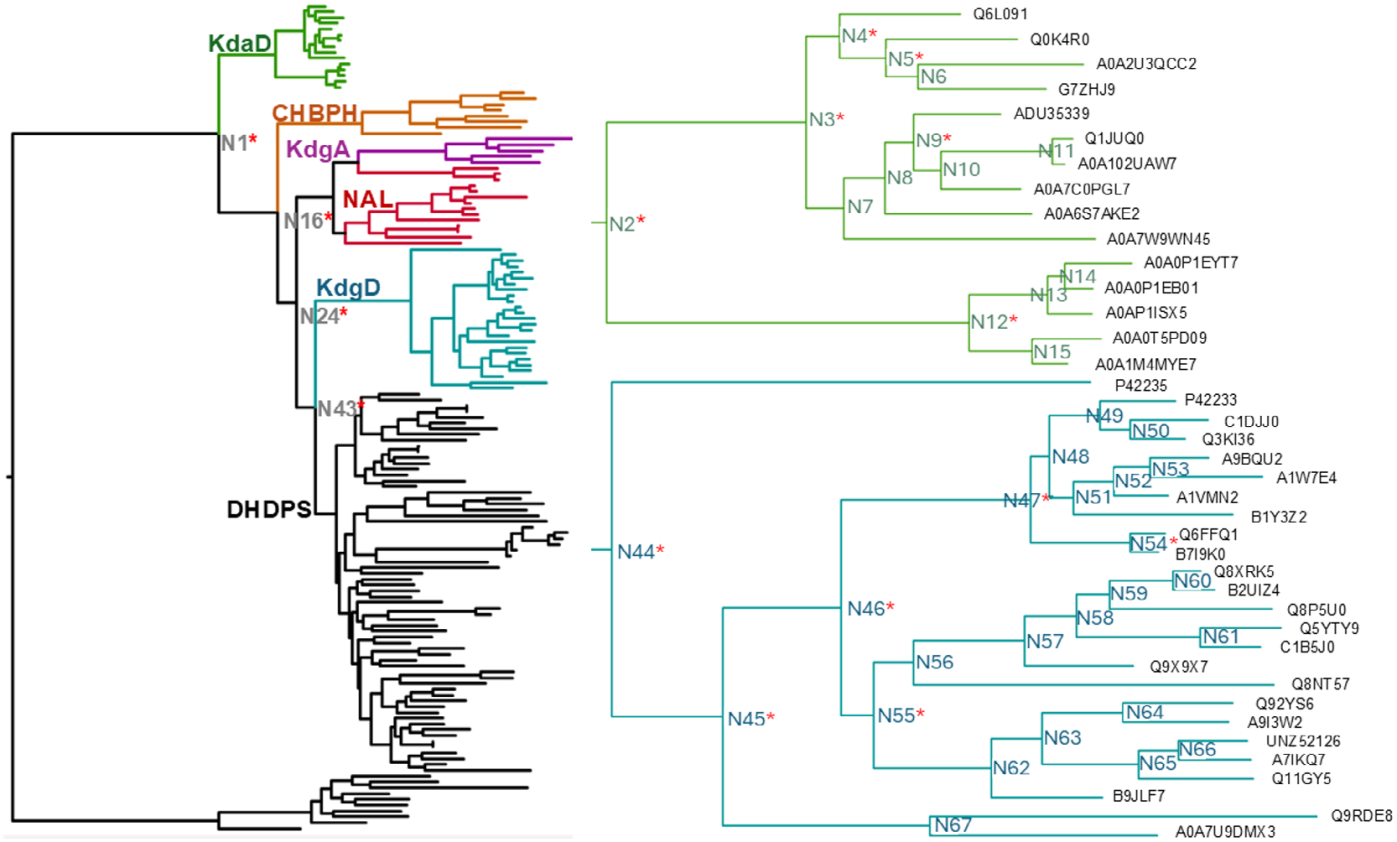
Phylogenetic tree was generated with IQtree from a dataset containing 132 sequences of the DHDPS/NAL superfamily aligned with Promals3D. Subfamilies are highlighted in different colors (KdaD—green, CHBPH—orange, KdgA—violet, NAL—red, KdgD—blue, DHDPS—black). Ten Sequences of the DERA enzyme family were added as the outgroup for the rooting process. On the right is a zoom‐in on the clades of the KdaD (top) and KdgD (bottom) clades, with uniport IDs for the wildtype sequences shown. Nodes are labeled by number, and nodes marked with a red star were chosen to be reconstructed and characterized.

For the generation of the phylogenetic tree, a dataset representing the entire DHDPS/NAL superfamily was created. The dataset contained 132 sequences from all parts of the superfamily in an attempt to generate an as accurate as possible overview of the evolutionary development of KdgD and KdaD in the context of their superfamily. The number of sequences from each subfamily was chosen to roughly represent the overall ratios of the subfamilies based on the Uniprot database, though the percentage of the DHDPS family had to be scaled down to not completely dominate the remaining sequences. Additionally, 10 sequences of the deoxyribose‐phosphate aldolase (DERA) family, a different enzyme family of the aldolase class I, were used as an outgroup for the tree rooting. The sequence similarity within the DHDPS/NAL superfamily was rather low; however, the structure was highly conserved. Because of that, the structural alignment tool Promals3D [10] was used to generate a multiple sequence alignment. The alignment was used to generate the phylogenetic tree with maximum likelihood calculation using the IQ tree server [11]. In the phylogenetic tree (Figure 1), the KdaD clade was observed to separate first from the remainder of the superfamily, which was expected as KdaD differed the most in the conversed residues of the superfamily; for example, KdaD contained a catalytic glutamate (E174 for *Cn*KdaD) at the position where the rest of the superfamily had a conserved tyrosine [2, 4, 6]. The KdgD clade was the second youngest clade, after the DHDPS clade, which seems plausible as they share some conserved catalytic residues like the above‐mentioned catalytic tyrosine (Y141 for *Ab*KdgD). High bootstraps were observed for most of the branches, so the phylogeny was used for the reconstruction of the sequences using Graphical Representation of Ancestral Sequence Predictions (GRASP) [12].

**FIGURE 2:**
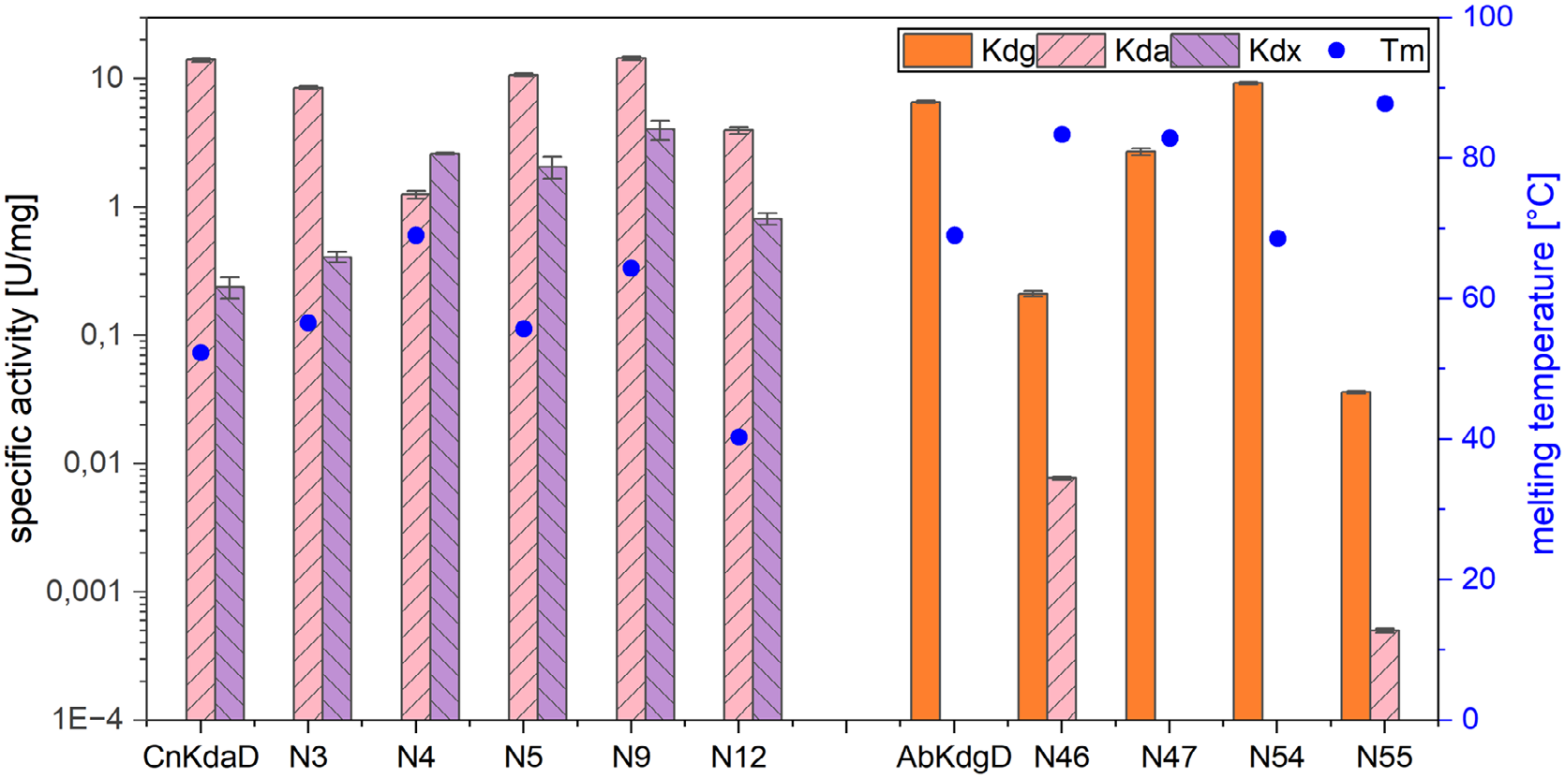
Activities of wildtype and active ancestral KdaDs and KdgDs. Specific activity in U/mg for the specified substrate was measured in 50 mM HEPES buffer (pH 7.5) with 5 mM MgCl_2_, 2.5 mM NAD, 5 mM of substrate, 170 nM KGSA dehydrogenase of *Pseudomonas putida* (Uniprot A0A0P7CZ02) and 0.01–1 mg/mL of the target enzyme. Error bars represent the standard deviation between triplicates. Melting points were determined via Thermofluor assay and are shown in °C for each enzyme.

To investigate the evolutionary development of the KdgD and KdaD subfamily, the sequences of 16 nodes along the path from the last common ancestor (Ancestor N1) to the extant KdgDs and KdaDs were reconstructed (see Figure 1, red stars). These ancestors were expressed in *E. coli* BL21 in autoinduction medium with 5 mM betaine and 1 M sorbitol added, following the expression conditions of *Ab*KdgD [13]. Of the 16 enzymes, 7 exhibited insoluble expression (Ancestors N1, N2, N16, N24, N43, N44, and N45) and were thus tagged with the maltose binding protein (MBP) to increase the solubility. Expression of the now tagged enzymes did indeed show soluble expression, and all ancestral enzymes were purified via affinity chromatography and size exclusion chromatography. The purified enzymes were investigated for their activities and stability (Figure 2). MBP‐tagged enzymes were evaluated for their activity before and after cleavage of the tag.

It could be seen that the ancestors of the KdaD clade (N3‐N12) showed activity on Kda and Kdx, like the *Cn*KdaD wildtype. Low promiscuity of KdaDs on Kdx was not surprising, as the two substrates are stereoisomers of each other. The KdgD ancestors N47 and N54 only showed activity on Kdg, while ancestors N46 and N55 exhibited low secondary Kda activity in addition to their activity on Kdg. While the Kda activity of N46 and N55 is 4–5 orders of magnitude lower than that of the *Cn*KdaD wildtype, the assay was performed with controls for every assay component to ensure the measurement of real activities. The ancestors with solubility problems did not show any activity, regardless of whether the MBP‐tag was present or cleaved off, and are therefore not shown in Figure 2.

The melting points of these enzymes were determined via a Thermofluor assay (Figure S35). For the active ancestors it was seen that generally the older, less active ancestors exhibited higher melting points that slowly decreased over time as they got closer to the extant wildtypes. No melting points were measured for the inactive ancestors after cleavage of the MBP‐tag. This suggests improper folding of the enzyme, likely due to a wrong amino acid sequence as the result of the sequence reconstruction. Since the bootstrapping of the phylogenetic tree revealed high values for most of the branches, the issue might lie with the dataset itself instead of the tree generation. The dataset with 142 sequences is on the smaller side, which could play a role in receiving inaccurate sequences from the reconstruction. The dataset was chosen to be kept small in order to keep the calculation time to a reasonable amount, as previous studies with datasets of a similar size have been reported successful [14, 15, 16, 17]. The reconstruction could have also been hindered by the inclusion of the different subfamilies of the DHDPS/NAL superfamily, which leads to KdgD and KdaD only making up a small part of the full dataset. This was originally done to see the development of the dehydratases within the superfamily and give evolutionary context. However, since the superfamily exhibits low sequence identity, the presence of these subfamilies might have introduced too much noise into the dataset.

Overall, the activity of the ancestors was observed to decrease with the age of the ancestors, which follows the idea that the extant enzymes today are highly specialized but evolved over time from more generalistic ancestors, which in turn have lower activities for more possible reactions [18]. The promiscuity of ancestors N46 and N55 is in alignment with this theory, representing the last ancestors that exhibit the shared activity. However, with the inactivity of the older ancestors, it cannot be determined if the activities developed from a promiscuous shared ancestor or if the KdgD activity developed over time from KdaD activity.

**FIGURE 3:**
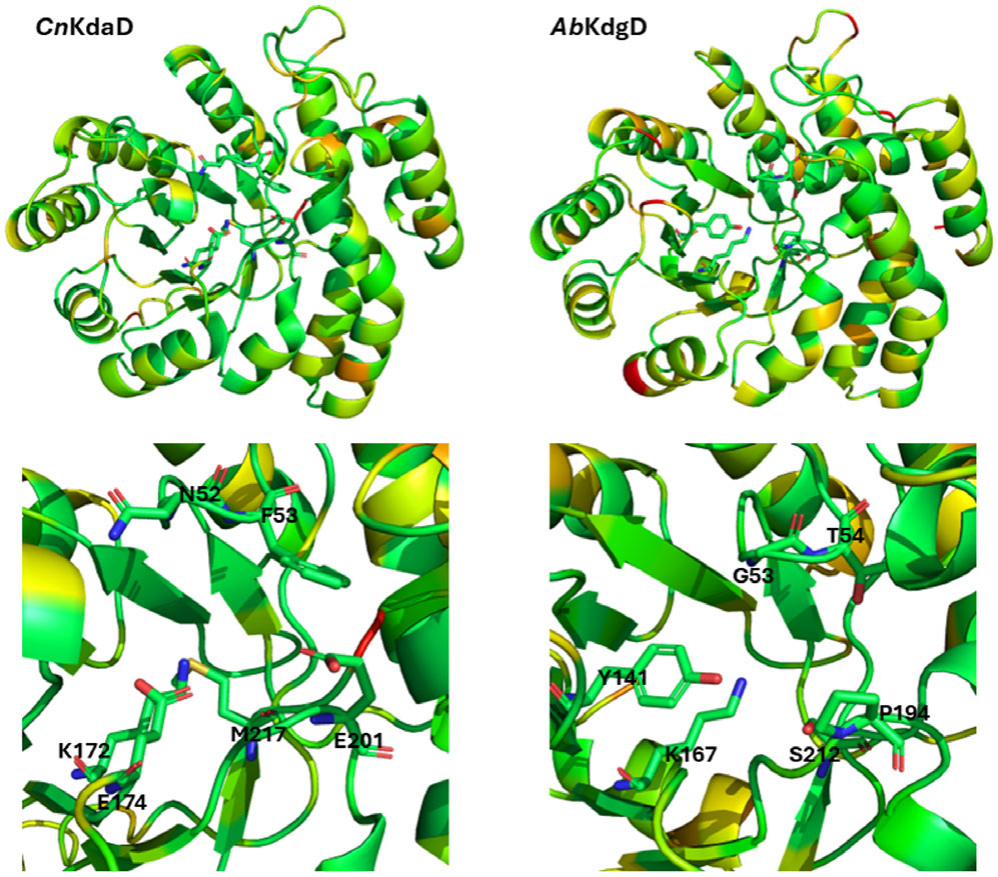
Structure of *Cn*KdaD (left) and *Ab*KdgD (right) with a zoom‐in of their respective active site below. Structures are colored by alignment score of a multiple sequence alignment of the respective wildtype and all ancestors within their clade. Higher conversion is shown by a green color, the lower the conversion of one residue the redder the coloring gets. Active site residues relevant to the catalytic mechanism are shown as sticks and annotated.

Still, the promiscuous activity of N46 and N55 for Kda was unexpected, as the active site of all ancestors within the KdgD clade is very conserved. In Figure 3, the structures of *Cn*KdaD and *Ab*KdgD are shown colored by how conserved each residue is in an alignment of the wildtype with all ancestors of their respective clades. It can be seen that the active sites at the core of the structures are colored green, representing high conversion. Mutations between the variants, represented by a more yellow–red color, mostly occur in the outer shells of the enzymes. This highlights that the active sites and therefore, catalytic mechanisms of the two enzymes likely developed very early on. The promiscuous function of N46 and N55 is possibly rooted in a general higher flexibility of the enzyme, possibly due to the outer shell mutations. This was previously described as the reason for promiscuity in ancestral enzymes [18]. Unfortunately, the ancestral analysis did not yield more insights on which residues in the active site are responsible for the substrate selectivity in the wildtype enzymes.

### 2.2 Active Site Engineering

Besides using the natural diversity and evolution to identify promiscuous variants, we were curious whether the promiscuity could be engineered. *Ab*KdgD and *Cn*KdaD were chosen as wildtypes for the active site engineering, as they have been reported previously to have high activity for their native substrate [13] and showed to be employable in cascade reactions [19, 20]. To determine possible active site mutations, first, the binding of the native and nonnative substrates in the enzymes was analyzed by constructing a cluster model of the active site pocket and performing geometry optimizations at the GFN2‐xTB [21] level of calculation (see Methods for a full description). The models of the two active sites were prepared with the substrates bound to the catalytic lysine via Schiff base formation. The optimized geometries were used to analyze how the native substrate is bound to the active site and what interactions are missing or hindering the binding of the nonnative substrate.

**FIGURE 4:**
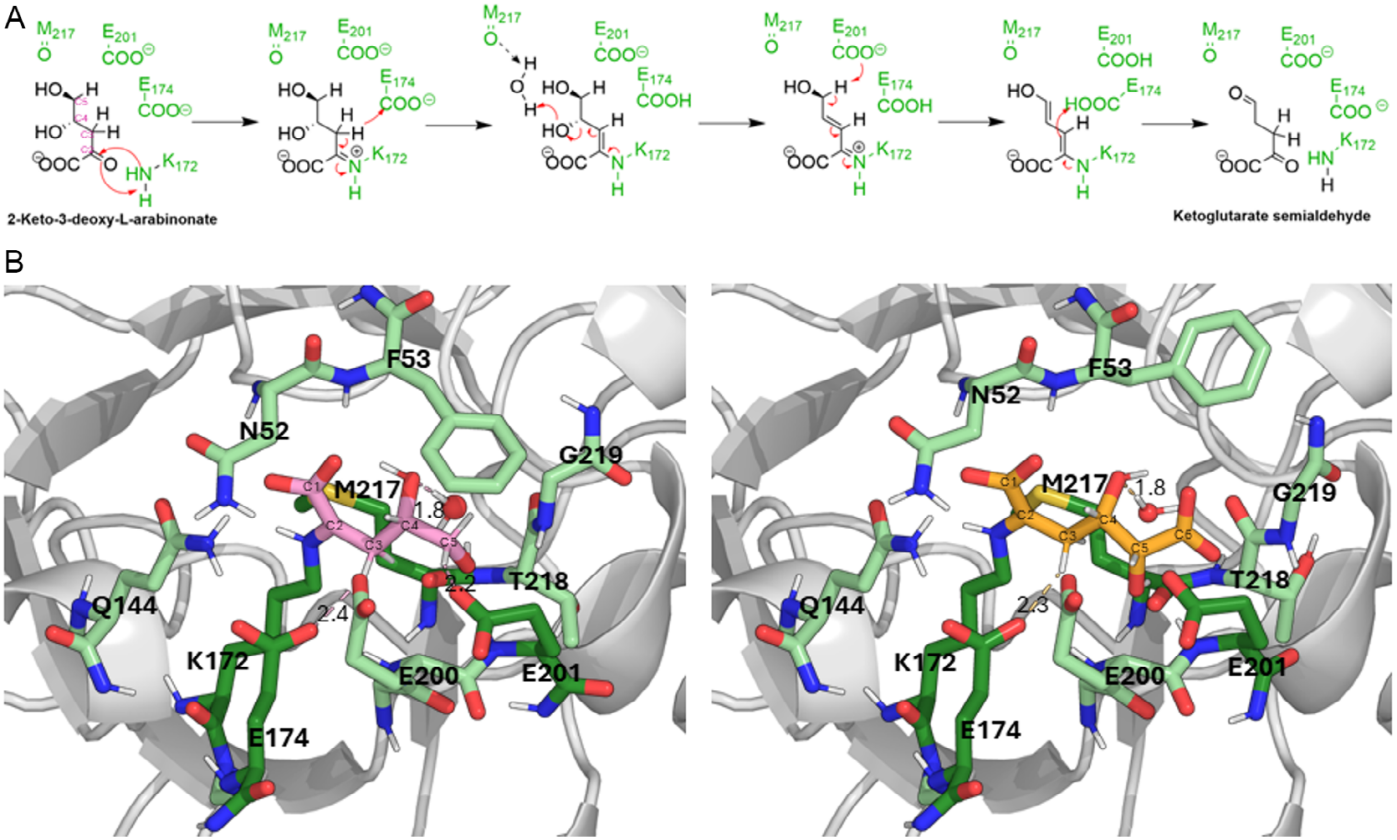
(A) Overview of the reaction mechanism in *Cn*KdaD. (B) Cluster model analysis of the binding of CnKdaD with its native substrate Kda (pink, left) and Kdg (orange, right). Active site residues are shown as sticks, catalytic residues are highlighted in a darker color. Catalytically relevant interactions are shown as dashes with their distances in angstroms.

In Figure 4B, the model of *Cn*KdaD with Kda showed a coordination via hydrogen bridges of the C1 carboxylic acid group of Kda with the backbone amides of N52 and F53 as well as with the sidechain of Q144. As per the reaction mechanism (Figure 4A), E174 formed a hydrogen bridge with Kda‐C3 and E201 with Kda‐C5, so both glutamates can carry out a deprotonation of the respective carbon. The catalytic water was coordinated between the Kda‐C4 hydroxy group and the sidechain carbonyl of M217. The binding of Kda in the model was therefore in accords with the proposed mechanism. The model of *Cn*KdaD with Kdg showed a similar coordination in the first five carbon atoms, though the hydrogen bridge between E201 and Kdg‐C5 is less important as a C5 deprotonation was not necessary for the Kdg dehydration. The main differences were that the catalytic water was no longer coordinated by the sidechain carbonyl of M217, but instead it formed a hydrogen bridge to the Kdg‐C6 carboxylic acid group, and the sidechain of F53 seemed to be pushed away from the active site due to larger size of Kdg compared to Kda. Additionally, it needs to be stated that the active site contained three glutamate residues near the entrance, which could lead to electrostatic repulsion between the active site and Kdg.

**FIGURE 5:**
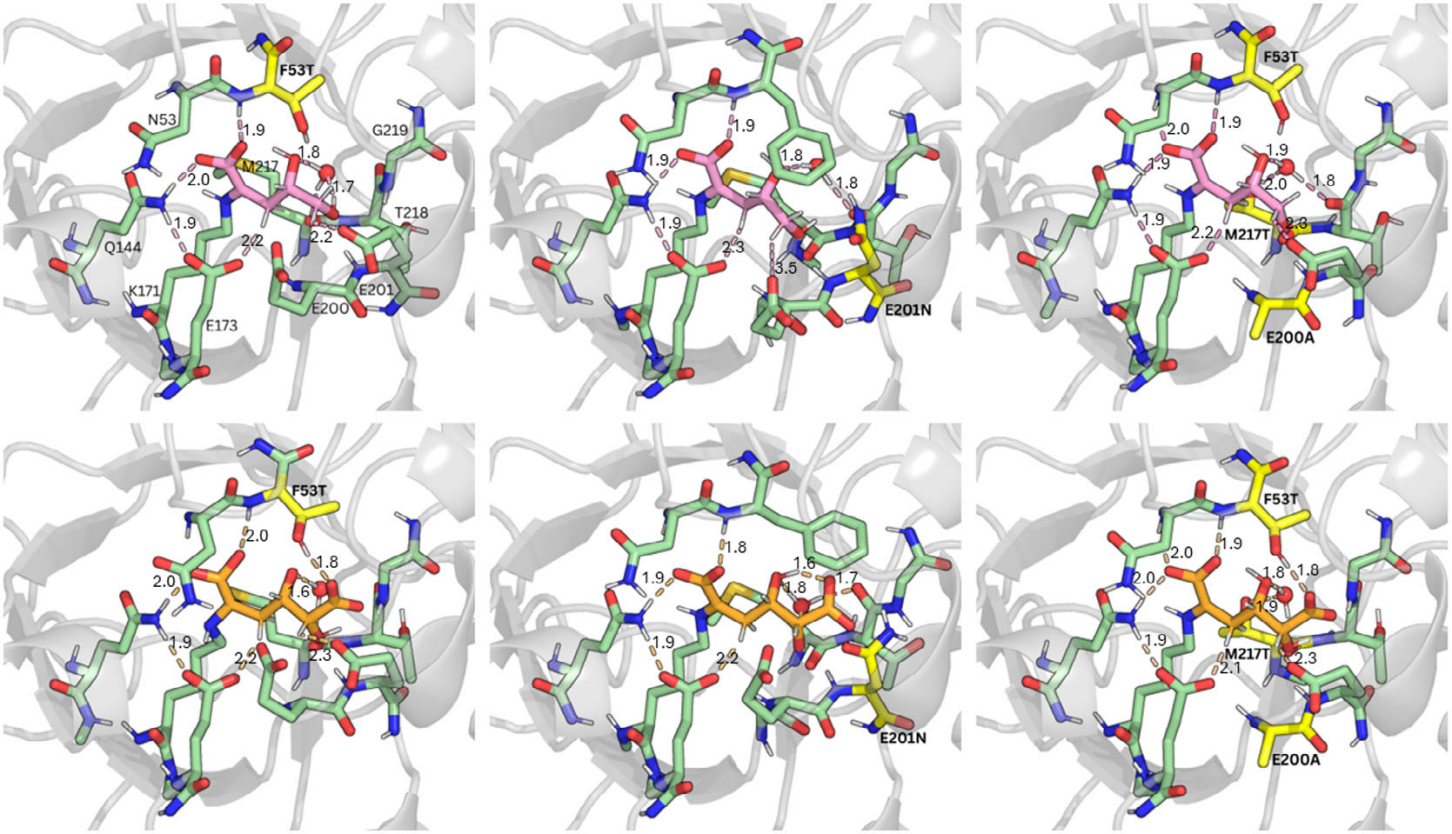
Display of how mutations in the *Cn*KdaD active site change the binding of Kda (pink, top) and Kdg (orange, bottom). The introduced mutations highlighted in yellow are from left to right: F53T; E201N; F53T E200A M217T. Interactions between the residues and the substrate are shown as dashes with their distance in angstroms.

**FIGURE 6:**
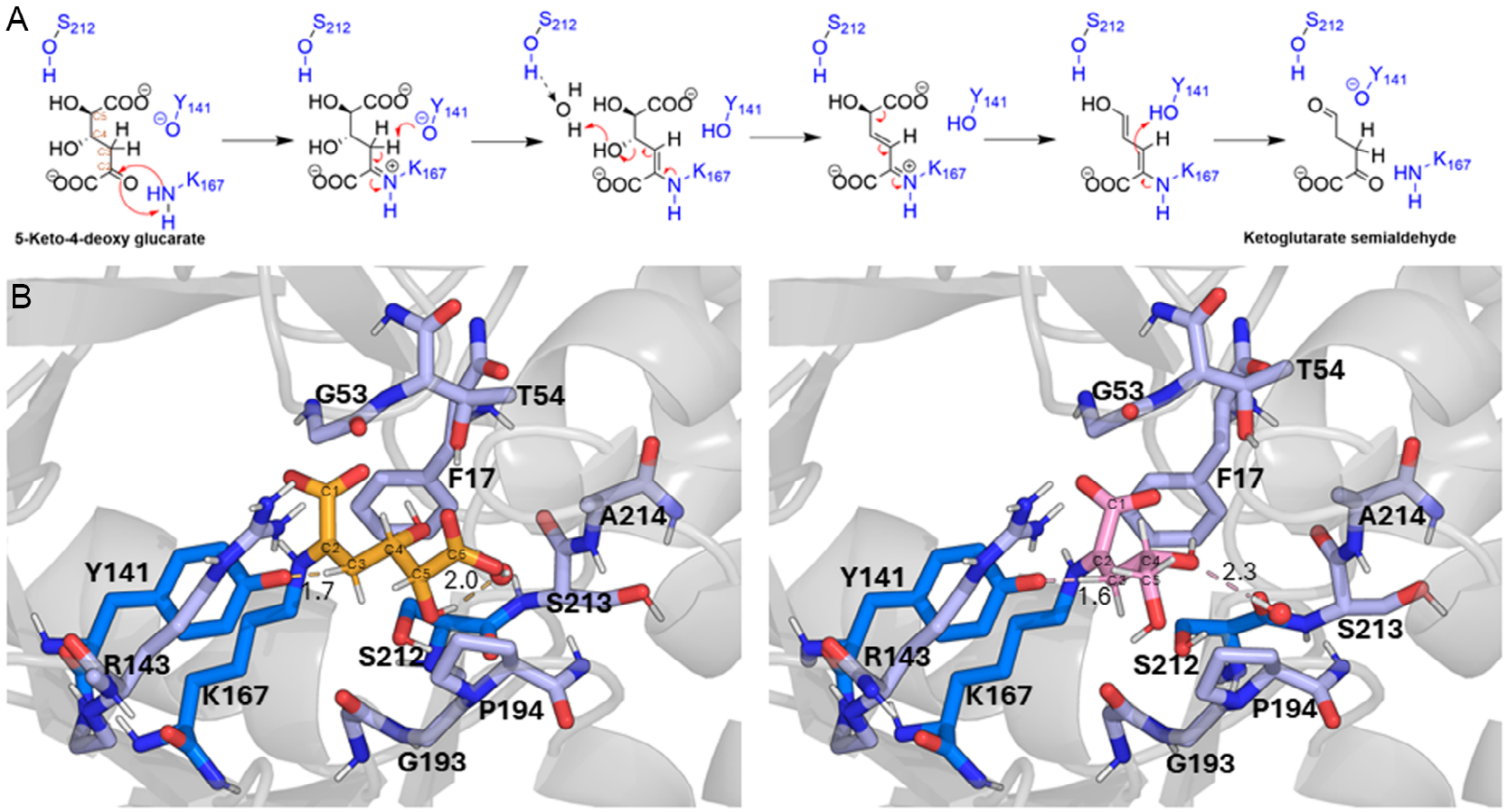
(A) Overview of the reaction mechanism in *Ab*KdgD. (B) Cluster model analysis of the binding of *Ab*KdgD with its native substrate Kdg (orange, left) and Kda (pink, right). Active site residues are shown as sticks, catalytic residues are highlighted in a darker color. Catalytically relevant interactions are shown as dashes with their distances in angstroms.

Engineering *Cn*KdaD for Kdg activity started by enlarging the active site through a F53T mutation (Figure 5), This mutation additionally facilitated the coordination of the Kdg‐C6 carboxylic group as seen in *Ab*KdgD (Figure 6B). To decrease the potential electrostatic repulsion between Kdg and the *Cn*KdaD active site, the two glutamates E200 and E201 were targeted with mutations to alanine or asparagine. The mutations of E201 were at a higher risk of eliminating the native activity as it is the catalytic residue, however, this residue is closer to the Kdg‐C6 group and would have a larger impact on the repulsion. Lastly, the peptide backbone of M217 is involved in the coordination of the catalytic water, this function is carried out by a serine in *Ab*KdgD. A mutation of M217 to serine or threonine was shown to form a hydrogen bridge to the water molecule, therefore likely improving the coordination. Combinations of the different mutations were tested, and the most promising variants were characterized in vitro (Figure 8).

The model of *Ab*KdgD with Kdg in Figure 6B showed a hydrogen bond between the C1 carboxylic group of the substrate and the backbone amides of G53 and T54. Mechanistically, Y141 deprotonates Kdg at the C3 position, the model showed this with a hydrogen bond. Additionally, the hydroxyl groups of C4 and C5 interacted with a catalytic water, and the C6 carboxylic group interacted with the sidechain of T54.The binding of Kda by *Ab*KdgD showed similarities in the first four carbon atoms of the substrate, however coming to Kda‐C5 it is obvious that, in the KdgD active site, there is no equivalent residue to perform the final deprotonation of Kda‐C5. Furthermore, due to the smaller size of Kda compared to Kdg the substrate binding was looser, which was seen by the greater distance between the catalytic water and the substrate (distance of 2.0 Å compared to 2.3 Å, Figure 6B).

**FIGURE 7:**
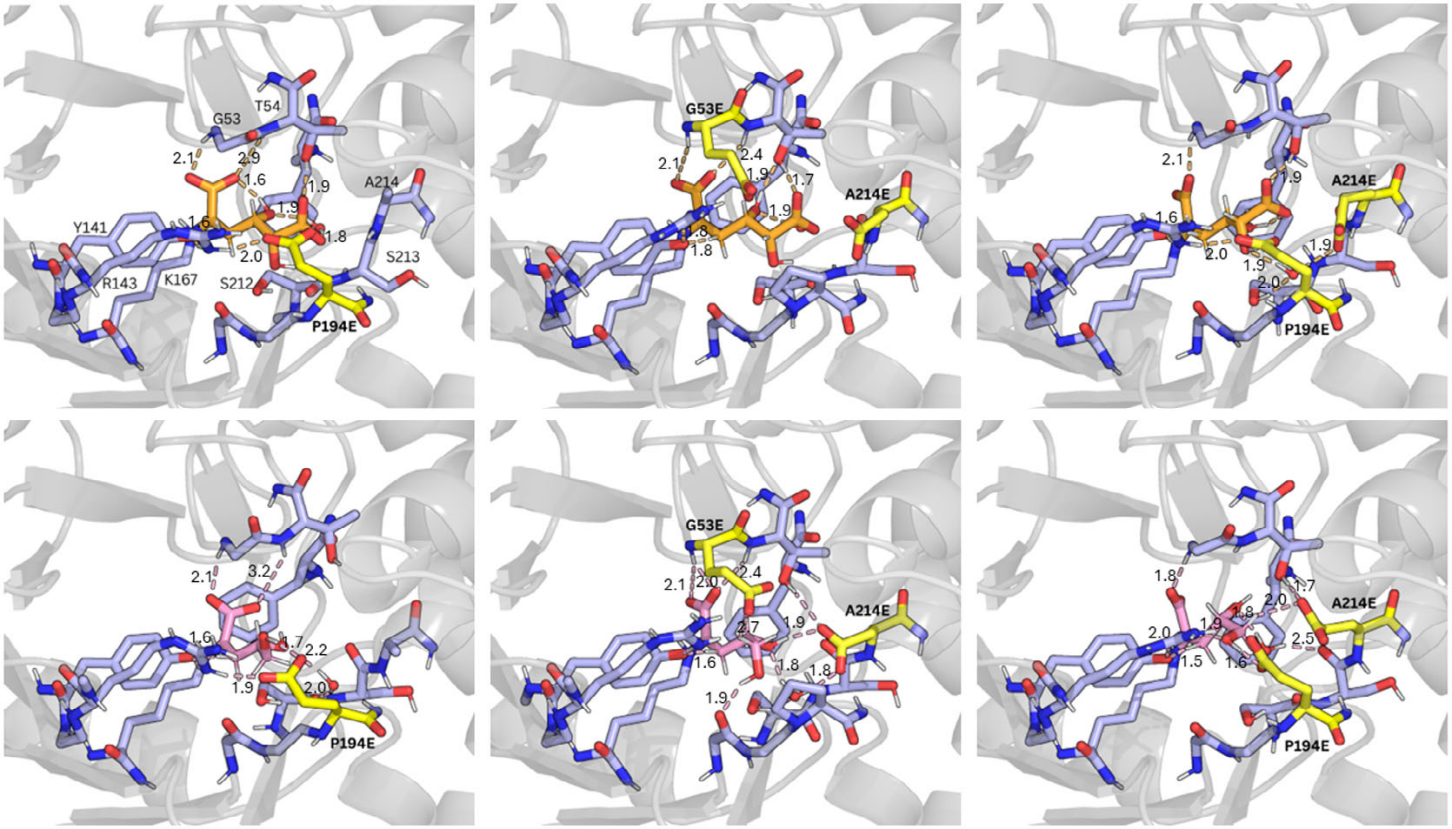
Display of how mutations in the *Ab*KdgD active site change the binding of Kdg (orange, top) and Kda (pink, bottom). The introduced mutations highlighted in yellow are from left to right: P194E; G53E A214D; P194E A214E. Interactions between the residues and the substrate are shown as dashes with their distance in angstroms.

**FIGURE 8:**
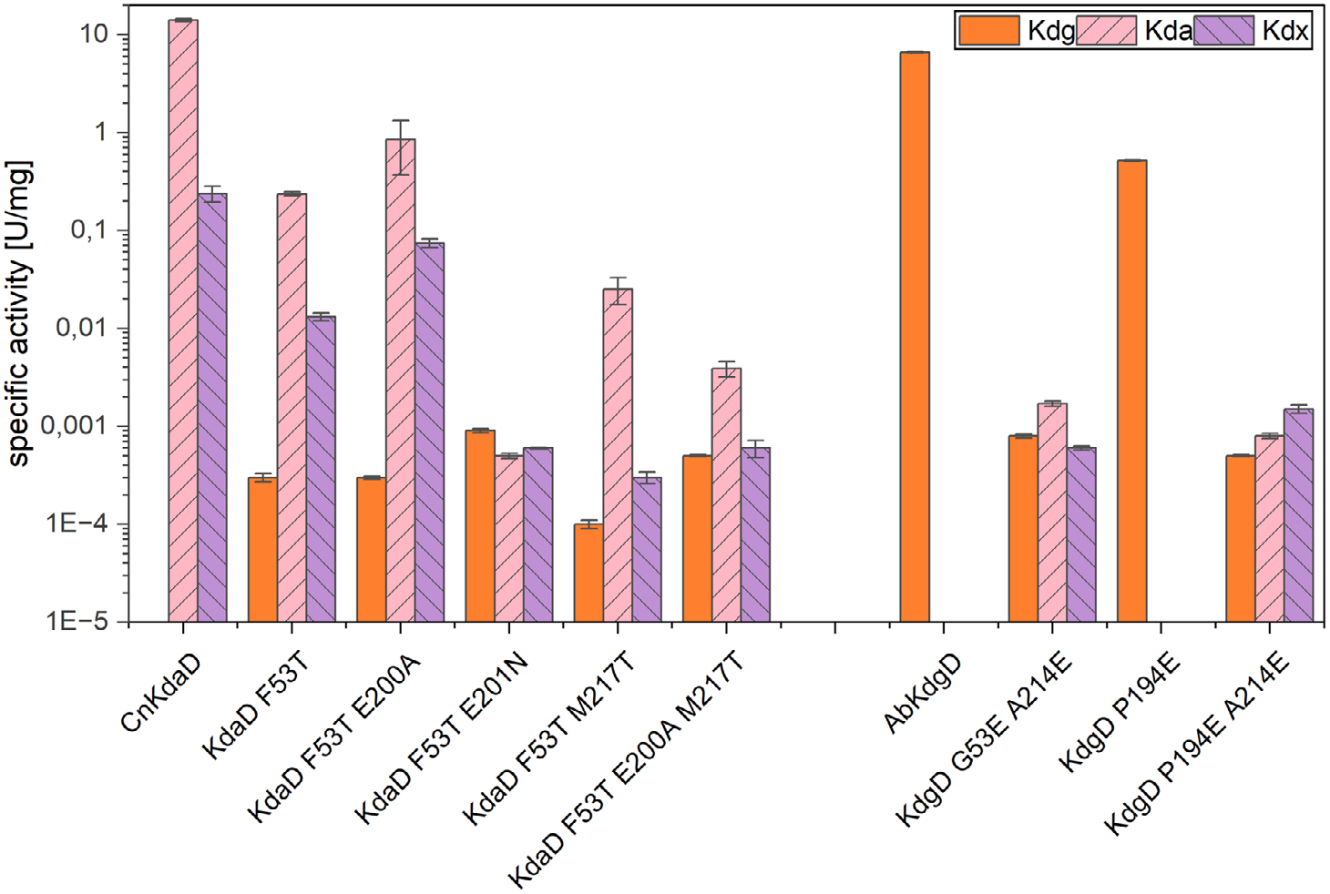
Specific activity of the wildtypes, *Cn*KdaD and *Ab*KdgD, and the generated variants to showcase the promiscuous activity. Specific activity in U/mg for the specified substrate was measured in 50 mM HEPES buffer (pH 7.5) with 5 mM MgCl_2_, 2.5 mM NAD, 5 mM of substrate, 170 nM KGSA dehydrogenase of *Pseudomonas putida* (Uniprot A0A0P7CZ02) and 0.01–1 mg/mL of the target enzyme. Error bars represent the standard deviation between triplicates.

To generate activity for Kda in *Ab*KdgD, acidic residues were introduced to facilitate the deprotonation of Kda at the C5 position and to improve the coordination of the water molecule (Figure 7). Additionally, the added residues, being either glutamate or aspartate, were rather large and therefore ensured a tighter fit of the substrate in the active site. Positions that were mutated are G53, P194, and A214 as these are noncatalytic and do not carry out a function for the substrate binding. A214 mutation to glutamate or aspartate also imitated the C6 carboxylic group of Kdg since the sidechain was in a similar place as the C6 group of the native substrate, which provided a coordination to the catalytic water that resembled that of wildtype KdgD with Kdg. The variant *Ab*KdgD P194E was chosen for in vitro testing as the introduced glutamate is homolog to the catalytic glutamate of *Cn*KdaD (E201), so the effect of this mutation was intriguing. Different combinations of the mutations were tested and *Ab*KdgD G53E A214E and *Ab*KdgD P194E A214E were chosen for in vitro characterization as they were deemed most likely to show activity on both substrates (Figure 8).

The chosen mutans were expressed in *E. coli* BL21 in autoinduction medium containing 5 mM betaine and 1 M sorbitol. After purification via affinity chromatography and size exclusion chromatography, the mutants were analyzed in vitro (Figure 8). Analogous to the wildtype and the ancestors, these variants were investigated for their activity toward Kdg, Kda and Kdx. The *Cn*KdaD variants, *Ab*KdgD G53E A214E, and *Ab*KdgD P194E A214E exhibited promiscuity for all three substrates. Overall, the two KdgD variants and *Cn*KdaD F53T E201N showed a more balanced promiscuity between all three substrates, while the KdaD variants seemed to favor one of the substrates. Interestingly, the two KdgD variants showed a similar activity toward their native substrate, Kdg, but *Ab*KdgD G53E A214E showed most of its activity toward Kda, while *Ab*KdgD P194E A214E favored Kdx. This is because the G53E and P194E mutations are on opposite sides of the active site, therefore working better for one of the stereoisomers.

The promiscuous activity was achieved at the cost of a highly decreased catalytic efficiency as seen in Figure 8. The introduction of acidic residues into the KdgD active site increased the pK_a_ of either the catalytic tyrosine Y141 or the catalytic lysin K167 according to pK_a_ predictions (Table S9) made using PropKa in the APBS biomolecular solvation software suite with the amber force field [22]. Y141 and K167 both need to be deprotonated for the catalytic mechanism, which is less likely at a higher pK_a_. In addition to the changes in pK_a_, the active site had overall become more negatively charged due to the mutations. This means electrostatic repulsion between the substrate and the active site increases, reducing the substrate binding and thus the activity. Enlargement of the KdaD active site and reduction of the negative charge made the *Cn*KdaD variants active on Kdg. The mutation of F53 to a threonine allowed for the larger substrate, Kdg, to fit better into the active site, but at the same time opened the active site up and potentially made the proper coordination of the substrate difficult to achieve. Mutating the two glutamates E200 and E201 reduced the negative charge of the active site but also increased the flexibility of the not‐mutated glutamate, which likely had a negative effect on the activity. It was unexpected that *Cn*KdaD F53T E201N retained activity for Kda, since the E201N mutation targeted the catalytic glutamate, but it seemed that E200 took over the role of the catalytic residue to retain a low level of native activity. However, E200 in the wildtype was likely needed to regenerate the initial state of the active site by causing deprotonation of E201 after the reaction. Mutating one of the glutamates removed this function, which would explain the low activities of the variants.

## 3 Conclusion

Both strategies, the ancestral sequence reconstruction and the active site engineering to generate a promiscuous dehydratase for the conversion of Kdg, Kda, and Kdx yielded variants with measurable activities on all substrates. However, those variants exhibited a large decrease in catalytic activity compared to the wildtype activity. Interestingly, it could be seen how the two approaches targeted different regions of the enzyme. The vertical approach of ancestral sequence reconstruction showed mutations in the outer shells of the enzymes, leaving the active sites conserved like the wildtypes. This way the reconstructed sequences have likely increased overall flexibility of the enzyme while keeping the catalytic machinery intact. The horizontal approach with active site engineering on the contrary targeted exactly that catalytic machinery in order to adapt the reaction mechanism for the promiscuous reaction. Overall, the engineered variants exhibited a broader promiscuity landscape with more balanced specificities and activities on all three substrates, while the ancestor presented activities for just two substrates with a strong bias toward the native substrate but maintained more of the overall activity.

## 4 Experimental Section

### 4.1 GFN2‐xTB Cluster Model Analysis

To investigate the active site interactions, extended tight binding analysis was performed [21]. Geometry optimizations were carried out using GFN2‐xTB with the ALPB implicit solvation model using water as the solvent, keeping all other default settings. Structures of *Cn*KdaD and *Ab*KdgD were generated using AlphaFold2 via ColabFold [23]. The systems were built with the appropriate total charge depending on enzyme and substrate, for the wild‐type enzymes with their native substrates, the *Ab*KdgD–Kdg model contained 484 atoms (charge –2) and the *Cn*KdaD–Kda model contained 523 atoms (charge –3). The substrates were modeled into the active site based on the ligand orientations in the crystal structures of KdgD of *Agrobacterium fabrum* (PDB ID: 5HWJ, 5HWN, 5HWM) and KdaD of *Azospirillum brasilense* (PDB‐ID: 7C0C, 7C0D, 7C0E) using the builder tool in PyMol. For the analysis, only residues in contact or in close proximity to the ligand were considered, and the α‐carbons of all truncated residues were constrained. The model of *Ab*KdgD included the following residues: S16, F17, P18; L48, F49, A50; G52, G53, T54, G55, E56, F57, F58; I108, L109, L110, M111, P112; F140, Y141, N142, R143, S144; F166, K167, D168, S169, S170; G191, G192, L193, P194, T195, A196, E197, I198; Y211, S212, S213, A214, V215, F216, N217, F218; Y259, A260, V261, S262; and the model of *Cn*KdaD was made up of the following residues: P15, V16, A17; I47, C48, I49, L50, A51, N52, F53, S54, E55; V108, M109, V110, M111, P112; I141, M142, I143, Q144, D145; F171, K172, I173, E174, V175; W197, D198, G199, E200, E201, A202; A216, M217, T218, G219, A220, G221, F223, P224; Y254, E255, N256, R257; G259, W260, L261, and A262. Broken peptide bonds were capped with hydrogens, which were also restrained to preserve the enzyme environment during optimization. The most optimal substrate orientation was determined by iterative rounds of extended tight binding simulations until the interactions of the proposed catalytic mechanisms were achieved and the start and end positions of the simulation did not differ too much. The orientation of the native substrate was used as a template for the nonnative substrate. Binding simulations were carried out for the mutated variants as well with both Kdg and Kda. Only the residues of the active site residues were considered and the calculation took place with water as the selected solvent. Calculations were performed using the xTB program package (version 6.5.1) following the recommended procedure described in the official documentation [24]. Figures 4–6 show a summarized result of the analysis; more detailed depictions of the results can be found in the supplementary (Figures S27−S29).

### 4.2 Ancestral Sequence Reconstruction

Ancestral sequence reconstruction was started by gathering sequences of the DHDPS superfamily. UniProt blast and the Conserved Protein Domain Family entry of the DHDPS superfamily were used to identify sequences. The final dataset contained 25 KdgD sequences, 15 KdaD sequences, 5 KdgA sequences, 8 CHBPH sequences, 14 NAL sequences, 65 DHDPS sequences, and 10 sequences belonging to the DERA enzyme family used as an outgroup. The ratios of the sequences used per subfamily were chosen in an attempt to mimic the real distribution within the superfamily, but the DHDPS part was sized down from 75% entries in UniProt to 50% sequences in the dataset in order to allow for sequences of the other subfamilies without increasing the size of the whole dataset. The final dataset was aligned with the structural alignment tool Promals3D [10] due to the low sequence identity in the DHDPS superfamily. For the generation of the phylogenetic tree, the IQ‐TREE webserver [11] was chosen. The tree was calculated with a maximum likelihood algorithm and the LG + G substitution model. 1000 ultrafast bootstraps were applied to confirm the robustness of the tree. The reconstruction of the ancestral sequences was performed with the GRASP tool [12] using the joint reconstruction mode after supplementing the alignment and the phylogenetic tree alongside the site‐specific rates generated from IQ‐TREE.

### 4.3 Reagents

Components for media, buffers, and assay reactions were purchased from either Carl Roth, Thermo Scientific, or Sigma–Aldrich if not mentioned otherwise. 5‐Kdg, L‐Kda, D‐Kdx, and KGSA were synthesized in‐house, described elsewhere [7, 13]. Commercial enzymes for PCR or Gibson assembly were purchased from New England Biolabs.

### 4.4 Strains and Plasmids

Used strains are *Escherichia coli* (*E. coli*) DH5α and *E. coli* BL21(DE3). The enzymes were prepared in pET28a vectors. The ancestral sequences were purchased as DNA strings and cloned into the pET28a vector with or without an MBP tag depending on the enzyme. The variants of *Cn*KdaD and *Ab*KdgD were generated by site‐directed mutagenesis during PCR with the primers listed in Table S1. The PCR was carried out in NEB‐HF buffer for *Ab*KdgD and NEB‐GC buffer with 3% DMSO for *Cn*KdaD with an annealing temperature between 60–65 °C. PCR samples were analyzed via 1% agarose gel electrophoresis, and the corresponding target bands were cut from the gel and isolated using the PRC clean‐up kit (Macherey–Nagel). The plasmids were assembled via Gibson assembly (Iso reaction buffer (25% PEG‐8000, 500 mM Tris/Cl pH 7.5, 50 mM MgCl2, 50 mM DTT, 1 mM dATP, 1 mM dCTP, 1 mMdGTP, 1 mM dTTP, 5 mM NAD), T5 exonuclease, Phusion High‐Fidelity DNA Polymerase, and Taq DNA ligase), where 5 µL of DNA is incubated with 15 µL of Gibson master mix at 50 °C for 1 h. All used and generated plasmids of this work are listed in Table S2.

Chemically competent *E. coli* DH5 α cells were transformed with the assembled plasmids, and the introduced mutations were confirmed by Sanger sequencing. For the transformation, the cells were incubated with the plasmid for 30 min on ice followed by a heat shock at 42 for 45 °C. The cells were again cooled on ice for 5 min, after which they were diluted 1:10 with SOC media and incubated for 1 h at 37 °C with mild shaking. The cells were plated on LB agar plates containing 100 µg/mL Kanamycin and incubated at 37 °C over night or at room temperature for 72 h. For plasmid isolation, a culture of 5 mL LB media with 100 µg/mL Kanamycin was inoculated *E. coli* DH5 α cells containing the target plasmid. The culture was incubated at 37 °C, 300 rpm for 16 h, after which the cells were harvested (21000 g, 20 °C, 5 min), and plasmid isolation was carried out using a miniprep kit (Thermo Scientific).

### 4.5 Heterologous Expression and Protein Purification

For protein expression of the dehydratases, the plasmids were used to transform chemically competent *E. coli* BL21 (DE3) cells. The cells were given into a preculture of 20 mL LB medium with 100 µg/mL Kanamycin. The preculture was incubated at 37 °C and 150 rpm overnight and then used to inoculate 400 mL^–1^ L of autoinduction medium containing 100 µg/mL Kanamycin, 1 M sorbitol, and 5 mM betaine. The cultures are inoculated to an OD of 0.1 and then incubated at 30 °C for 24 h. The cells are harvested by centrifugation (20 min, 8000 g, 4 °C) and stored at −20 °C. *Pp*KGSADH was expressed similarly, expect the main culture was incubated at 37 °C for 3 h and at 18 °C for additional 21 h.

For purification, the cells were thawed and resuspended in 50 mM sodium phosphate buffer (pH 8, 500 mM NaCl, 10% glycerol). Cell lysis was achieved by sonication (15 min, 80%, 0.5), and the lysate was cleared by centrifugation (30 min, 45 000 g, and 4 °C) and subsequent filtration (0.2 µm). The lysate was applied to a 1 mL His‐tag column, and purification was carried out via an aekta purifier system. After loading of the lysate, the column was washed with 5 CV of the resuspension buffer before eluting the protein with a 0%–100% gradient of the elution buffer (50 mM sodium phosphate pH 8, 500 mM NaCl, 500 mM imidazole, 10% glycerol). The fractions were analyzed by a UV detector, and fractions containing protein were pooled and applied to a size exclusion column for buffer exchange. The protein was eluted in 50 mM HEPES buffer (pH 7.5, 500 mM NaCl, 10% glycerol) and flash frozen with liquid nitrogen and stored at −80 °C. For enzymes with a MBP‐tag, a TEV digest was performed to cleave off the tag. 2 mg of the tagged enzyme was incubated with 0.2 mg/mL TEV protease at 4 °C for at least 16 h. Afterward the solution was applied to a 1 mL nickel Sepharose column to filter off the MBP. The enzyme was washed of the column with 50 mM HEPES buffer (pH 7.5, 500 mM NaCl, 10% glycerol). Protein concentration was measured with a Nanodrop via UV signal using the molecular masses and extinction coefficients calculated using the expasy ProtParam webserver [25] (Table S3).

### 4.6 Enzyme Assays

To measure the activity of the dehydratases, the reaction was coupled to *Pp*KGSADH (EC 1.2.1.26; Uniprot A0A0P7CZ02), which uses the produced keto‐glutarate semialdehyde and NAD to from 2‐keto‐glutarate and NADH. The increase in NADH was measured spectrophotometrically at 340 nm over the course of 15 min at room temperature. Activity of *Pp*KGSADH was measured in 50 mM HEPES buffer (pH 7.5) with 5 mM MgCl2, 2.5 mM NAD, 0.01 ‐ 10 mM KGSA and 17 nM of *Pp*KGSADH. For the activity measurement of the dehydratases, the reaction was carried out in 50 mM HEPES buffer (pH 7.5) with 5 mM MgCl_2_, 2.5 mM NAD, 0.01 – 10 mM of substrate, 170 nM *Pp*KGSADH and 0.01–1 mg/mL of the target enzyme. MgCl_2_ was added to the reaction mixture as higher activities were measured in presence of the salt. For determination of kinetic parameters, Origin 2021b was used with the Michaelis‐Menten fit. The detection limit was determined by the background absorption signal of the substrates Kdg, Kda, and Kdx. Only absorption measurements, where the signal visibly surpassed the background, were further investigated and counted as activity.

## Funding

This work was supported by the Deutsche Forschungsgemeinschaft.

## Conflicts of Interest

The authors declare no conflicts of interest.

## Supporting information

Supplementary Material

## Data Availability

The data that support the findings of this study are available from the corresponding author upon reasonable request.
