# Supplementary material for: Investigation of Substrate Specificity in 5‐Keto‐4‐Deoxy‐Glucarate Dehydratase and 2‐Keto‐3‐Deoxy‐L‐Arabinonate Dehydratase
Source: Chembiochem. 2026 Jul 24;27(14):e70472. doi: 10.1002/cbic.70472 (PMC13398020; doi:10.1002/cbic.70472)
Supplement: Supplementary file 1 — Supplementary Material [file CBIC-27-e70472-s001.pdf]

Supporting Information

Investigation of substrate specificity in 5-Keto-4-deoxy-glucarate dehydratase and 2-Keto-3-deoxy-L-arabinonate dehydratase

*Kathrin Hörnschemeyer, Esther Pruna, Sílvia Osuna, Mikael Boden and Volker Sieber**

Gene Sequences and amino acid sequences of the reconstructed ancestral enzymes

Ancestor N1

ATGCTGGGTCTGCCGCTGGGCCTGAAAAAGCTGCGTGGTGTTATTGCAGTTCTGCCGACACCGTTTGATGAAGAT
GGTGAAGTTGATCTGGAAGGTCTGAAGAAACTGGTGGAATTTCTGATTGAACAGGGTGTTGATGGTATTTGCATT
CTGGGTAATTTTGGCGAACAGTTTGTTCTGACCGATGAAGAACGTAAAGAACTGATTAAAACCGTTCTGGAAGCA
GTTAATGGTCGTGTTCCGGTTATTGTTACCACCGGTCATTATAACACCCGTGTTGCAGTTGAACGTGCAAAATAT
GCCGAAGAACTGGGTGCAGATGGTGTTATGGTTGTTCCGCCTTATCATGGTCCGCTGCGTGTGCCGGAAGAGGGT
ATTTATGAATTTTTCAAAGCAGTTAGCGAAGCCGTTGATCTGCCGATTATGATTTATGATATTCCGGCAGCAAGC
GGTGTTGATCTGAGCGCACCGACACTGGCACGTATGGCAAAAATTCCGAATGTTAGCTATGTGAAAATTACCACC
GCAGGCGCACTGGATCAGCTGCGTGAACTGATTGAATTAGGTGGTGATGCAATTGGTGTTTTTAGCGGTGGTGAA
GAACTGCTGCTGCCTGCGCTGCTGCTGGGTGCAACCGGTGCAATGACCGGCACCAGCGGTGTGGTGCCGGAACTG
GTTCGTCCGATTATTGAAGCATTTCTGGAAGGCGATCTGGAAAAAGCCGTTAAACTGTATCAAGAAGTGCTGCCG
CTGATCGAATATCTGAATAAACAGTGTGGTCTGAGCCTGGTTAAAGCAGCAATGCAAGAAGGTGGCGTTATTAAA
GCAGGTCCGTGTCGTCCGCCTTTTCCGCCTCTGCATCCGGAACAGCTGGAAGGCCTGCTGGAACTGCTGAAAAAG
TTAGGTCTGCTGCTGTTAAAATGGGGTCGTTAA

MLGLPLGLKKLRGVIAVLPTPFDEDGEVDLEGLKKLVEFLIEQGVDGICILGNFGEQFVLTDEERKELIKT
VLEAVNGRVPVIVTTGHYNTRVAVERAKYAEELGADGVMVVPPYHGPLRVPEEGIYEFFKAVSEAVDL
PIMIYDIPAASGVDLSAPTLARMAKIPNVSYVKITTAGALDQLRELIELGGDAIGVFSGGEELLLPALLLGA
TGAMTGTSGVVPELVRPIIEAFLEGDLEKAVKLYQEVLPLIEYLNKQCGLSLVKAAMQEGGVIKAGPCR
PPFPPLHPEQLEGLLELLKKLGLLLLKWGR*

Ancestor N2

ATGCTGGGTCTGCCGAGCAGCACCAAACGTTATCGTGGTGTTTTTCCGGTTGCACCGACACCGTTTGATGAAGAT
GGTGAACTGGATCTGGAAGGTCTGAAACGTGTTGTGGATTTTATGATTGATCAGGGCGTTGATGGTATTTGCATT
CTGGCAAATTTTAGCGAACAGTTTGTTCTGTCTGATGATGAACGTGAAGTTCTGACCAAAACCGTTCTGGAACAT
GTTGCAGGTCGTGTTCCGGTTATTGTTACCACCAGTCATTATAGCACCCGTGTTGCAGTTGAACGTGCCCGTTAT
GCACAGGATCTGGGTGCAGATATGGTTATGGTGATGCCTCCGTATCATGGTGCACTGTTTCGTGTGCCGGAAGAA
CAAATCTATGAATTTTTCAAAGCAGTGAGCGAAGCCGTGGATATTCCGATTATGATTCAGGATGCACCGGTTAGC
GGTGTTGATCTGAGCGCACCGTTTCTGGCACGTATGGCACGTGAAATTGAAAATGTGAGCTACTTCAAAATTGAA
ACCGGTGCCGCAAATAAACTGCGTGAACTGATTGAATTAGGTGGTGATGCAATTGGTGGTTTTAGCGGTGGTGAA
GAAGTTATTCTGCCTGCACTGCTGGCAGGCGCAACAGGTGCAATGACCGGCACCAGCGGTGTTATTCCGGATCTG
ATTCGTCCGATTATTGATGCATTTTTAGCCGGTGATCGTGAAAAAGCAGTTGCACTGTATCAGCAGGTTCTGCCG
CTGATTAACTATGAAAATCGTCAGTGTGGTCTGAGCACCTGTAAAGCAGCAATGCAAGAAGGTGGCGTTATCAAA
AGTGATGCATGTCGTCATCCGTTTCCGCCTCTGCATCCGGCAACACGTGAAGGTCTGCTGGAACTGCTGCGTCGT
CTGGATCCGCTGGTTCTGAAATGGGGTCGTTAA

MLGLPSSTKRYRGVFPVAPTPFDEDGELDLEGLKRVVDFMIDQGVDGICILANFSEQFVLSDDEREVLT
KTVLEHVAGRVPVIVTTSHYSTRVAVERARYAQDLGADMVMVMPPYHGALFRVPEEQIYEFFKAVSEA
VDIPIMIQDAPVSGVDLSAPFLARMAREIENVSYFKIETGAANKLRELIELGGDAIGGFSGGEEVILPALL
AGATGAMTGTSGVIPDLIRPIIDAFLAGDREKAVALYQQVLPLINYENRQCGLSTCKAAMQEGGVIKSD
ACRHPFPPLHPATREGLLELLRRLDPLVLKWGR*

Ancestor N3

ATGACCCGTCCGAGCAGCACACCGCGTTATCGTGGTGTTTTTCCGGTTGCACCGACCATTTTTGATGAAGATGGT
GAACTGGATCTGGAAGGTCAGAAACGTTGTGTGGATTTTATGATTGATGCAGGTTCAGATGGTCTGTGTATTCTG
GCAAATTTTAGCGAACAGTTTGTTCTGTCTGATGATGAACGTGAAGTTCTGACCAAAACCGTTCTGGAACATGTT
GCAGGTCGTGTTCCGGTTATTGTTACCACCACACATTATAGCACCCGTGTTTGTGCAGAACGTAGCCGTCGTGCA
CAGGATATGGGTGCAGCAATGGTTATGGTTATGCCTCCGTATCATGGTGCAACCTTTCGTGTGCCGGAAAAGCAG
ATTTATGAATTTTTCAAACGTGTTAGCGACGCCGTGGATATTCCGATTATGATTCAGGATGCACCGGTTAGCGGT
ACACCGCTGAGCGCACCGTTTCTGGCACGTATGGCACGTGAAATTGAAAATGTGAGCTACTTCAAAATTGAAACC
GCAGGCGCAGCAAGCAAACTGCGTGAACTGATTGAATTAGGTGGTGATGCAATTGAAGGTCCGTGGGATGGTGAA
GAAGCAATTACCCTGCTGCCGGATCTGGATGCCGGTGCCACCGGTGCAATGACAGGTGGTGGTTATCCGGATGGT
ATTCGTCCGATTATTGATGCATATCTGGCAGGCGATCGTGAAAAAGCAGTTGCACTGTATCAGCAGTGGCTGCCG
CTGATTAACTATGAAAATCGTCAGTGTGGTCTGAGCACCTGTAAAGCACTGATGCAAGAAGGTGGCGTTATCAAA
AGTGATGCAGTTCGTCATCCGTTTCCGCCTCTGCATCCGGCAACACGTGAAGGTCTGCTGGAAATTGCACGTCGT
CTGGATCCGCTGGTTCTGCGTTGGGGTCGTTAA

MTRPSSTPRYRGVFPVAPTIFDEDGELDLEGQKRCVDFMIDAGSDGLCILANFSEQFVLSDDEREVLT
KTVLEHVAGRVPVIVTTTHYSTRVCAERSRRAQDMGAAMVMVMPPYHGATFRVPEKQIYEFFKRVSD
AVDIPIMIQDAPVSGTPLSAPFLARMAREIENVSYFKIETAGAASKLRELIELGGDAIEGPWDGEEAITLL
PDLDAGATGAMTGGGYPDGIRPIIDAYLAGDREKAVALYQQWLPLINYENRQCGLSTCKALMQEGGVI
KSDAVRHPFPPLHPATREGLLEIARRLDPLVLRWGR*

<u>Ancestor N4</u>

ATGCTGGGTCTGCCGAGCAGCACCCCTCCGTATCGTGGTGTTTTTCCGGTTGCACCGACCATTTTTGATGAAGAT
GGTGAACTGGATCTGGAAGGTCAGAAACGTTGTATCGATTTTATGATTGATGCCGGTAGCAATGGTCTGTGTATT
CTGGCAAATTTTAGCGAACAGTTTGTTCTGTCTGATGATGAACGTGAAGTTCTGACCAAAACCGTTCTGGAACAT
GTTGCAGGTCGTGTTCCGGTTATTGTTACCACCACACATTATAGCAGCCGTGTTTGTGCAGAACGTAGCCGTCGT
GCACAGGATGCGGGTGCAGCAATGGTTATGGTTATGCCACCGTATCATGGTGCAACCTTTCGTGTGCCGGAAAAG
CAGATTTATGAATTTTTCAAACGTGTGAGCGACGCCATTGATATTCCGATTATGATTCAGGATGCACCGGTTAGC
GGTACACCGCTGAGCGCACCGTTTCTGGCACGTATGGCACGTGAAATTGAAAATGTGAGCTACTTCAAAATTGAA
ACCGCAGGCGCAGCAAGCAAACTGCGTGAACTGATTGAATTAGGTGGTGATGCAATTGAAGGTCCGTGGGATGGT
GAAGAAGCAATTACCCTGCTGCCGGATCTGGATGCCGGTGCCACCGGTGCAATGACAGGTGGTGGTTATCCGGAT
GGTATTCGTCAGATTATTGATGCATATCTGGCAGGCGATCGTGAAAAAGCAGTTGCACTGTATCAGCAGTGGCTG
CCGCTGATTAACTATGAAAATCGTCAGTGTGGTCTGAGCACCTGTAAAGCACTGATGCAAGAAGGTGGCGTTATC
AAAAGTGATGCAGTTCGTCATCCGCTGCCTCCGCTGCATCCGGCAACACGTGAAGGTCTGCTGGAAATTGCACGT
CGTCTGGATCCGCTGGTTCTGCGTTGGGGTCGTTAA

MLGLPSSTPPYRGVFPVAPTIFDEDGELDLEGQKRCIDFMIDAGSNGLCILANFSEQFVLSDDEREVLT
KTVLEHVAGRVPVIVTTTHYSSRVCAERSRRAQDAGAAMVMVMPPYHGATFRVPEKQIYEFFKRVSD
AIDIPIMIQDAPVSGTPLSAPFLARMAREIENVSYFKIETAGAASKLRELIELGGDAIEGPWDGEEAITLLP
DLDAGATGAMTGGGYPDGIRQIIDAYLAGDREKAVALYQQWLPLINYENRQCGLSTCKALMQEGGVIK
SDAVRHPLPPLHPATREGLLEIARRLDPLVLRWGR*

<u>Ancestor N5</u>

ATGACCCGTCCGAGCAGCACCCCTCCGTATCGTGGTGTTTTTCCGGTTGCACCGACCATTTTTGATGAACAGGGT
GAACTGGATCTGGAAGGTCAGAAACGTTGTATCGATTTTATGATTGATGCAGGTAGCCATGGTCTGTGTATTCTG
GCAAATTTTAGCGAACAGTTTGTTCTGTCTGATGATGAACGTGAAGTTCTGATGAAAACCGTTCTGGAACATGTT
GCAGGTCGTGTTCCGGTTATTGTTACCACCACACATTTTAGCAGCCGTGTTTGTGCAGAACGTAGCCGTCGTGCA
CAGGATGCCGGTGCAGCAATGGTTATGGTTATGCCACCGTATCATGGTGCAACCTTTCGTGTGCCGGAAAAAGGT
ATTTATGAATTCTTTAAACGCGTGAGCGACGCCATTGATATTCCGATTATGATTCAGGATGCACCGGTTAGCGGT
ACACCGCTGAGCGCACCGTTTCTGGCACGTATGGCACGTGAAATTGCAAATGTTAGCTACTTCAAAATCGAAGTT
CCGGGTGCAGCCAGCAAACTGCGTGAACTGATTGAATTAGGTGGTGATGCAATTGAAGGTCCGTGGGATGGTGAA
GAAGCAATTACCCTGCTGGCAGATCTGGATGCGGGTGCCACCGGTGCCATGACCGGTGGTGGTTATCCGGATGGT
ATTCGTCAGATTATTGATGCATATCTGGCAGGCGATCGTGAAAAAGCAGTTGCAGCATATCAGCAGTGGCTGCCG
CTGATTAACTATGAAAATCGTCAGTGTGGTCTGGCCACCTGTAAAGTGCTGATGCAAGAAGGTGGCGTTATCAAA
AGTGATGCAGTTCGTCATCCGCTGCAGCCGCTGCATCCGGCAACACGTGAAGGTCTGCTGGAAATTGCACGTCGT
CTGGATCCGCTGGTTCTGCGTTGGGGTCGTTAA

MTRPSSTPPYRGVFPVAPTIFDEQGELDLEGQKRCIDFMIDAGSHGLCILANFSEQFVLSDDEREVLM
KTVLEHVAGRVPVIVTTTHFSSRVCAERSRRAQDAGAAMVMVMPPYHGATFRVPEKGIYEFFKRVSD
AIDIPIMIQDAPVSGTPLSAPFLARMAREIANVSYFKIEVPGAASKLRELIELGGDAIEGPWDGEEAITLLA
DLDAGATGAMTGGGYPDGIRQIIDAYLAGDREKAVAAYQQWLPLINYENRQCGLATCKVLMQEGGVIK
SDAVRHPLQPLHPATREGLLEIARRLDPLVLRWGR*

<u>Ancestor N9</u>

ATGCCGAGCACCACCAGTAGCACACCGCGTTATCGTGGTATTTTTCCGGTTGTTCCGACCACCTTTACCGAAGAT
GGTGAACTGGATCTGGAAAGCCAGAAACGTTGTGTGGATTTTATGATTGATGCAGGTTCAGATGGTCTGTGTATT
CTGGCAAATTTTAGCGAACAGTTTGTTCTGTCTGATGATGAACGTGAAGTTCTGACCCGTACCATTCTGGAACAT
GTTGCAGGTCGTGTTCCGGTTATTGTTACCACCACACATTATAGCACCCGTGTTTGTGCAGAACGTAGCCGTCGT
GCACAGGATATGGGTGCAGCAATGGTTATGGTTATGCCTCCGTATCATGGTGCAACCTTTCGTGTGCCGGAAGCA
CAGATTTATGAATTTTATGCACGTGTTAGTGATGCCGTGGATATTCCGATTATGATTCAGGATGCACCGGCAAGC
GGCACCGTTCTGAGCGCACCTTTTCTGGCACGTATGGCACGTGAAATTGAACATGTGGCCTATTTCAAAATTGAA
ACCGCAGGCGCAGCAAGCAAACTGCGTGAACTGATTCGTTTAGGTGGTGATGCAATTGAAGGTCCGTGGGATGGT
GAAGAAGCAATTACCCTGCTGCCGGATCTGGATGCGGGTGCAACCGGTGCAATGACCGGTGGTGGTTATCCGGAT
GGTATTCGTCCGATTATTGAAGCACATCGTGCCGGTGATCGTGATAAAGCATTTGCACTGTATCAGCAGTGGCTG
CCGCTGATTAACTATGAAAATCGTCAAGGTGGTATCCTGACCTGTAAAGCACTGATGAAAGAGGGTGGCGTTATT
GCATGTGAAGCACCGCGTCATCCGTTTCCGGCAATGCATCCAGAAACACGTGCCGGTCTGCTGGAAATTGCACGT
CGTCTGGATCCGCTGGTTCTGCGTTGGGGTAAATAA

MPSTTSSTPRYRGIFPVVPTTFTEDGELDLESQKRCVDFMIDAGSDGLCILANFSEQFVLSDDEREVLT
RTILEHVAGRVPVIVTTTHYSTRVCAERSRRAQDMGAAMVMVMPPYHGATFRVPEAQIYEFYARVSDA
VDIPIMIQDAPASGTVLSAPFLARMAREIEHVAYFKIETAGAASKLRELIRLGGDAIEGPWDGEEAITLLP
DLDAGATGAMTGGGYPDGIRPIIEAHRAGDRDKAFALYQQWLPLINYENRQGGILTCKALMKEGGVIA
CEAPRHPFPAMHPETRAGLLEIARRLDPLVLRWGK*

Ancestor N12

ATGACCAAAACCTATACCGGCATTTGGCCTGTTGCACCGACACCGTTTAATCCGGATGGCACCCTGGATCTGGAA
GGTATGAAACGTGTTCTGGATTGTATGATTGATCAGGGTGTTGATGGTATTTGCATTCTGGCAAATTTTAGCGAA
CAGTTTCTGATCTCAGATGCCGAACGTGAAGTTCTGACCCGTCTGAGCCTGGAACATGTTGCCGGTCGTGTTCCG
GTTATTGTTACCATTAGCCATTATGCAACCCAGATTGCAGTTGAACGTGCACAGTTTGCAAAAGATCTGGGTGCA
GATATTGTTATGATGATGCCTCCGTATCATGGTGCACTGCTGAAAGGCACCGCAGAACAGACCTTTGAACAGTTT
AAAGCAGTTGGTGAAGTGGGCATTCCGATTATGGTTCAGGATGCACCGCTGAGTGGTGTTGATCTGCCGGTTCCG
CTGCTGGTTCGTATGGCACGTGAAATTGAAATGGTGAAGCTGTTCAAAATTGAATGTCCGCGTGCAGCAAATAAA
CTGCGTGCACTGATTGAACAAGGTGGTGATGCAATTGAAGGTCCGTTTGATGGTGAAGAAGCAATTACCCTGCTG
GCAGATCTGGATGCCGGTGCAACCGGTACAATGACCAGCGGTATGATTCCGGATCTGATTAAACCGGTTGTTACC
GATTTTCTGGCAGGTAATCGTGAAGAGGCCATTGCAGCATATGCCCGTGTTCTGCCTGCAATTAATCATGAAAAT
CGTCAGTGTGGTTTTCGTAGCTGTAAAGCAGCAATGGTTGAAGGTGGTGTGATCAAAAGCGATTTTTGTCGTCAT
CCGATTCCGCCTCTGCATCCGGCAACACGTGAACGTCTGATGGAACTGCTGCGTCCGCTGGATCCGATGGTTCTG
AAATGGGGTAAATAA

MTKTYTGIWPVAPTPFNPDGTLDLEGMKRVLDCMIDQGVDGICILANFSEQFLISDAEREVLTRLSLEH
VAGRVPVIVTISHYATQIAVERAQFAKDLGADIVMMMPPYHGALLKGTAEQTFEQFKAVGEVGIPIMVQ
DAPLSGVDLPVPLLVRMAREIEMVKLFKIECPRAANKLRALIEQGGDAIEGPFDGEEAITLLADLDAGAT
GTMTSGMIPDLIKPVVTDFLAGNREEAIAAYARVLPAINHENRQCGFRSCKAAMVEGGVIKSDFCRHPI
PPLHPATRERLMELLRPLDPMVLKWGK*

Ancestor N16

ATGCTGGGTCTGCCGCTGGGCCTGAAAAAGCTGCGTGGTGTTATTGCAGCACTGCCGACACCGTTTGATGAAGAT
GGTGAAGTTGATCTGGAAGCACTGCGTAAACTGGTTGAATTTCTGATTGAACAGGGTGTCGATGGTATTTTTGTT
AGCGGCACCTTTGGTGAAAGCTTTACCCTGACACATGAAGAACGTAAAGAACTGATTAAAACCGTTGTGGAAGCA
GTTAATGGTCGTGTTCCGGTTATTGTTGGCACCGGTAGCCTGAATACCCGTGAAGCAATTGAACGTGCAAAATAT
GCCGAAGAACTGGGTGCAGATGGTGTTATGGTTGTTCCGCCTTATTATAGTCCGCTGAGCGAAGAAGGTATCTAC
CAGTATTTCAAAGCAGTTGCAGAAGCCGTTGATCTGCCGATTATGATTTATGATATTCCGGCAGCAAGCGGTGTT
AATCTGAGCCCTCCGACCTTTGCACGTCTGGCAAAAATTCCGAATGTTGTTGGTGTTAAACATACCAGCGGTGAT
CTGGATCAGCTGGCAGAAATTATTGAACTGACCGGTGATGATTTTGCCGTTTTTAGCGGTAGTGATGAACTGCTG
CTGCCTGCGCTGCTGCTGGGTGCAACCGGTGCAATTAGCGGTACAAGCAATGTTGTGCCGGAACTGGTTCGTGAA
ATGATTGAAGCATTTCAAGAAGGCGATCTGGAAAAAGCACGTAAACTGCATCATGAAATCCTGCCGCTGATCGAA
GCACTGTTCAAACAGGGTAATCTGAGTCTGGTTAAAGCAGCAATGCAGCTGATGGGTGTTGTTAAAGCCGGTCCG
TGTCGTCCGCCTTTTGTTCCGCTGTCAGAAGAACAGCTGGAAGGTCTGCAAGAACTGCTGAAGAAACTGGGCCTG
CTGCTGTAA

MLGLPLGLKKLRGVIAALPTPFDEDGEVDLEALRKLVEFLIEQGVDGIFVSGTFGESFTLTHEERKELIK
TVVEAVNGRVPVIVGTGSLNTREAIERAKYAEELGADGVMVVPPYYSPLSEEGIYQYFKAVAEAVDLPI
MIYDIPAASGVNLSPPTFARLAKIPNVVGVKHTSGDLDQLAEIIELTGDDFAVFSGSDELLLPALLLGATG
AISGTSNVVPELVREMIEAFQEGDLEKARKLHHEILPLIEALFKQGNLSLVKAAMQLMGVVKAGPCRPP
FVPLSEEQLEGLQELLKKLGLLL*

Ancestor N24

ATGCTGGGTCTGCCGCTGGGCCTGAAAAAGCTGCGTGGTGTTATTGCAGCACTGGTTACCCCGTTTGATGAAGAT
GGTGAAGTTGATCAAGAAGCACTGCGTAAACTGGTGGAATTTCTGATTGAACAGGGTGTTGATGGTATTTTTGTT
AGCGGCACCACCGGTGAAAGCTTTACCCTGACACATGAAGAACGTAAAGAACTGATTAAAACCGTTGTGGAAGCA
GTTAATGGTCGTGTTCCGGTTATTGCCGGTACAGGTAGCCTGAATACCCGTGAAGCAATTGAACTGGCAAAATAT
GCCGAAGAACTGGGTGCAGATGGTGTTATGGTTGTTCCGCCTTATTATAGTCCGCTGAGCGAAGAAGGTATCTAC
CAGTATTTCAAAGCAGTTGCAGAAGCAGCCGATCTGCCGATTATTATCTATAACATTCCGGCACGTACCGGTGTT
AATCTGAGTCCGGAAACCTTTGCACGTCTGGCCAAAATTCCGAATATTGTTGGTGTTAAACATACCAGCGGTGAT
CTGGATCAGCTGGCAGAAATTATTGAACTGACCGGTGATGATTTTGCCGTTTTTAGCGGTAGTGATGAACTGCTG
CTGCCTGCGCTGCTGCTGGGTGCAACCGGTGCAATTAGCGCAACCAGCAATGTTGTGCCGGAACTGGTTCGTGAA
ATGATTGAAGCATTTCAAGAAGGCGATCTGGAAAAAGCACGTAAACTGCATCATGAAATCCTGCCGCTGATCGAA
GCACTGTTCAAACAGGGTAATCTGAGCCTGGTTAAAGCAGCAATGCAGCTGATGGGTGTTGTTAAAGCCGGTCCG
TGTCGTCCGCCTTTTGTTCCGCTGTCAGAAGAACAGCTGGAAAAACTGCAAGAACTGCTGAAGAAACTGGGCCTG
CTGCTGTAA

MLGLPLGLKKLRGVIAALVTPFDEDGEVDQEALRKLVEFLIEQGVDGIFVSGTTGESFTLTHEERKELIK
TVVEAVNGRVPVIAGTGSLNTREAIELAKYAEELGADGVMVVPPYYSPLSEEGIYQYFKAVAEAADLPIII
YNIPARTGVNLSPETFARLAKIPNIVGVKHTSGDLDQLAEIIELTGDDFAVFSGSDELLLPALLLGATGAIS
ATSNVVPELVREMIEAFQEGDLEKARKLHHEILPLIEALFKQGNLSLVKAAMQLMGVVKAGPCRPPFVP
LSEEQLEKLQELLKKLGLLL*

Ancestor N43

ATGCTGGGTCTGCCGCTGGGTCAGATGATGCTGCGTGGTGTTATTACCGCACTGGTTACCCCGTTTGATGAAGAT
GGTGAAGTTGATCAAGAAGCACTGCGTAAACTGGTGGAATTTCTGATTGAAAATGGTGTGGATGGCATTTTTGTT
AGCGGCACCACCGGTGAAAGCTTTACCCTGACACATGAAGAACATAAAGAGCTGATTAAGACCGTTGTTGAAGCA
GTTAATGGTCGTGTTCCGGTTATTGCAGGTACAGGTAGCCTGAATACCCGTGAAGCAATTGAACTGGCAAAATAT
GCCGAAGAGGTTGGTGCAGATGGTGTTCTGGTTGTTCCGCCTTATTATAGCAAACCGAGCCAAGAAGGTCTGTAT
CAGCATTTTAAAGCAGTTGCAGAAGCAGCCGATCTGCCGATTATTATCTATAACATTCCGGGTCGTACCGGTGTT
AATCTGAGTCCGGAAACCATTGCACGTCTGGCCGAAATTCCGAATATTGTTGGTGTTAAAGAAGCATCAGGCGAT
CTGGATCAGCTGGCAGAAATTATTGAACTGACCGGTGATGATTTTGCCGTTTTTAGCGGTAGCGATAGTCTGACC
CTGCCGATGCTGGCACTGGGTGCAACCGGTGTGATTAGCGCAACCAGCAATGTTGTGCCGGAACTGGTTCGTGAA
ATGATTGAAGCATTTCAAGAAGGCGACCTGGAAAAAGCACGTAAACTGCATCATGAACTGCTGCCGCTGTTTGAA
GCCCTGTTCAAACAGGGTAATCTGAGCCTGGTTAAAGCAGCAATGCAGCTGATGGGTTTAGTTGCAGGTCCGCTG
CGTCCGCCTCTGGTTCCGCTGAGCGAAGAACAGCTGGAAAAACTGCAAGAACTGCTGAAGAAACTGGGCCTGCTG
CTGTAA

MLGLPLGQMMLRGVITALVTPFDEDGEVDQEALRKLVEFLIENGVDGIFVSGTTGESFTLTHEEHKELIK
TVVEAVNGRVPVIAGTGSLNTREAIELAKYAEEVGADGVLVVPPYYSKPSQEGLYQHFKAVAEAADLPII
IYNIPGRTGVNLSPETIARLAEIPNIVGVKEASGDLDQLAEIIELTGDDFAVFSGSDSLTLPMLALGATGVI
SATSNVVPELVREMIEAFQEGDLEKARKLHHELLPLFEALFKQGNLSLVKAAMQLMGLVAGPLRPPLVP
LSEEQLEKLQELLKKLGLLL*

Ancestor N44

ATGAATCCGCTGGAACTGCGTCAGACCGTTCCGAGCGGTGTTCTGAGCTTTCCGGTTACACCGTTTGATGCAGAT
GGTGAATTTGATGAAGATGCCCTGCGTAAACATGTTGAATTTCTGCTGGAAGATGGTGCCGAAGCAATTTTTGTT
GCATGTGGCACCGGTGAATTTTTCAGCCTGACACCGGATGAATATCCGCAGGTTGTTCGTGCAGCAGTTGAAGCA
ACCGCAGGTCGTGTTCCGGTTATTGCCGGTGCAGGTAGCAATACCGCACAGGCAATTGAATATGCACAGCTGGCA
GAAAAAGCCGGTGCCGATGGTATTCTGGTTCTGCCTCCGTATCTGACCGAAGCAAGCCAAGAGGGTCTGTATCAG
CATGTTAAAGCAGTTGCAGAAAGCACCAATCTGCCGGTGATTGTTTATAATCGTGATAATGCAGTTCTGACTCCG
GAAACCATTGCACGTCTGGCAGAATGTCCGAATCTGGTTGGTGTTAAAGATGGTGTTGGTGATCTGGATCTGATG
ACCCGTATTGTTGCACTGCTGGGTGATCGTTTTACCTATTTTAACGGTCTGCCGACCGCAGAAACCACCGCACTG
CCGTATCTGGCACTGGGTGTTACCAGCTATAGCAGCGCAGTTAGCAATTTTGTGCCGGAACTGGCACGTGATTTT
TATCGTGCACTGCGTGAAGGTGATGATGAAACCGTTCATCGTCTGCATCGTGATTTCTTTCTGCCGTTTGTTGCA
ATTCGCAAACAAGGTAAAGGTTATGCAGTTAGCCTGGTTAAAGCCGGTATGAAACTGATGGGTTTAGATGCAGGT
CCGGTTCGTCCGCCTCTGACACCGCTGACAGAAGAAGAACTGGAACAACTGGCAGCACTGCTGAAGAAAGCGGGT
GTTCGTGTTTAA

MNPLELRQTVPSGVLSFPVTPFDADGEFDEDALRKHVEFLLEDGAEAIFVACGTGEFFSLTPDEYPQV
VRAAVEATAGRVPVIAGAGSNTAQAIEYAQLAEKAGADGILVLPPYLTEASQEGLYQHVKAVAESTNLPV
IVYNRDNAVLTPETIARLAECPNLVGVKDGVGDLDLMTRIVALLGDRFTYFNGLPTAETTALPYLALGVT
SYSSAVSNFVPELARDFYRALREGDDETVHRLHRDFFLPFVAIRKQGKGYAVSLVKAGMKLMGLDAG
PVRPPLTPLTEEELEQLAALLKKAGVRV*

Ancestor N45

ATGAATCCGCTGGAACTGCGTCAGACCGTTCCGAGCGGTGTTCTGAGCTTTCCGGTTACACCGTTTGATGCAGAT
GGTGAATTTGATGAAGATGCCTATCGTAAACATGTTGAATGGCTGCTGGAAGATGGTGCAGCAGCACTGTTTGCA
GCATGTGGCACCGGTGAATTTTTCAGCCTGACACCGGATGAATATCCGCAGGTTGTTCGTGCAGCAGTTGAAGCA
ACCGCAGGTCGTGTTCCGGTTATTGCCGGTGCAGGTTATGGCACCGCACAGGCAATTGAATATGCACAGCTGGCA
GAAAAAGCCGGTGCCGATGGTATTCTGGTTCTGCCTCCGTATCTGACCGAAGCAAGCCAAGAGGGTCTGTATGCA
CATGTTAAAGCAGTTGCAGAAAGCACCAATCTGCCGGTGATTGTTTATAATCGTGATAATGCAGTTCTGACTCCG
GAAACCATTGCACGTCTGGCAGAATGTCCGAATCTGGTTGGTCTGAAAGATGGTGTTGGTGATCTGGATCTGATG
ACCCGTATTGTTGCACTGCTGGGTGATCGTTTTACCTATTTTAACGGTCTGCCGACCGCAGAAACCACCGCACTG
CCGTATCTGGCACTGGGTGTTACCAGCTATAGCAGCGCAGTTTTTAACTTTGTGCCGGAACTGGCCCTGGATTTT
TATCGTGCACTGCGTGAAGGTGATGATGCAACCGTTCATCGTCTGCTGCGTGATTTCTTTCTGCCGTTTGTTGCA
ATTCGTAATCGCGGTAAAGGTTATGCAGTTAGCCTGGTTAAAGCCGGTATGAAACTGATGGGTTTAGATGCAGGT
CCGGTTCGTCCTCCGCTGACCGATCTGACAGAAGAAGAACTGGAACAACTGGCAGCACTGATTAAGAAAGCGGGT
GTTCGTTAA

MNPLELRQTVPSGVLSFPVTPFDADGEFDEDAYRKHVEWLLEDGAAALFAACGTGEFFSLTPDEYPQ
VVRAAVEATAGRVPVIAGAGYGTAQAIEYAQLAEKAGADGILVLPPYLTEASQEGLYAHVKAVAESTNLP
VIVYNRDNAVLTPETIARLAECPNLVGLKDGVGDLDLMTRIVALLGDRFTYFNGLPTAETTALPYLALGV
TSYSSAVFNFVPELALDFYRALREGDDATVHRLLRDFFLPFVAIRNRGKGYAVSLVKAGMKLMGLDAG
PVRPPLTDLTEEELEQLAALIKKAGVR*

Ancestor N46

ATGAATCCGCAAGAACTGAAACAGACCGTTAGCAGCGGTCTGCTGAGCTTTCCGGTTACACATTTTGATGCAGAT
GGCGAATTTGATGAAGCAGCCTATCGTAAACATGTTGAATGGCTGAGCGGTTATGGTGCAGCAGCACTGTTTGCA
GCCGGTGGCACCGGTGAATTTTTCAGCCTGACACCGGATGAATATCCGCAGGTTGTTCGTGCAGCAGTTGAAGCA
ACCGCAGGTCGTGTTCCGGTTATTGCCGGTGCAGGTTATGGCACCGCACAGGCAATTGAATATGCACAGGCAGCA
GAAAAAGCGGGTGCAGATGGTATTCTGCTGCTGCCTCCGTATCTGACCGAAGCAAGCCAAGAGGGTCTGTATGCA
CATGTTAAAGCAGTTTGTGAAAGCACCAATCTGGGTGTGATTGTGTATAATCGTGATAATGCAGTTCTGACTCCG
GATACCATTGCACGTCTGGCAGAACGTTGTCCGAATCTGGTTGGTTTTAAAGATGGTGTGGGTGATATTGAACTG
ATGACCCGTATTACCGCAAAACTGGGTGATCGTTTTACCTATTTAGGTGGTCTGCCGACCGCAGAAACCTATGCA
CTGCCGTATCTGGCACTGGGTGTTACCACCTATAGCAGCGCAGTTTTTAACTTTGTGCCGGAACTGGCCCTGGAT
TTTTATCGTGCAGTTCGTGCCGGTGATCATGCAACCGTTCATCGTCTGCTGCGTGATTTCTTTCTGCCGTTTGTT
GCAATTCGTAATCGCAAAAAGGGTTATGCCGTTAGCATTGTTAAAGCCGGTATGAAAGTTATTGGTCATGATGCA
GGTCCGGTTCGTCCTCCGCTGACCGATCTGACAGAAGAGGAACTGGAAGAACTGGCAGCACTGATTAAGAAAGCC
GGTGTTCGTTAA

MNPQELKQTVSSGLLSFPVTHFDADGEFDEAAYRKHVEWLSGYGAAALFAAGGTGEFFSLTPDEYPQ
VVRAAVEATAGRVPVIAGAGYGTAQAIEYAQAAEKAGADGILLLPPYLTEASQEGLYAHVKAVCESTNL
GVIVYNRDNAVLTPDTIARLAERCPNLVGFKDGVGDIELMTRITAKLGDRFTYLGGLPTAETYALPYLAL
GVTTYSSAVFNFVPELALDFYRAVRAGDHATVHRLLRDFFLPFVAIRNRKKGYAVSIVKAGMKVIGHDA
GPVRPPLTDLTEEELEELAALIKKAGVR*

Ancestor N47

ATGAATCCGCAAGAGCTGAAAAACATTGTTAGCAGCGGTCTGCTGAGCTTTCCGGTTACCGATTTTGATGCAAAT
GGTGATTTTAACGCAGCCAGCTATGCAAAACGTCTGGAATGGCTGGCACCGTATGGTGCAAGCGCACTGTTTGCA
GCCGGTGGCACCGGTGAATTTTTCAGCCTGACCGGTGATGAATATAGCGAAGTGATTAAAACCGCAGTTGATACC
TGTCGTGGTAGCGTTCCGATTATTGCCGGTGCCGGTGGTCCGACACGTCAGGCAATTGAATATGCACAAGAGGCA
GAACGTCTGGGTGCACATGGTATTCTGCTGCTGCCGCATTATCTGACCGAAGCAAGCCAAGAGGGTCTGATTGCA
CATGTTAAAGCAGTTTGCAAAAGCGTGAATTTTGGCGTGATTGTGTATAATCGTAATGTGTGTCGTCTGACACCG
GATAGCCTGGCAAAACTGGCCGAACGTTGTCCGAATCTGATTGGTTTTAAAGATGGTGTGGGTGATATTGAACTG
ATGACCAGCATTACCCAGAAACTGGGTGATCGTTTTAGCTATTTAGGTGGTCTGCCGACAGCCGAAGTTTATGCA
GCACCGTATAAAGCACTGGGTGTTCCGGTTTATAGCAGCGCAGTTTTTAACTTTATTCCGAAAACCGCCATGGAT
TTTTATCATGCAGTTCGCAGTGATGATCATGCAACCGTTCATCGTCTGATTCGTGATTTCTTTCTGCCGTATCTG
GCAATTCGTAATCGTAAAGCAGGTTATGCAGTGAGCATTGTTAAAGCCGGTGCAAAAATTGTTGGTCATGATGCA
GGTCCGGTTCGTCCGCCTCTGACCGATCTGACTCCGGCAGAAATGGAAGAACTGGCAGCACTGATTAAAAAGCTG
GGTCCGCAGTAA

MNPQELKNIVSSGLLSFPVTDFDANGDFNAASYAKRLEWLAPYGASALFAAGGTGEFFSLTGDEYSEV
IKTAVDTCRGSVPIIAGAGGPTRQAIEYAQEAERLGAHGILLLPHYLTEASQEGLIAHVKAVCKSVNFGVI
VYNRNVCRLTPDSLAKLAERCPNLIGFKDGVGDIELMTSITQKLGDRFSYLGGLPTAEVYAAPYKALGV
PVYSSAVFNFIPKTAMDFYHAVRSDDHATVHRLIRDFFLPYLAIRNRKAGYAVSIVKAGAKIVGHDAGPV
RPPLTDLTPAEMEELAALIKKLGPQ*

Ancestor N54

ATGGATGCACTGGAACTGAAAAACATTGTTAGTGATGGTCTGCTGAGCTTTCCGGTTACCGATTTTGATCAGAAT
GGTGATTTTAACGCAGCCAGCTATGCAAAACGTCTGGAATGGCTGGCACCGTATGGTGCAAGCGCACTGTTTGCA
GCCGGTGGCACCGGTGAATTTTTCAGCCTGACCGGTGATGAATATAGCGAAGTGATTAAAACCGCAGTTGATACC
TGTCGTGGTAGCGTTCCGATTATTGCCGGTGCCGGTGGTCCGACACGTCAGGCAATTGAACAGGCACAAGAGGCA
GAACGTCTGGGTGCACATGGTATTCTGCTGATGCCGCATTATCTGACCGAAGCAAGCCAAGAGGGTCTGATTGAA
CATGTTAAACAGGTTTGCAACAGCGTGAATTTTGGCGTGATTTTCTATAATCGTAGCGTGAGCCGTCTGAATCTG
GATAGCCTGCAGAAACTGACCGAAAGCTGTCCGAATCTGATTGGTTTTAAAGATAGCAGCGGTCAGATTGATATG
ATGACCGCAGTTACCCAGACACTGGGTGATCGTCTGAGCTATTTAGGTGGTCTGCCGACCGCAGAAGTTTTTGCA
GCACCGTATAAAGCACTGGGTTGTCCGGTTTATAGCAGCGCAGTTTTTAACTTTATTCCGAAAACCGCCATGGAA
TTCTATAATGCACTGCGCTCAGATGATTTTGCAACCACACATCGTCTGATTCGTGATTTCTTTCTGCCGCTGATT
AAAATCCGTAATCGCAAAAGCGGTTATGCCGTTAGCATGGTTAAAGCCGGTGCAAAAATTGTTGGTCATGATGCA
GGTCCGGTTCGTCCGCCTCTGAGCGATCTGACACCGGCAGATTATGAAGATCTGGCAGCACTGATTGCAACCCTG
GGTCCGCAGTAA

MDALELKNIVSDGLLSFPVTDFDQNGDFNAASYAKRLEWLAPYGASALFAAGGTGEFFSLTGDEYSEV
IKTAVDTCRGSVPIIAGAGGPTRQAIEQAQEAERLGAHGILLMPHYLTEASQEGLIEHVKQVCNSVNFG
VIFYNRSVSRLNLDSLQKLTESCPNLIGFKDSSGQIDMMTAVTQTLGDRLSYLGGLPTAEVFAAPYKAL
GCPVYSSAVFNFIPKTAMEFYNALRSDDFATTHRLIRDFFLPLIKIRNRKSGYAVSMVKAGAKIVGHDAG
PVRPPLSDLTPADYEDLAALIATLGPQ*

<u>Ancestor N55</u>

ATGAATCCGCAAGAACTGAAACAGACCGTTGGTAGCGGTCTGCTGAGCTTTCCGGTTACACATTTTGATGCAGAT
CTGGAATTTGATGAAGCAGCCTATCGTAAACATGTTGAATGGCTGAGCGGTTATGATGCAGCAGCACTGTTTGCA
GCCGGTGGCACCGGTGAATTTTTCAGCCTGACACCGGATGAAGTTCCGCAGGTTGTTCGTGCAGCAGTTGAAGCA
ACCGCAGGTCGTGTTCCGGTTATTGCCGGTGCAGGTTATGGCACCGCAATTGCCATTGAAATTGCACAGGCAGCA
GAAAAAGCGGGTGCAGATGGTATTCTGCTGCTGCCTCCGTATCTGACCGAAGCAAGCCAAGAGGGTCTGTATGCA
CATGTTAAAGCAGTTTGTGAAAGCACCAATCTGGGTGTGATTGTGTATAATCGTGATAATGCAGTTCTGACTCCG
GATACCATTGCACGTCTGGCAGAACGTTGTCCGAATCTGGTTGGTTTTAAAGATGGTGTGGGTGATATTGAACTG
ATGACCCGTATTACCGCAAAACTGGGTGATCGTCTGACCTATTTAGGTGGTCTGCCGACCGCAGAAACCTATGCA
CTGCCGTATCTGGCAATGGGTGTTACCACCTATAGCAGCGCAGTTTTTAACTTTGTGCCGGAACTGGCACTGGAT
TTTTATCGTGCAGTTCGTGCCGGTGATCATGCAACCGTTCATCGTCTGCTGCGTGATTTCTTTCTGCCGTTTGTT
GCAATTCGTAATCGCAAAAAGGGTTATGCCGTTAGCATTGTTAAAGCCGGTATGAAAGTTATTGGTCATGATGCA
GGTCCGGTTCGTCCTCCGCTGACCGATCTGACAGAAGAAGAACTGGCCGAACTGGCAGCACTGATTAAGAAAGCC
GGTGTTTAA

MNPQELKQTVGSGLLSFPVTHFDADLEFDEAAYRKHVEWLSGYDAAALFAAGGTGEFFSLTPDEVPQ
VVRAAVEATAGRVPVIAGAGYGTAIAIEIAQAAEKAGADGILLLPPYLTEASQEGLYAHVKAVCESTNLGV
IVYNRDNAVLTPDTIARLAERCPNLVGFKDGVGDIELMTRITAKLGDRLTYLGGLPTAETYALPYLAMGV
TTYSSAVFNFVPELALDFYRAVRAGDHATVHRLLRDFFLPFVAIRNRKKGYAVSIVKAGMKVIGHDAGP
VRPPLTDLTEEELAELAALIKKAGV*

The gene strings were purchased at GeneArt or Twist Bioscience after codon optimization for *E. coli*. The enzymes were used in a pET28a vector with kanamycin resistance and a His_6-tag. Ancestors N1, N2, N16, N24, N43, N44, N45 were also used in a pET28a vector containing the sequence of the maltose binding protein with a TEV protease cutting site to be used as a tag.

Table S1: Primers used in this work

Primer name	Sequence 5' -> 3'	Application
T7 promotor	TAATACGACTCACTATAGGG	Colony PCR and Sequencing
T7 terminator	CTAGTTATTGCTCAGCGGT	
pET backbone fwd	GCACCAGGCCGCTGCT	Amplification of the pET28a backbone
pET backbone rev	TAGAGACCTCCTCGAATTCG	
Insert fwd	AGCAGCGGCCTGGTGC	Amplification of synthetic genes
Insert rev	CGAATTCGAGGAGGTCTCTA	
MBP backbone fwd	CATATGGGCGCCCTGAAAATAAAGATTC	

MBP backbone rev	GAAGCTGAGTTGGCTGCTGCCAC	Amplification of the pET28a-MBP backbone
MBP insert fwd	CAGGGCGCCCATATGAGCAGCGGCCTGGTGCCG	Amplification of the insert for cloning into the MBP backbone
MBP insert rev	GCCAACTCAGCTTCCTTTCGGGC	
*Ab*KdgD G53 fwd	ACAGGTGAATTTTTTAGCCTGACCGGTGA	Introducing G53D or G53E mutation in *Ab*KdgD
*Ab*KdgD G53D rev	AAAATTCACCTGTATCACCGGCTGC	
*Ab*KdgD G53E rev	AAAATTCACCTGTTTCACCGGCTGC	
*Ab*KdgD P194E fwd	GGACGGCGAAAACGCCATCACGC	Introducing P194E mutation in *Ab*KdgD
*Ab*KdgD P194E rev	TTCGCCGTCCCAGGGGCCG	
*Ab*KdgD S212M fwd	GTGTTTAACTTTATTCCGAAAACCGCCATGGAA	Introducing S212M mutation in *Ab*KdgD
*Ab*KdgD S212M rev	GAATAAAGTTAAACACTGCGCTCATATAAACCGG	
*Ab*KdgD A214 fwd	CTATAAACCGGAGAACCCAGTGCTTTATACG	Introducing A214D or A214E mutation in *Ab*KdgD
*Ab*KdgD A214D rev	GGGTTCTCCGGTTTATAGCAGCGATGTG	
*Ab*KdgD A214E rev	GGGTTCTCCGGTTTATAGCAGCGAAGTG	
*Cn*KdaD F53T fwd	CGAACAGTTCGTGCTGAGCGACGACGAG	Introducing F53T mutation in *Cn*KdaD
*Cn*KdaD F53T rev	CACGAACTGTTCGGAGGTGTTGGCCAG	
*Cn*KdaD E200A fwd	CCTGGGACGGCGCCGAGGC	Introducing E200A mutation in *Cn*KdaD
*Cn*KdaD E200A rev	GCCGTCCCAGGGGCCGAC	
*Cn*KdaD E201N fwd	GGACGGCGAAAACGCCATCACGC	Introducing E201N mutation in *Cn*KdaD
*Cn*KdaD E201N rev	TTCGCCGTCCCAGGGGCCG	
*Cn*KdaD M217 fwd	GCGGGCTTCCCCGACGGCATC	Introducing M217T or M217S mutation in *Cn*KdaD
*Cn*KdaD M217T rev	GGGAAGCCCGCGCCGGTGGTGG	
*Cn*KdaD M217S rev	GGGGAAGCCCGCGCCGGTGCTGGC	

Table S2: List of the used wildtype plasmids of this work.

Plasmid	Source organism	Reference
pET28a *Cn*KdaD	*Cupriavidus necator N-1*	[7]
pET28a *Ab*KdgD	*Acinetobacter baylyi*	[14]
pET28a *Pp*KGSADH	*Pseudomonas putida*	[16]
pET28a Ancestor N1	Synthetic gene	This work
pEt28a Ancestor N1-MBP	Synthetic gene	This work

pET28a KdaD Ancestor N2	Synthetic gene	This work
pET28a KdaD Ancestor N2-MBP	Synthetic gene	This work
pET28a KdaD Ancestor N3	Synthetic gene	This work
pET28a KdaD Ancestor N4	Synthetic gene	This work
pET28a KdaD Ancestor N5	Synthetic gene	This work
pET28a KdaD Ancestor N9	Synthetic gene	This work
pET28a KdaD Ancestor N12	Synthetic gene	This work
pET28a KdaD Ancestor N16	Synthetic gene	This work
pET28a KdaD Ancestor N16-MBP	Synthetic gene	This work
pET28a KdaD Ancestor N24	Synthetic gene	This work
pET28a KdaD Ancestor N24-MBP	Synthetic gene	This work
pET28a KdaD Ancestor N43	Synthetic gene	This work
pET28a KdaD Ancestor N43-MBP	Synthetic gene	This work
pET28a KdaD Ancestor N44	Synthetic gene	This work
pET28a KdaD Ancestor N44-MBP	Synthetic gene	This work
pET28a KdaD Ancestor N45	Synthetic gene	This work
pET28a KdaD Ancestor N45-MBP	Synthetic gene	This work
pET28a KdaD Ancestor N46	Synthetic gene	This work
pET28a KdaD Ancestor N47	Synthetic gene	This work
pET28a KdaD Ancestor N54	Synthetic gene	This work
pET28a KdaD Ancestor N55	Synthetic gene	This work
pET28a *Cn*KdaD F53T	*Cupriavidus necator N-1*	This work
pET28a *Cn*KdaD F53T E200A	*Cupriavidus necator N-1*	This work
pET28a *Cn*KdaD F53T E201N	*Cupriavidus necator N-1*	This work
pET28a *Cn*KdaD F53T M217T	*Cupriavidus necator N-1*	This work
pET28a *Cn*KdaD F53T E200A M217T	*Cupriavidus necator N-1*	This work
pET28a *Ab*KdgD G53E A214E	*Acinetobacter baylyi*	This work
pET28a *Ab*KdgD P194E	*Acinetobacter baylyi*	This work
pET28a *Ab*KdgD P194E A214E	*Acinetobacter baylyi*	This work

Table S3: Molecular weight and extinction coefficient of the expressed and purified enzymes for the determination of the protein concentration via UV measurement. Parameters were calculated using the expasy ProtParam web server [19].

Enzyme	Molecular weight [kDa]	Extinction coefficient [$M^{-1}\cdot cm^{-1}$]
*Cn*KdaD	36.0	36440
*Ab*KdgD	34.8	21890
*Pp*KGSADH	57.7	28420
N1	35.5	17420
N1-MBP	78.8	85260
N2	36.0	17420
N2-MBP	79.3	85260
N3	36.3	29910
N4	36.2	29910
N5	36.1	28420
N9	36.3	29910
N12	35.4	16960
N16	34.7	8940
N16-MBP	78.0	76780
N24	34.8	8940
N24-MBP	78.1	76780
N43	34.6	7450
N43-MBP	77.8	75290

N44	34.8	14900
N44-MBP	78.1	82740
N45	34.7	13380
N45-MBP	77.9	91220
N46	34.7	26360
N47	34.7	24870
N54	34.8	21890
N55	34.5	23380
*Cn*KdaD F53T	36.0	36440
*Cn*KdaD F53T E200A	36.0	36440
*Cn*KdaD F53T E201N	36.0	36440
*Cn*KdaD F53T M217T	36.0	36440
*Cn*KdaD F53T E200A M217T	36.0	36440
*Ab*KdgD G53E A214E	34.8	21890
*Ab*KdgD P194E	34.8	21890
*Ab*KdgD P194E A214E	34.8	21890

Protein Purification – SDS Gels:

The purification of the enzymes was confirmed via SDS Page using 12 % SDS gels. The following figures show the results of the purification, confirming expression and purity of the enzymes.

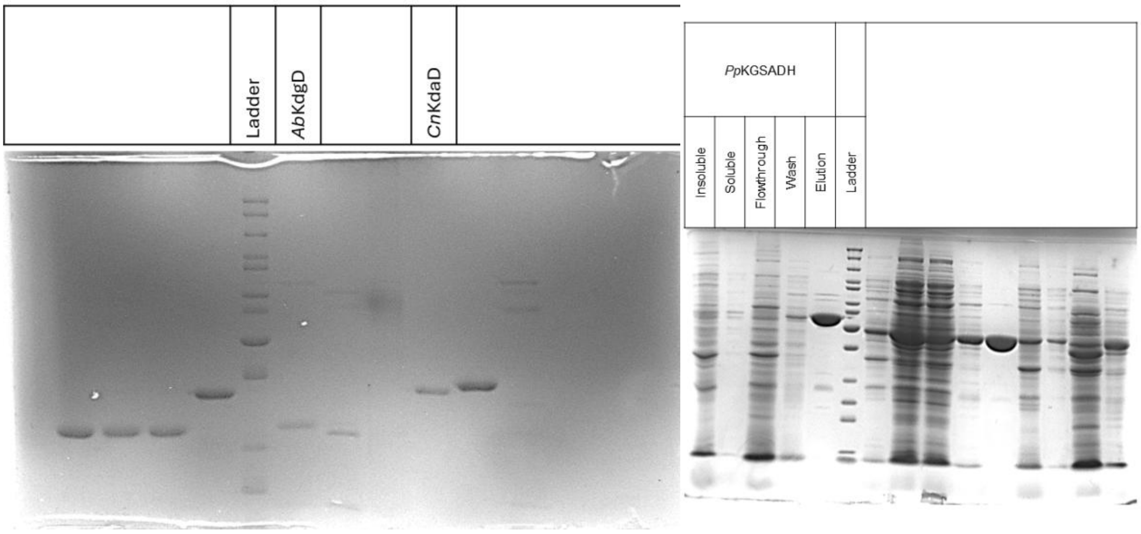

Figure S1: Uncropped SDS gel of the wildtypes used in this work. The dehydratases have an expected height of ~ 34 kDa for *Ab*KdgD and ~ 36 kDa for *Cn*KdaD. *Pp*KGSADH has an expected height of ~ 57 kDa.

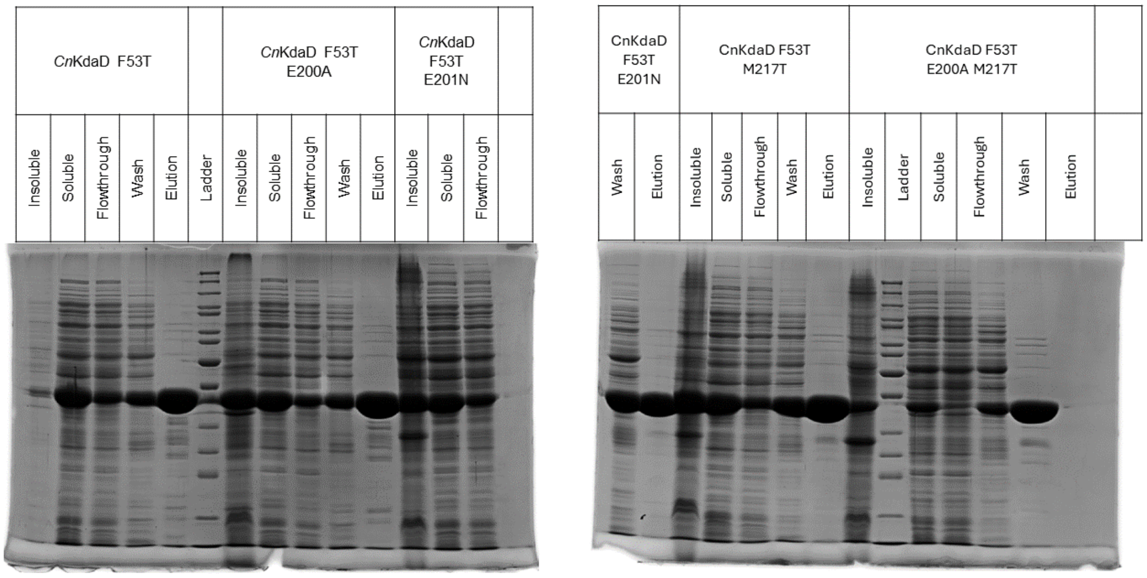

Figure S2: Full size SDS gel of the purification of the generated *Cn*KdaD variants (*Cn*KdaD F53T, *Cn*KdaD F53T E200A, *Cn*KdaD F53T E201N, *Cn*KdaD F53T M217T, and *Cn*KdaD F53T E200A M217T). The variants have expected heights of ~36 kDa.

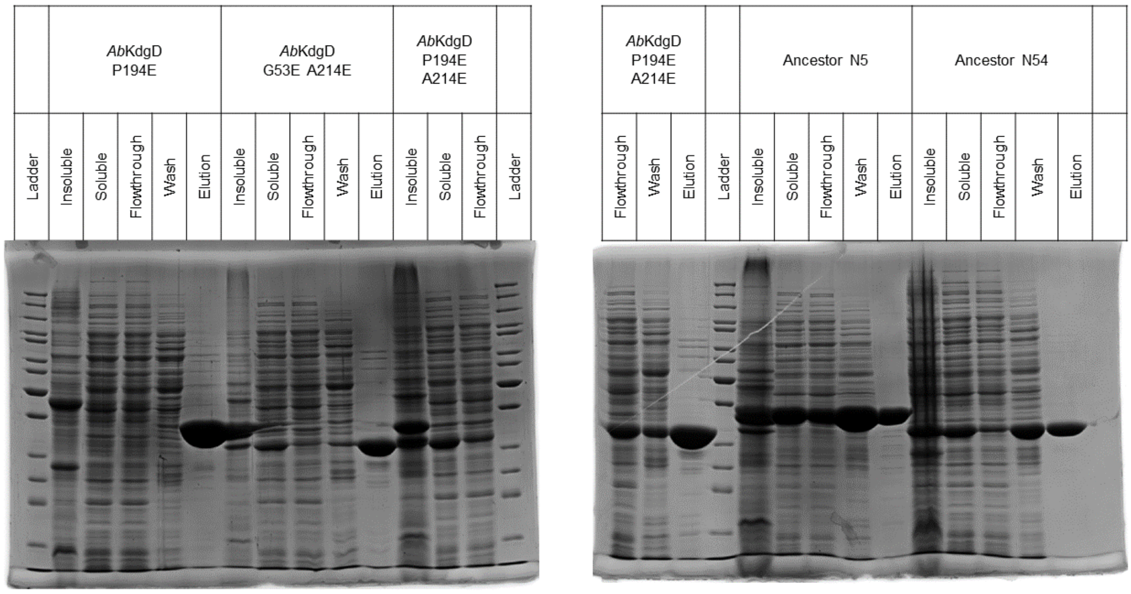

Figure S3: Full size SDS gel of the purification of the generated *Ab*KdgD variants (*Ab*KdgD P194E, *Ab*KdgD G53E A214E, *Ab*KdgD P194E A214E) and the ancestral enzymes N5 and N54. The expected height of the variants and N54 is ~ 34 kDa, while that of N5 is ~ 36 kDa.

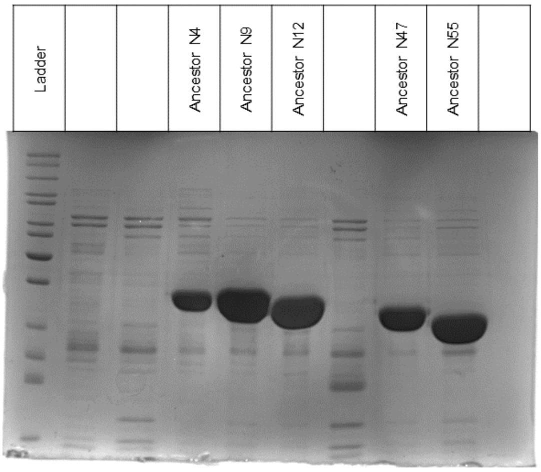

Figure S4: Uncropped SDS gel of the purified ancestors N4, N9, N12, N47 and N55. The Ancestors N3, N9, and N12 have an expected height of ~ 36 kDa, while the ancestors N47 and N55 have an expected height of ~ 34 kDa.

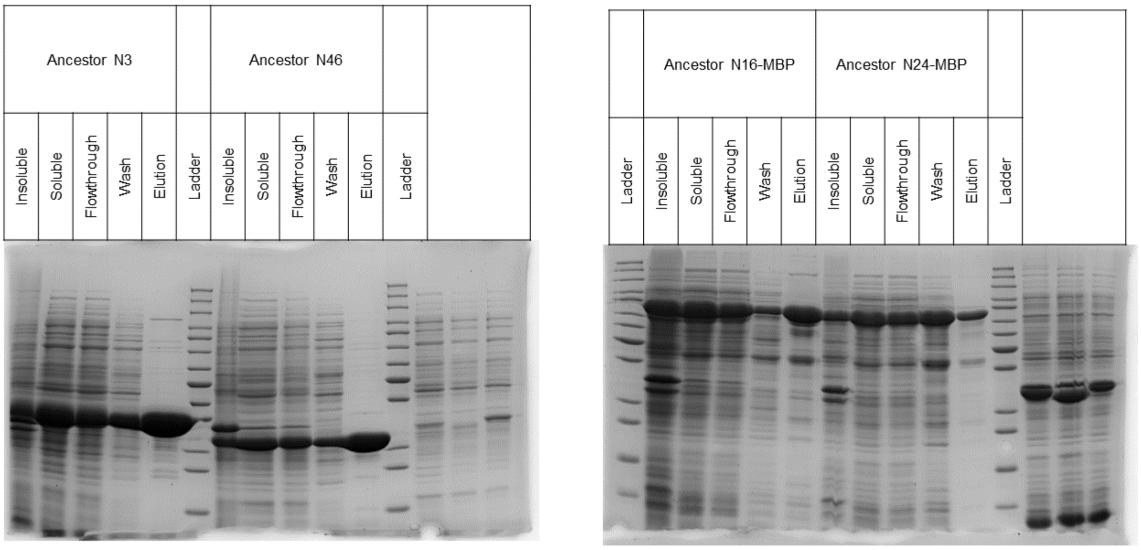

Figure S5: Full size SDS gel of the purification of the Ancestors N3, N46, N16-MBP and N24-MBP. The expected height of N3 is ~36 kDa, that of N46 is ~ 34kDa, and the tagged ancestors N16 and 24 have an expected height of ~ 80 kDa.

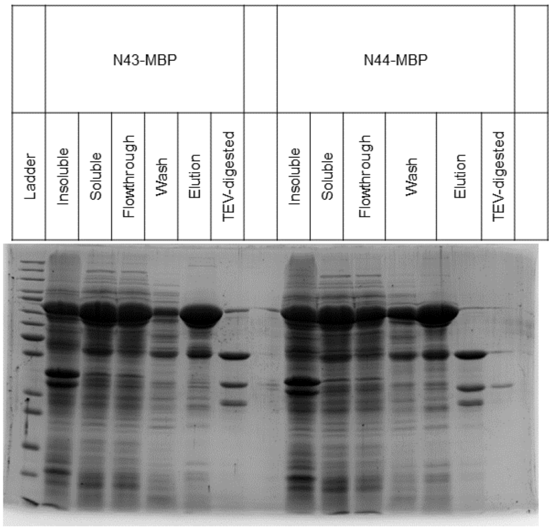

Figure S6: Full size SDS gel of the purification and maltose binding protein cleavage of the tagged ancestors N43-MBP and N44-MBP. The tagged enzymes have an expected height of ~ 80 kDa, the maltose binding protein has a height of ~ 55 kDa, and the untagged ancestors have expected heights of 30 - 40 kDa. In the sample after the digest, no band at 80 kDa is still visible, suggesting a successful digest.

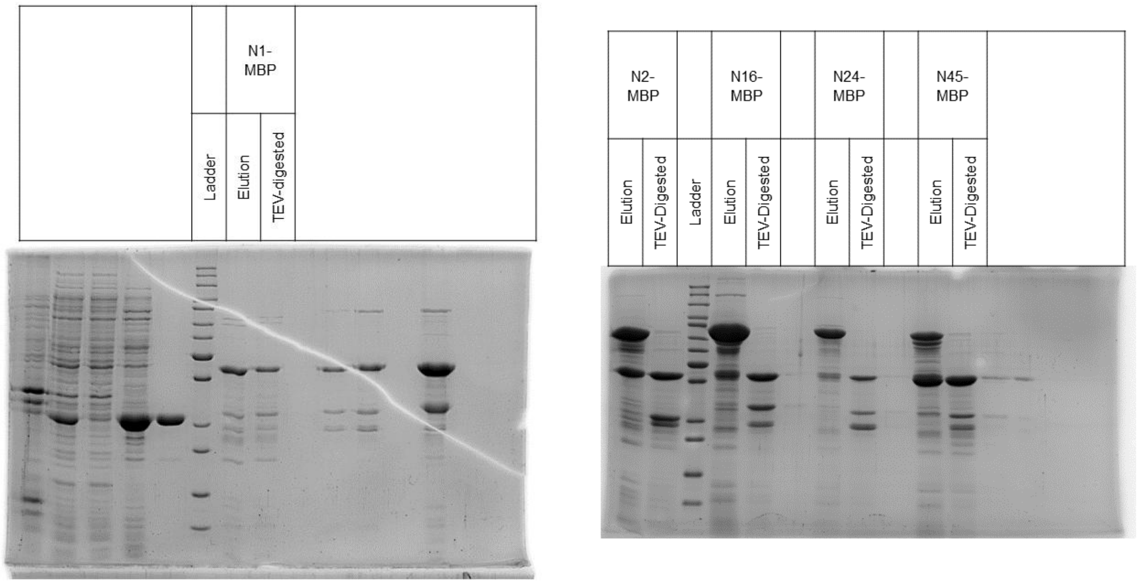

Figure S7: Uncropped SDS gel of the TEV digest of Ancestors N1, N2, N16, N24, and N45. The “Elution” sample shows the tagged enzyme after the initial his-tag purification, and the “TEV-digested” sample shows the enzyme and the maltose binding protein cleaved off after an incubation (4 °C, 16 h) with TEV protease. The tagged enzymes have an expected height of ~ 80 kDa, the maltose binding protein has a height of ~ 55 kDa, and the untagged ancestors have expected heights of 30 - 40 kDa. In the sample after the digest, no band at 80 kDa is still visible, suggesting a successful digest.

Table S4: Expression yields of the wildtype KdgDs and KdaDs as well as their ancestors and variants. Expression was carried out in *E. coli* BL21 in a 400 mL of autoinduction medium supplemented with 5 mM betaine and 1 M sorbitol at 30 °C for 24 h. After expression the enzymes were purified via affinity chromatography (His_6-tag purification) and size exclusion chromatography. The expression yield shows how much of each enzyme was obtained per liter of expression culture. In case of the tagged ancestors, enzyme mass and yield is shown before the cleavage of the maltose binding protein.

**Enzyme**	**Expression yield [mg enzyme/ L expression culture]**
*Cn*KdaD	29
*Ab*KdgD	12
N1-MBP	5
N2-MBP	9.5
N3	125
N4	24
N5	25
N9	44
N12	78
N16-MBP	13.5
N24-MBP	6.3
N43-MBP	10.3
N44-MBP	10.5
N45-MBP	8.8
N46	57
N47	27
N54	18
N55	62
*Cn*KdaD F53T	73
*Cn*KdaD F53T E200A	70
*Cn*KdaD F53T E201N	62
*Cn*KdaD F53T M217T	97
*Cn*KdaD F53T E200A M217T	49
*Ab*KdgD G53E A214E	32
*Ab*KdgD P194E	50
*Ab*KdgD P194E A214E	15

Kinetic measurement of the enzymes

The enzymes were tested for their activities towards three substrates, Kdg, Kda, and Kdx. The measured kinetics are listed in the following table. For cases where no kinetic was measurable due to too low activities, only the specific activity in U/mg is listed.

Table S5: Kinetic parameters of the used, active enzymes on 5-Keto-4-deoxy glucarate.

Enzyme	Km [mM]	Vmax [$U \cdot mg^{-1}$]	kcat [s^{-1}]	kcat·Km^{-1} [$s^{-1} \cdot mM^{-1}$]
*Cn*KdaD	-	-	-	-
*Ab*KdgD	0.12 ± 0.01	6.6101 ± 0.16255	6.9266 ± 0.17033	59.5119 ± 6.188
N3	-	-	-	-
N4	-	-	-	-
N5	-	-	-	-
N9	-	-	-	-
N12	-	-	-	-
N46	0.11 ± 0.02	0.2087 ± 0.01066	2.1820 ± 0.11151	19.9273 ± 4.1551
N47	0.66 ± 0.16	2.6955 ± 0.16843	2.9962 ± 0.18722	4.5280 ± 1.13748
N54	0.20 ± 0.04	9.2296 ± 0.24983	8.0071 ± 0.21836	40.2351 ± 0.2184
N55	0.02 ± 0.002	0.0356 ± 0.00076	0.0392 ± 0.00083	2.3072 ± 0.27582
*Cn*KdaD F53T	-	0.0003 ± 0.00003	-	-
*Cn*KdaD F53T E200A	-	0.0003 ± 0.00001	-	-
*Cn*KdaD F53T E201N	0.38 ± 0.07	0.0009 ± 0.00004	0.0001 ± 0.00004	0.0026 ± 0.00049
*Cn*KdaD F53T M217T	0.23 ± 0.07	0.0001 ± 0.00001	0.0001 ± 0.00001	0.0004 ± 0.00014
*Cn*KdaD F53T E200A M217T	0.77 ± 0.05	0.0005 ± 0.00001	0.0005 ± 0.00001	0.0007 ± 0.00004
*Ab*KdgD G53E A214E	0.18 ± 0.04	0.0008 ± 0.00004	0.0009 ± 0.00004	0.0050 ± 0.00113
*Ab*KdgD P194E	1.57 ± 0.12	0.5169 ± 0.01151	0.5784 ± 0.01288	0.3684 ± 0.02933
*Ab*KdgD P194E A214E	-	0.0005 ± 0.00009	-	-

Table S6: Kinetic parameters of the used, active enzymes on 2-Keto-3-deoxy-L-arabinonate.

Enzyme	Km [mM]	Vmax [$U \cdot mg^{-1}$]	kcat [s^{-1}]	kcat·Km^{-1} [$s^{-1} \cdot mM^{-1}$]
*Cn*KdaD	0.24 ± 0.03	14.0423 ± 0.45965	16.1591 ± 0.52884	68.5142 ± 8.86102
*Ab*KdgD	-	-	-	-
N3	0.49 ± 0.07	8.5001 ± 0.24479	9.3238 ± 0.26851	18.9008 ± 2.80435
N4	0.44 ± 0.15	1.2047 ± 0.09097	1.3979 ± 0.18556	3.1712 ± 1.13157
N5	0.55 ± 0.07	10.7029 ± 0.26582	11.6732 ± 0.28992	21.0707 ± 2.56419
N9	0.74 ± 0.09	14.5320 ± 0.47715	16.8902 ± 0.55462	22.7324 ± 2.85593
N12	1.66 ± 0.31	3.9582 ± 0.23813	4.4952 ± 0.27044	2.7032 ± 0.52660
N46	0.38 ± 0.05	0.0077 ± 0.00019	0.0080 ± 0.00019	0.0214 ± 0.00272
N47	-	-	-	-
N54	-	-	-	-
N55	2.87 ± 0.31	0.0005 ± 0.00002	0.0006 ± 0.00002	0.0002 ± 0.00003
*Cn*KdaD F53T	9.91 ± 0.86	0.2358 ± 0.01193	0.1455 ± 0.00736	0.0147 ± 0.00147
*Cn*KdaD F53T E200A	11.96 ± 1.28	0.8500 ± 0.48229	0.9820 ± 0.05572	0.0821 ± 0.00995
*Cn*KdaD F53T E201N	2.01 ± 0.46	0.0005 ± 0.00003	0.0006 ± 0.00004	0.0003 ± 0.00007
*Cn*KdaD F53T M217T	22.61 ± 12.29	0.0252 ± 0.00778	0.0252 ± 0.00899	0.0011 ± 0.00072

*Cn*KdaD F53T E200A M217T	25.56 ± 7.11	0.0039 ± 0.00070	0.0045 ± 0.00080	0.0002 ± 0.00006
*Ab*KdgD G53E A214E	0.48 ± 0.12	0.0017 ± 0.00010	0.0019 ± 0.00011	0.0040 ± 0.00102
*Ab*KdgD P194E	-	-	-	-
*Ab*KdgD P194E A214E	0.83 ± 0.23	0.0008 ± 0.00005	0.0009 ± 0.00006	0.0011 ± 0.00031

Table S7: Kinetic parameters of the used, active enzymes on 2-Keto-3-deoxy-D-xylonate.

**Enzyme**	**Km [mM]**	**Vmax [U·mg^{-1}]**	**kcat [s^{-1}]**	**kcat·Km^{-1} [s^{-1}·mM^{-1}]**
*Cn*KdaD	4.17 ± 1.68	0.2384 ± 0.04424	0.2502 ± 0.04642	0.0599 ± 0.02659
*Ab*KdgD	-	-	-	-
N3	-	0.4079 ± 0.03910	-	-
N4	7.05 ± 0.27	2.6127 ± 0.05239	3.0492 ± 0.06114	0.4329 ± 0.01847
N5	13.47 ± 4.03	2.0612 ± 0.39954	2.1597 ± 0.41895	0.1604 ± 0.05718
N9	7.30 ± 2.29	4.0304 ± 0.68302	4.7038 ± 0.79713	0.6448 ± 0.2304
N12	15.24 ± 2.94	0.8106 ± 0.08452	0.9138 ± 0.01315	0.0600 ± 0.01315
N46	-	-	-	-
N47	-	-	-	-
N54	-	-	-	-
N55	-	-	-	-
*Cn*KdaD F53T	9.59 ± 1.50	0.0131 ± 0.0012	0.0081 ± 0.00074	0.0008 ± 0.00015
*Cn*KdaD F53T E200A	15.59 ± 2.97	0.0744 ± 0.00747	0.0860 ± 0.00863	0.0055 ± 0.00119
*Cn*KdaD F53T E201N	-	0.0006 ± 0.00001	-	-
*Cn*KdaD F53T M217T	3.68 ± 1.07	0.0003 ± 0.00004	0.0004 ± 0.00004	0.0001 ± 0.00003
*Cn*KdaD F53T E200A M217T	3.12 ± 1.54	0.0006 ± 0.00012	0.0007 ± 0.00013	0.0002 ± 0.00012
*Ab*KdgD G53E A214E	0.18 ± 0.05	0.0006 ± 0.00003	0.0007 ± 0.00004	0.0039 ± 0.0011
*Ab*KdgD P194E	-	-	-	-
*Ab*KdgD P194E A214E	1.88 ± 0.64	0.0015 ± 0.00015	0.0017 ± 0.00017	0.0009 ± 0.00032

Michaelis-Menten graphs for the measured kinetics:

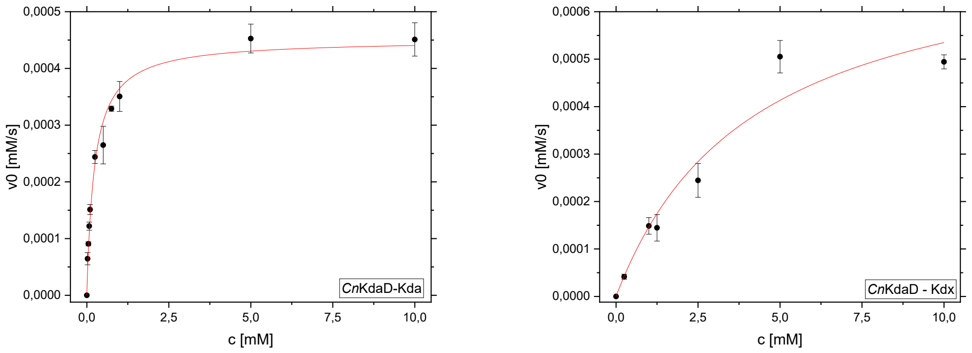

Figure S8: Michaelis-Menten kinetics of *Cn*KdaD with 2-Keto-3-deoxy-L-arabinonate (left) and 2-Keto-3-deoxy-D-xylonate (right). Measurements were carried out in 50 mM HEPES buffer (pH 7.5) with 5 mM MgCl2, 2.5 mM NAD, 0.01 – 10 mM of substrate, 170 nM *Pp*KGSADH and 0.001 - 0.01 mg/mL *Cn*KdaD.

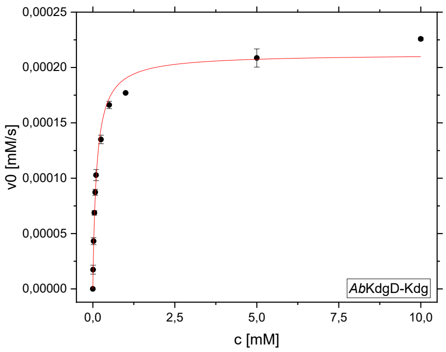

Figure S9: Michaelis-Menten kinetics of *Ab*KdgD with 5-Keto-4-deoxy glucarate. Measurements were carried out in 50 mM HEPES buffer (pH 7.5) with 5 mM MgCl2, 2.5 mM NAD, 0.01 – 10 mM of substrate, 170 nM *Pp*KGSADH and 0.001 mg/mL *Ab*KdgD.

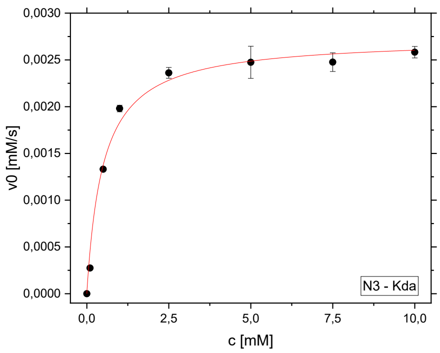

Figure S10: Michaelis-Menten kinetics of KdaD Ancestor N3 with 2-Keto-3-L-arabinonate. Measurements were carried out in 50 mM HEPES buffer (pH 7.5) with 5 mM MgCl2, 2.5 mM NAD, 0.01 – 10 mM of substrate, 170 nM *Pp*KGSADH and 0.01 mg/mL enzyme.

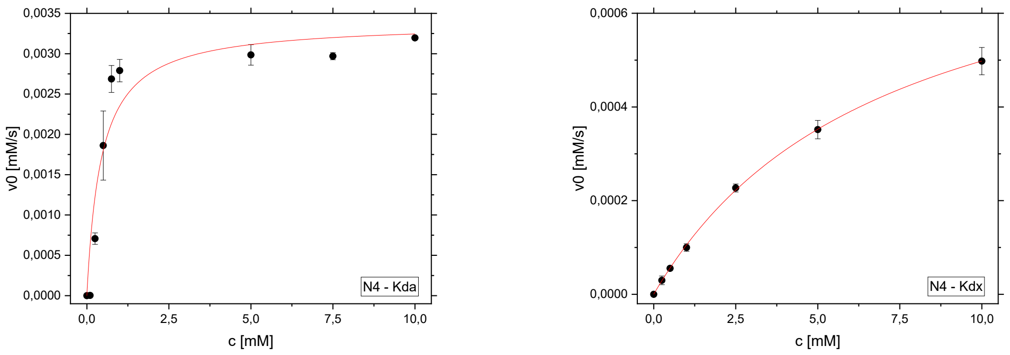

Figure S11: Michaelis-Menten kinetics of KdaD Ancestor N4 with 2-Keto-3-deoxy-L-arabinonate (left) and 2-Keto-3-deoxy-D-xylonate (right). Measurements were carried out in 50 mM HEPES buffer (pH 7.5) with 5 mM MgCl2, 2.5 mM NAD, 0.01 – 10 mM of substrate, 170 nM *Pp*KGSADH and 0.01 - 1 mg/mL enzyme.

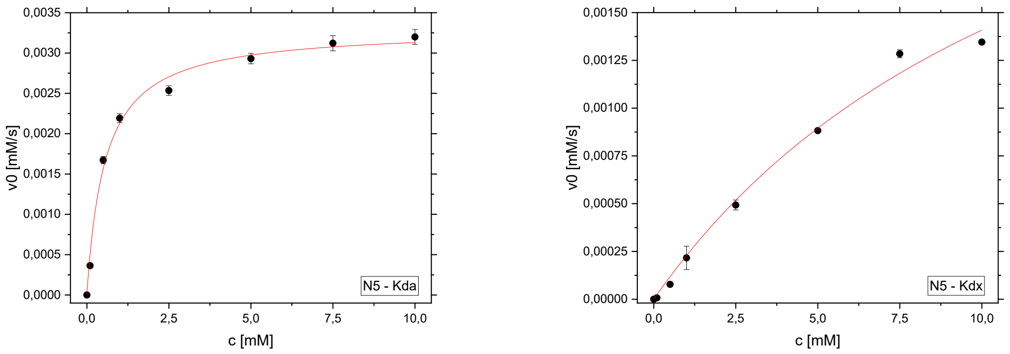

Figure S12: Michaelis-Menten kinetics of KdaD Ancestor N5 with 2-Keto-3-deoxy-L-arabinonate (left) and 2-Keto-3-deoxy-D-xylonate (right). Measurements were carried out in 50 mM HEPES buffer (pH 7.5) with 5 mM MgCl2, 2.5 mM NAD, 0.01 – 10 mM of substrate, 170 nM *Pp*KGSADH and 0.01 - 1 mg/mL enzyme.

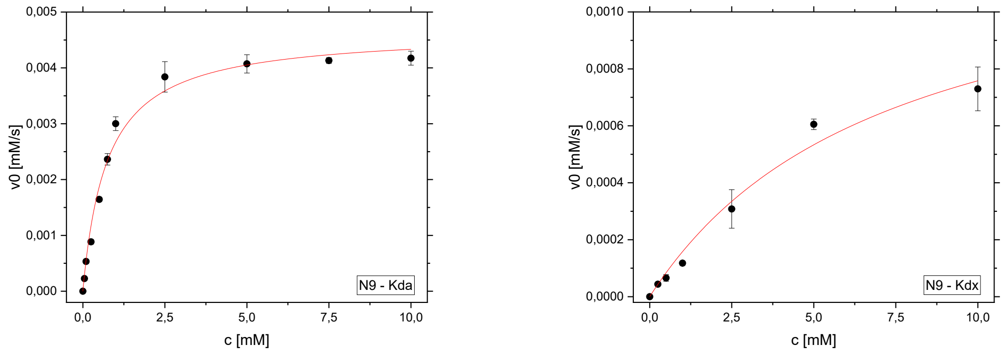

Figure S13: Michaelis-Menten kinetics of KdaD Ancestor N9 with 2-Keto-3-deoxy-L-arabinonate (left) and 2-Keto-3-deoxy-D-xylonate (right). Measurements were carried out in 50 mM HEPES buffer (pH 7.5) with 5 mM MgCl2, 2.5 mM NAD, 0.01 – 10 mM of substrate, 170 nM *Pp*KGSADH and 0.01 - 1 mg/mL enzyme.

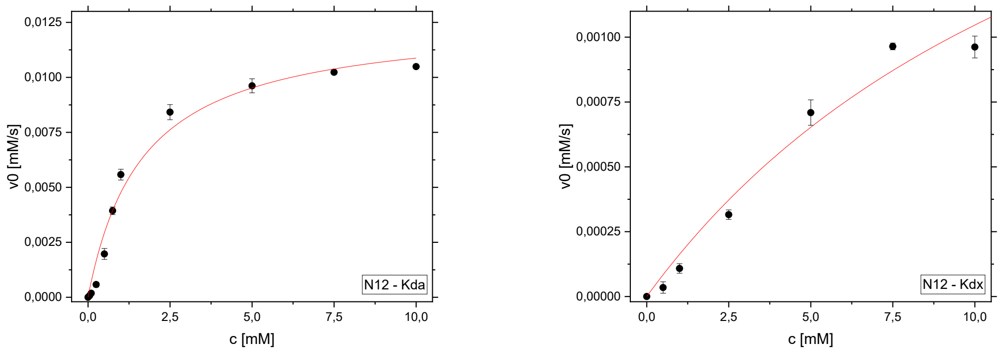

Figure S14: Michaelis-Menten kinetics of KdaD Ancestor N12 with 2-Keto-3-deoxy-L-arabinonate (left) and 2-Keto-3-deoxy-D-xylonate (right). Measurements were carried out in 50 mM HEPES buffer (pH 7.5) with 5 mM MgCl2, 2.5 mM NAD, 0.01 – 10 mM of substrate, 170 nM *Pp*KGSADH and 0.01 - 1 mg/mL enzyme.

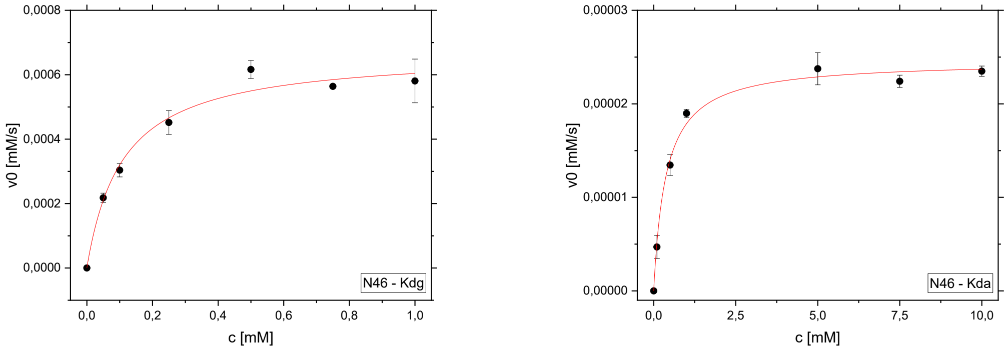

Figure S15: Michaelis-Menten kinetics of KdgD Ancestor N46 with 5-Keto-4-deoxy glucarate (left) and 2-Keto-3-deoxy-L-arabinonate (right). Measurements were carried out in 50 mM HEPES buffer (pH 7.5) with 5 mM MgCl2, 2.5 mM NAD, 0.01 – 10 mM of substrate, 170 nM *Pp*KGSADH and 0.01 - 1 mg/mL enzyme.

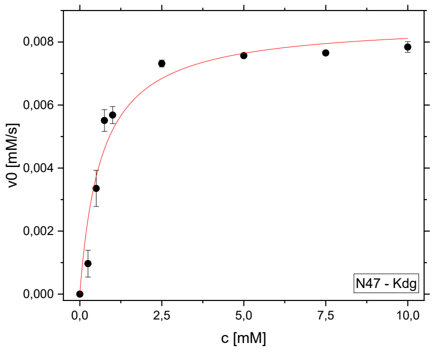

Figure S16: Michaelis-Menten kinetics of KdgD Ancestor N47 with 5-Keto-4-deoxy glucarate. Measurements were carried out in 50 mM HEPES buffer (pH 7.5) with 5 mM MgCl2, 2.5 mM NAD, 0.01 – 10 mM of substrate, 170 nM *Pp*KGSADH and 0.01 mg/mL enzyme.

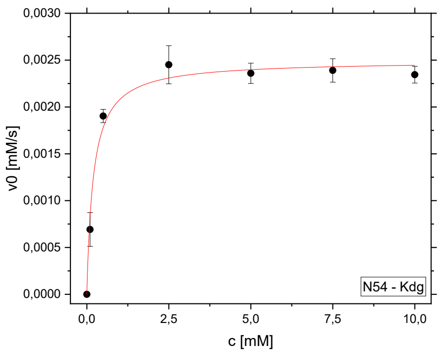

Figure S17: Michaelis-Menten kinetics of KdgD Ancestor N55 with 5-Keto-4-deoxy glucarate. Measurements were carried out in 50 mM HEPES buffer (pH 7.5) with 5 mM MgCl2, 2.5 mM NAD, 0.01 – 10 mM of substrate, 170 nM *Pp*KGSADH and 0.01 mg/mL enzyme.

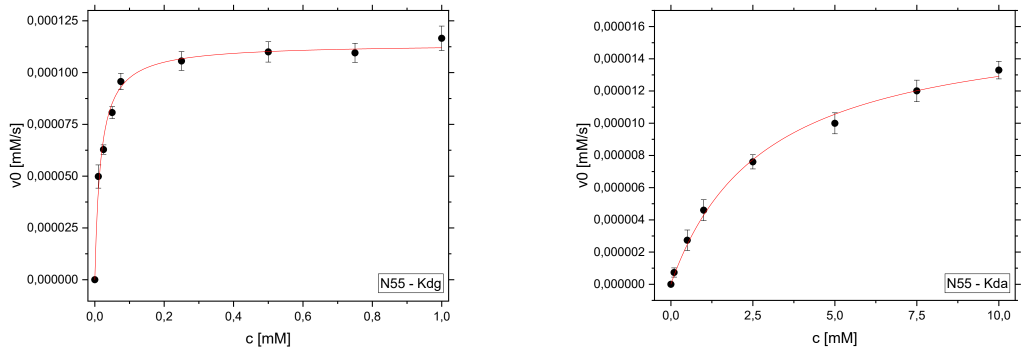

Figure S18: Michaelis-Menten kinetics of KdgD Ancestor N55 with 5-Keto-4-deoxy glucarate (left) and 2-Keto-3-deoxy-L-arabinonate (right). Measurements were carried out in 50 mM HEPES buffer (pH 7.5) with 5 mM MgCl2, 2.5 mM NAD, 0.01 – 10 mM of substrate, 170 nM *Pp*KGSADH and 0.01 - 1 mg/mL enzyme.

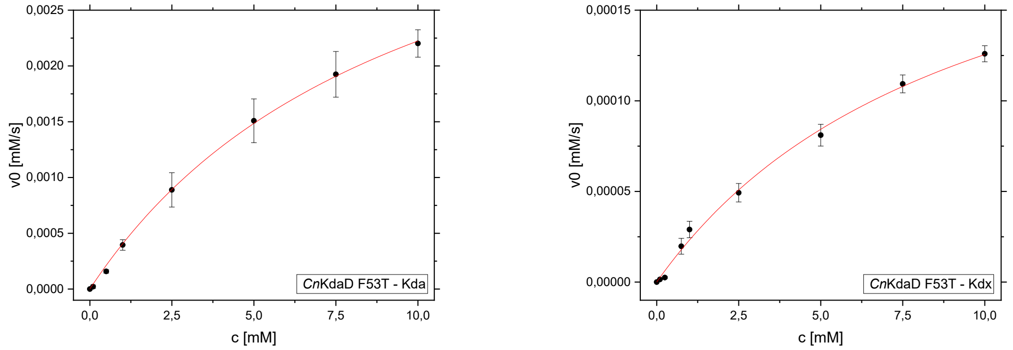

Figure S19: Michaelis-Menten kinetics of *Cn*KdaD F53T with 2-Keto-3-deoxy-L-arabinonate (left) and 2-Keto-3-deoxy-D-xylonate (right). Measurements were carried out in 50 mM HEPES buffer (pH 7.5) with 5 mM MgCl2, 2.5 mM NAD, 0.01 – 10 mM of substrate, 170 nM *Pp*KGSADH and 0.1 - 1 mg/mL enzyme.

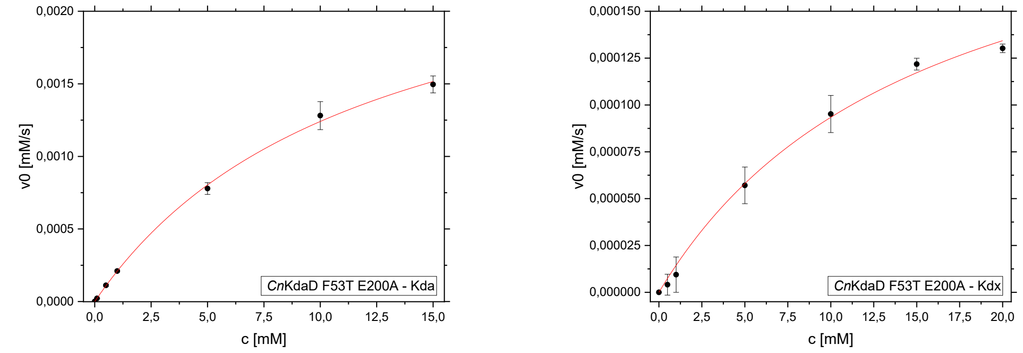

Figure S20: Michaelis-Menten kinetics of *Cn*KdaD F53T E200A with 2-Keto-3-deoxy-L-arabinonate (left) and 2-Keto-3-deoxy-D-xylonate (right). Measurements were carried out in 50 mM HEPES buffer (pH 7.5) with 5 mM MgCl2, 2.5 mM NAD, 0.01 – 10 mM of substrate, 170 nM *Pp*KGSADH and 0.1 - 1 mg/mL enzyme.

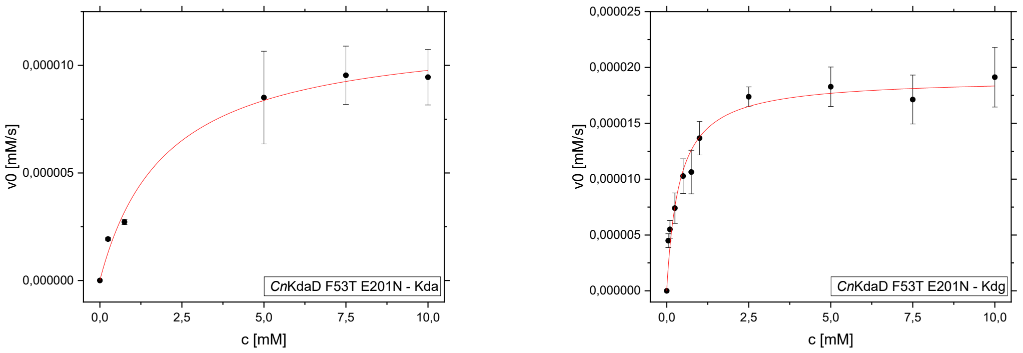

Figure S21: Michaelis-Menten kinetics of *Cn*KdaD F53T E201N with 2-Keto-3-deoxy-L-arabinonate (left) and 5-Keto-4-deoxy glucarate (right). Measurements were carried out in 50 mM HEPES buffer (pH 7.5) with 5 mM MgCl2, 2.5 mM NAD, 0.01 – 10 mM of substrate, 170 nM *Pp*KGSADH and 0.1 - 1 mg/mL enzyme.

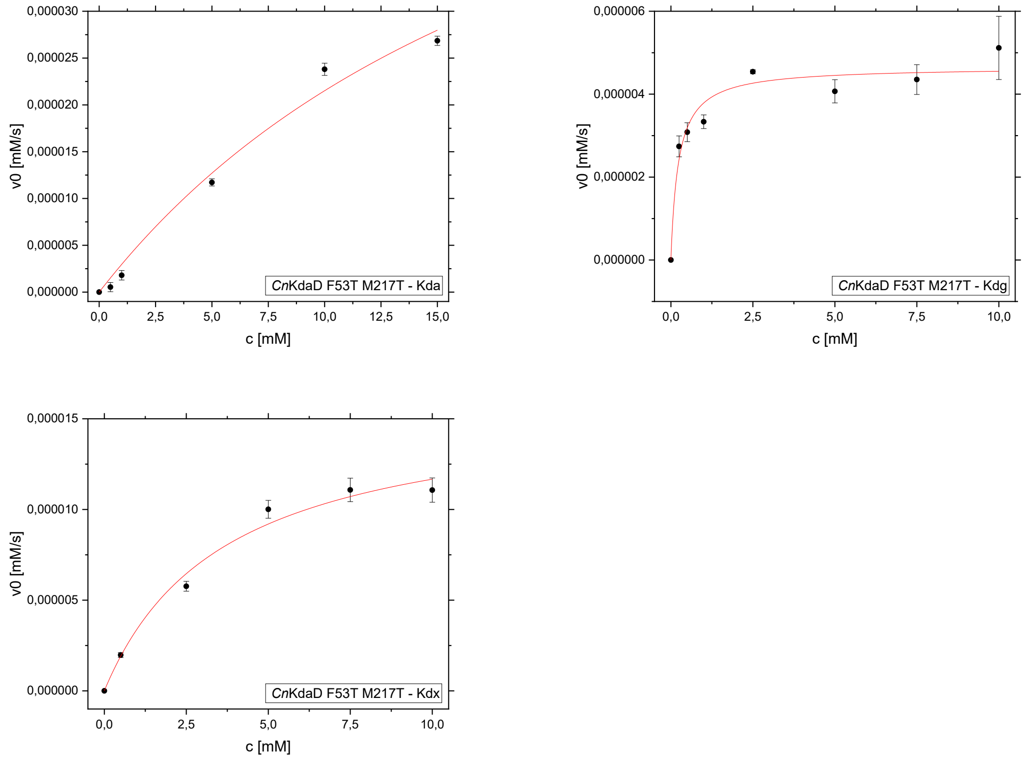

Figure S22: Michaelis-Menten kinetics of *Cn*KdaD F53T M217T with 2-Keto-3-deoxy-L-arabinonate (top left), 5-Keto-4-deoxy glucarate (top right) and 2-Keto-3-deoxy-D-xylonate (bottom left). Measurements were carried out in 50 mM HEPES buffer (pH 7.5) with 5 mM MgCl2, 2.5 mM NAD, 0.01 – 10 mM of substrate, 170 nM *Pp*KGSADH and 0.1 - 1 mg/mL enzyme.

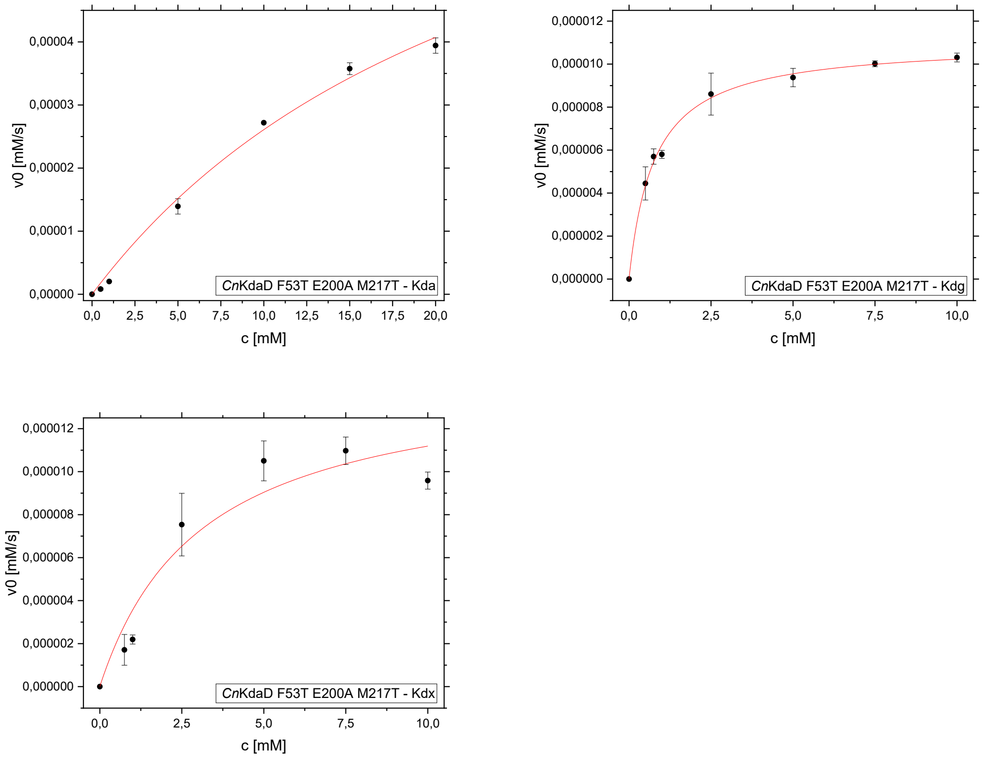

Figure S23: Michaelis-Menten kinetics of *Cn*KdaD F53T E200A M217T with 2-Keto-3-deoxy-L-arabinonate (top left), 5-Keto-4-deoxy glucarate (top right) and 2-Keto-3-deoxy-D-xylonate (bottom left).

Measurements were carried out in 50 mM HEPES buffer (pH 7.5) with 5 mM MgCl2, 2.5 mM NAD, 0.01 – 10 mM of substrate, 170 nM *Pp*KGSADH and 0.1 - 1 mg/mL enzyme.

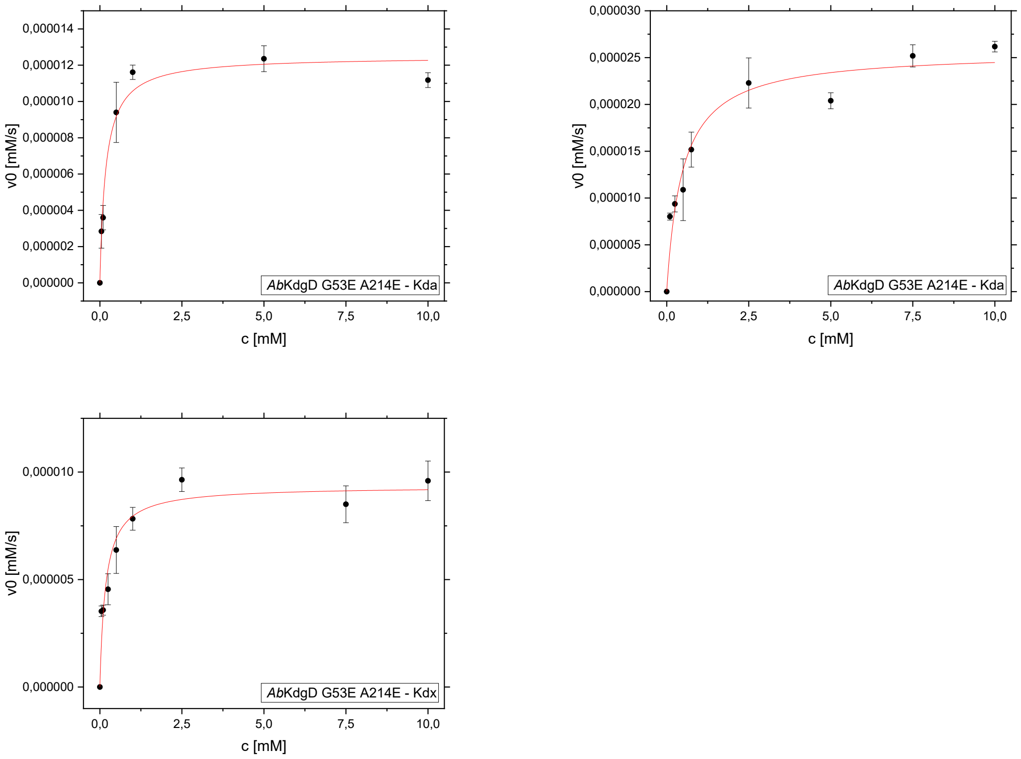

Figure S24: Michaelis-Menten kinetics of *Ab*KdgD G53E A214E with 2-Keto-3-deoxy-L-arabinonate (top left), 5-Keto-4-deoxy glucarate (top right) and 2-Keto-3-deoxy-D-xylonate (bottom left). Measurements were carried out in 50 mM HEPES buffer (pH 7.5) with 5 mM MgCl2, 2.5 mM NAD, 0.01 – 10 mM of substrate, 170 nM *Pp*KGSADH and 0.1 - 1 mg/mL enzyme.

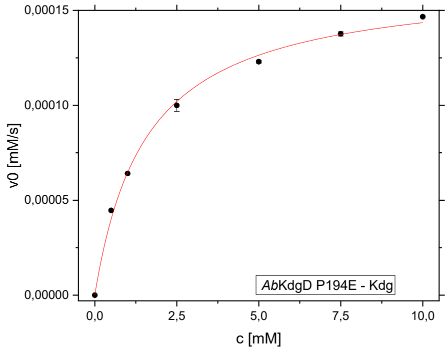

Figure S25: Michaelis-Menten kinetics of *Ab*KdgD P194E with 5-Keto-4-deoxy glucarate. Measurements were carried out in 50 mM HEPES buffer (pH 7.5) with 5 mM MgCl2, 2.5 mM NAD, 0.01 – 10 mM of substrate, 170 nM *Pp*KGSADH and 0.1 mg/mL enzyme.

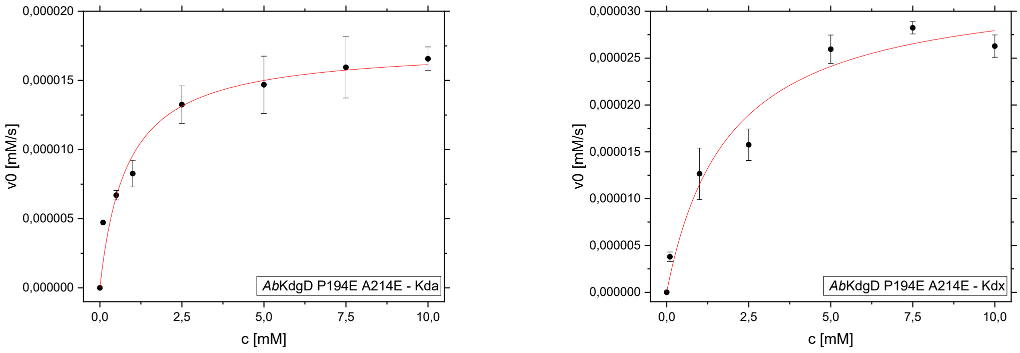

Figure S26: Michaelis-Menten kinetics of *Ab*KdgD P194E A214E with 2-Keto-3-deoxy-L-arabinonate (left) and 2-Keto-3-deoxy-D-xylonate (right). Measurements were carried out in 50 mM HEPES buffer (pH 7.5) with 5 mM MgCl2, 2.5 mM NAD, 0.01 – 10 mM of substrate, 170 nM *Pp*KGSADH and 0.1 - 1 mg/mL enzyme.

GFN2-xTB cluster model analysis

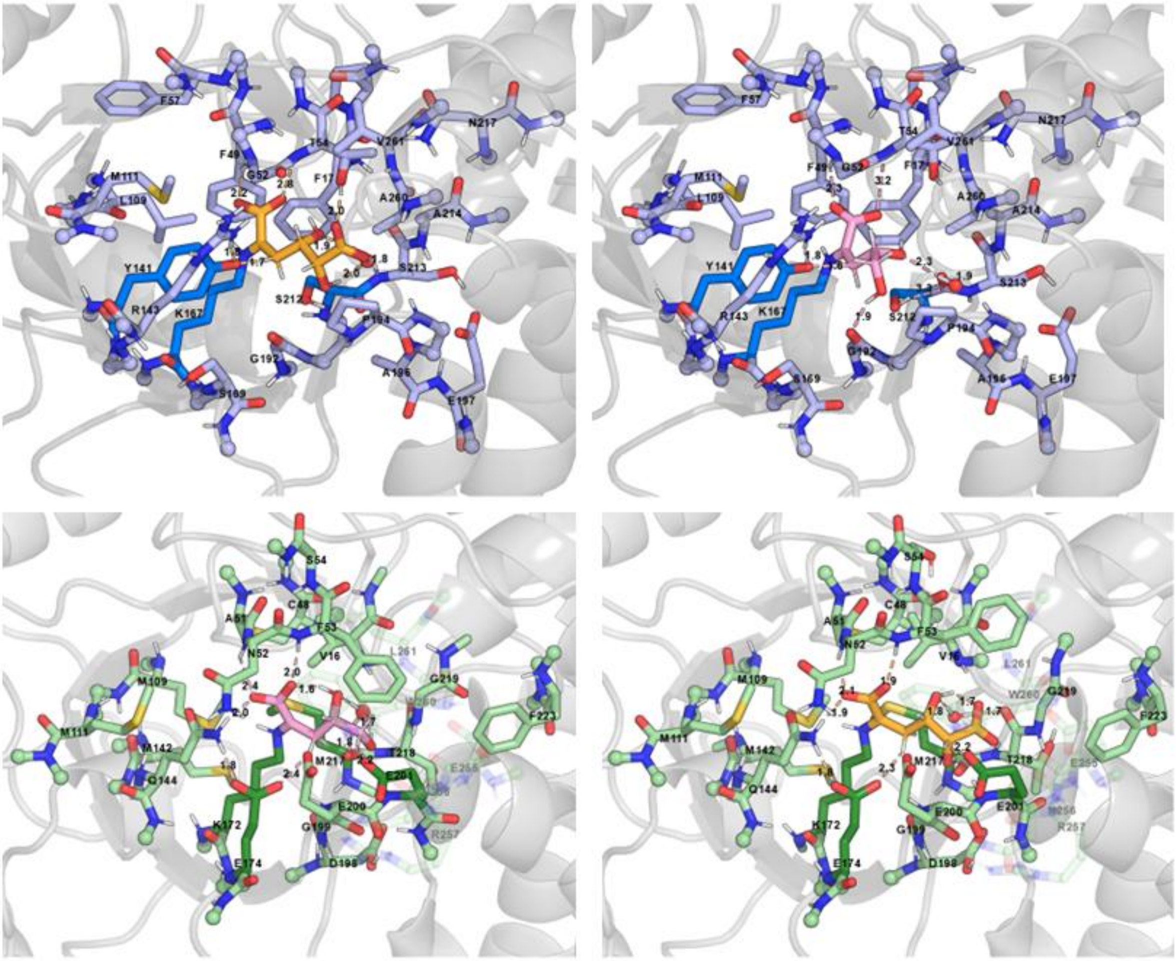

Figure S27: Results of the GFN2-xTB cluster model analysis of *Ab*KdgD (top) and *Cn*KdaD (bottom) with their native substrate (left) or the non-native substrate (right) and the catalytic water. The residues, which were considered for the calculations, are shown as sticks, a darker color was used to mark the catalytic residues. The substrates are highlighted in orange for Kdg, and pink for Kda. Atoms which were fixed during the calculations are shown as spheres with the exception of the water molecule, which was flexible. Interactions between the substrate and the amino acid residues are indicated with dashes. The distance of each interaction is annotated in angstroms. In case of the structures of *Cn*KdaD, some residues are between the substrate and the reader, these residues were made transparent in order to not obstruct the vision of the active site.

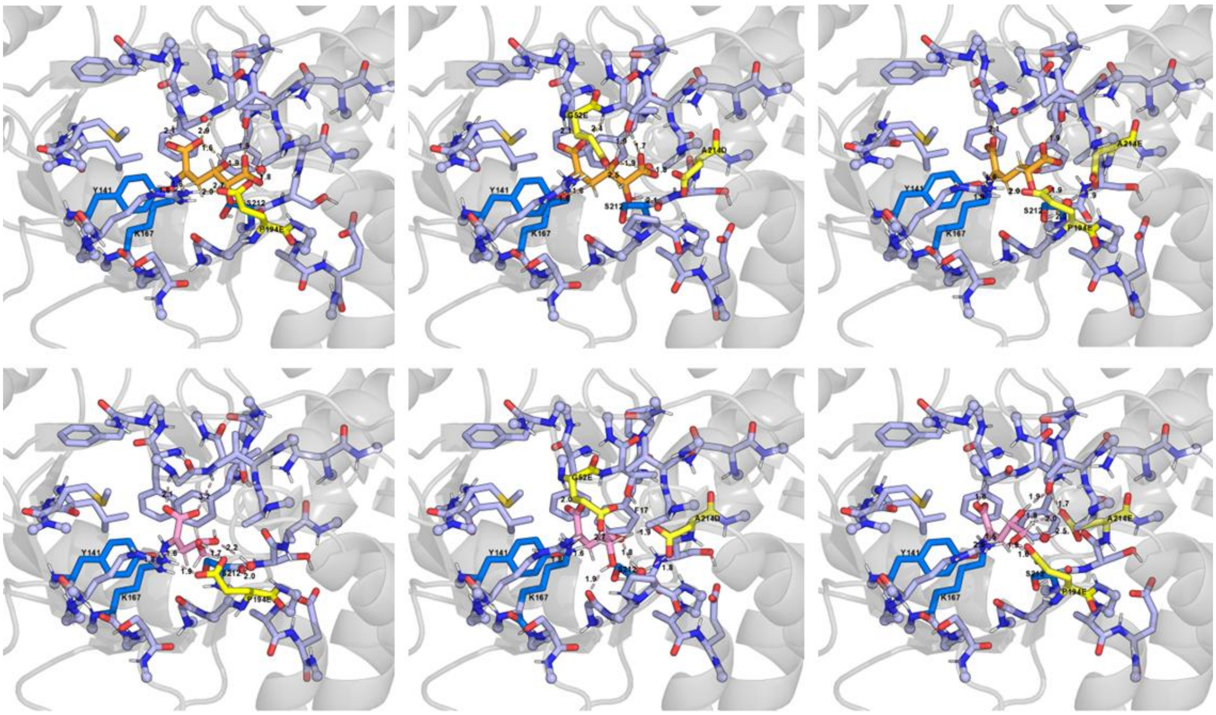

Figure S28: Results of the GFN2-xTB cluster model analysis of the AbKdgD variants P194E, G53E A214, and P194E A214 with the native substrate, Kdg (orange, top) and the non-native substrate, Kda (pink, bottom). The residues, which were considered for the calculations, are shown as sticks, a darker color was used to mark the catalytic residues. Mutated residues are highlighted in yellow. Atoms which were fixed during the calculations are shown as spheres with the exception of the water molecule, which was flexible. Interactions between the substrate and the amino acid residues are indicated with dashes. The distance of each interaction is annotated in angstroms.

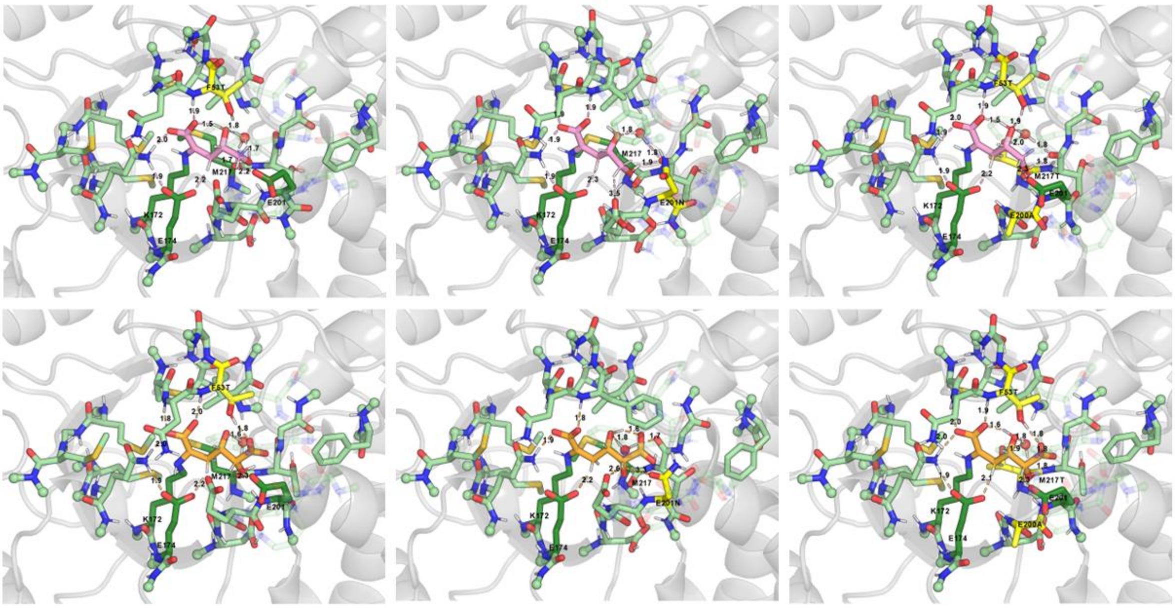

Figure S29: Results of the GFN2-xTB cluster model analysis of the CnKdaD variants F53T, E201N, and F53T E200A M217T with the native substrate, Kda (pink, top) and the non-native substrate, Kdg (orange, bottom). The residues, which were considered for the calculations, are shown as sticks, a darker color was used to mark the catalytic residues. Mutated residues are highlighted in yellow. Atoms which were fixed during the calculations are shown as spheres with the exception of the water molecule, which was flexible. Interactions between the substrate and the amino acid residues are indicated with dashes. The distance of each interaction is annotated in angstroms.

Generation of a phylogenetic tree:

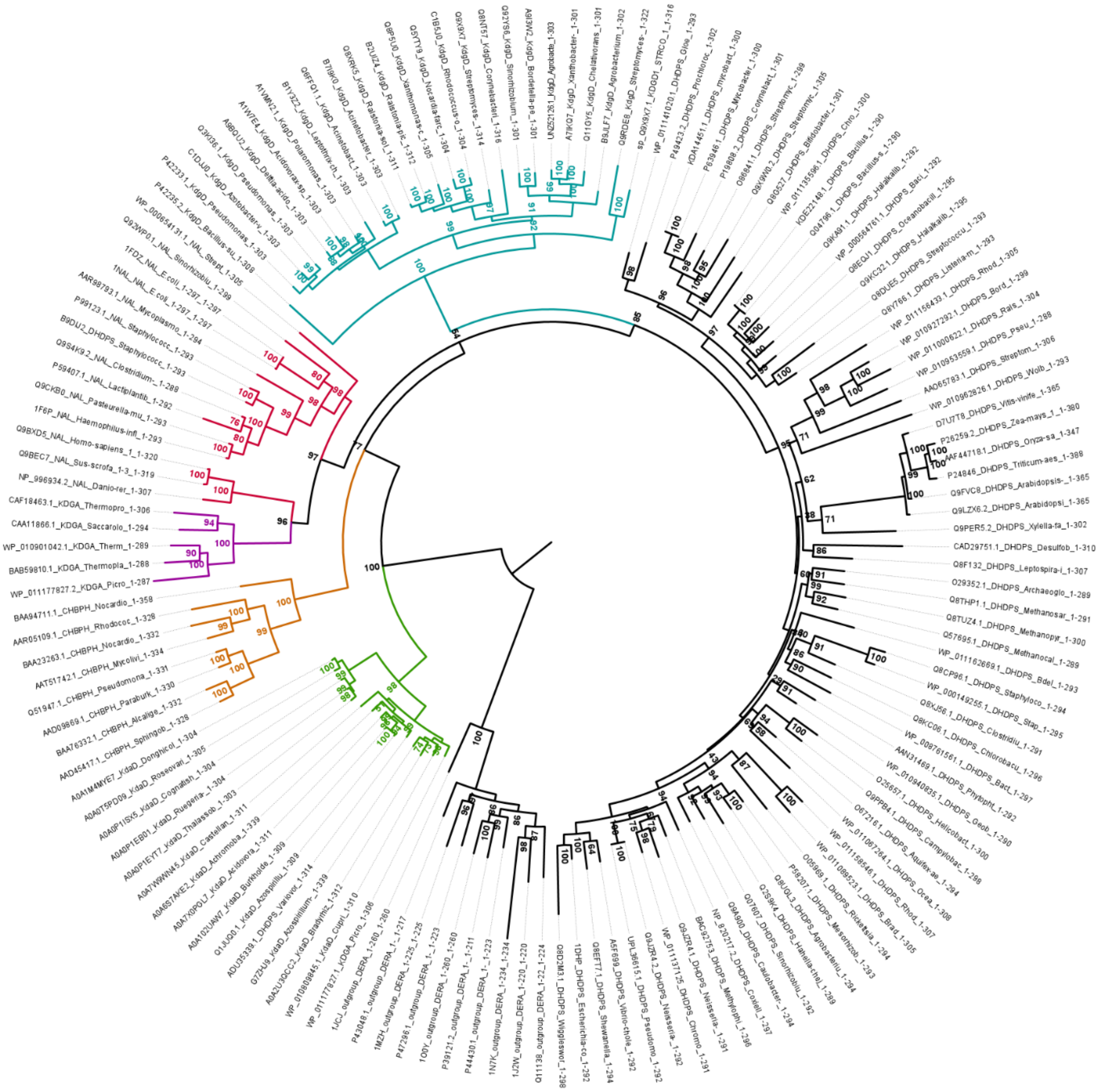

Figure S30: Phylogenetic tree of the DHDPS superfamily that was used for the ancestral sequence reconstruction. Tree branches are labeled with the uniprot accession number and the subfamily of the enzyme. Tree nodes are labelled with bootstrap values.

Multiple Sequence Alignments

```
CnKdaD    MTRT-PSPTVYRGVFPVAPTVFDAQGGLDLDGQRRCIDFMIDAGSHGICILANFSEQFVLSDDERNVLMK
AbKdgD    MDALELKNIVSDGLLSFPVTDFDQNGDFNAASYAKRLEWLAPYGASALFAAGGTGEFFSLTGDEYSDVIK


CnKdaD    TVLEHVDGRVPVIVTTTHFSSRLCAERSRAAQDAGAAMVMVMPPYHGATIRVPERSIYEFFATVSDAIDI
AbKdgD    TAVDACKGSVPIIAGAGG-PTRQAILQAQEAERLGAHGILLMPHYLTE---ASQEGLVEHVKQVCNAVNF


CnKdaD    PIMIQDAPVSGTTLSAPFLARMAREIGNVSYFKIEVPQAAAKLRELIELGGDAIVGPWDGEE-AITLMAD
AbKdgD    GVIFYNRS--VSKLNVDSLQQLVESCPNLIGFKDSS-GQIDMMTEVVQTLGDRLSYLGGLPTAEIFAAPY


CnKdaD    LEAGATGAMTGAG--FPDGIRQILDAWQAGDAELAAQ-RYQQWLPLINYENRQG--WLATSKVLMQAGGV
AbKdgD    KALGSPVYSSAVFNFIPKTAMEFYNALRNDDFATTQRLIRDFFLPLIKIRNRKSGYAVSMVKAGAKIVGH


CnKdaD    IASDAVRHPLQPMHPATREGLLKIARRLDPLVLRWAR
AbKdgD    DA-GPVRPPLSDLTPQDYEDLAALIATLGPQ------
```

Figure S31: Alignment of *Cn*KdaD and *Ab*KdgD. Highlighted in blue and green are the active site residues, while the yellow shows the mutation sites for the rational design.

```
KdaD_Cupriavidus-necator   -MTRTPSPT-VYRGVFPVAPTVFDAQGGLDLDGQRRCIDFMIDAGSHGICILANFSEQFVLSDDERNVLMKTVLEHVDGRVPVIVTTTHFSSRLCAERSR
IQtree-N2                  -MLGLPSSTKRYRGVFPVAPTPFDEDGELDLEGLKRVVDFMIDQGVDGICILANFSEQFVLSDDEREVLTKTVLEHVAGRVPVIVTTSHYSTRVAVERAR
IQtree-N3                  --MTRPSSTPRYRGVFPVAPTIFDEDGELDLEGQKRCVDFMIDAGSDGLCILANFSEQFVLSDDEREVLTKTVLEHVAGRVPVIVTTTHYSTRVCAERSR
IQtree-N4                  -MLGLPSSTPPYRGVFPVAPTIFDEDGELDLEGQKRCIDFMIDAGSNGLCILANFSEQFVLSDDEREVLTKTVLEHVAGRVPVIVTTTHYSSRVCAERSR
IQtree-N5                  --MTRPSSTPPYRGVFPVAPTIFDEQGELDLEGQKRCIDFMIDAGSHGLCILANFSEQFVLSDDEREVLMKTVLEHVAGRVPVIVTTTHFSSRVCAERSR
IQtree-N6                  RPRRRPSSTRPYRGVFPVAPTIFDEQGELDLEGQKRCIDFMIDAGSHGLCILANFSEQFVLTDDEREVLMKTVLEHVAGRVPVIVTTTHFSSRVCAERSR
IQtree-N7                  -MPSTTSSTPRYRGVFPVVPTIFTEDGELDLEGQKRCVDFMIDAGSDGLCILANFSEQFVLSDDEREVLTKTILEHVAGRVPVIVTTTHYSTRVCAERSR
IQtree-N8                  -MPSTTSSTPRYRGIFPVVPTTFTEDGELDLESQKRCVDFMIDAGSDGLCILANFSEQFVLSDDEREVLTRTILEHVAGRVPVIVTTTHYSTRVCAERSR
IQtree-N9                  -MPSTTSSTPRYRGIFPVVPTTFTEDGELDLESQKRCVDFMIDAGSDGLCILANFSEQFVLSDDEREVLTRTILEHVAGRVPVIVTTTHYSTRVCAERSR
IQtree-N10                 -MPSTTSSTPRYRGIFPVVPTTFTEDGELDLESQKRCVDFMIDAGSDGLCILANFSEQFALSDDEREVLTRTILEHVAGRVPVIVTTTHYSTRVCAERSR
IQtree-N11                 -M--TSSSTPRYRGIFPVVPTTFTDTGELDLASQKRAVDFMIDAGSDGLCILANFSEQFAITDDERDVLTRTILEHVAGRVPVIVTTSHYSTQVCAARSL
IQtree-N12                 -MT------KTYTGIWPVAPTPFNPDGTLDLEGMKRVLDCMIDQGVDGICILANFSEQFLISDAEREVLTRLSLEHVAGRVPVIVTISHYATQIAVERAQ
IQtree-N13                 -MS------KTYTGIWPVAPTPFNPDGTLDLEGMKRVLDCLIDQGADGICILANFSEQFLISDAEREVLTRLSVEHIAGRVPVIVTISHYATQIAVERAQ
IQtree-N14                 -MS------KTYTGIWPVAPTPFNPDGTLDLEGMKRVLDCLIDQGADGICILANFSEQFLISDAEREVLARLSVEHIAGRVPVIVTISHYATQIAVERAQ
IQtree-N15                 -MT------KTYTGIWPVAPTPFNPDGTLDLEGMKRVLDCMIDQGVDGICILANFSEQFLISDAEREVLTRLSLEHVAGRVPVIVTISHYATPIAVERAQ

KdaD_Cupriavidus-necator   AAQDAGAAMVMVMPPYHGATIRVPERSIYEFFATVSDAIDIPIMIQDAPVSGTTLSAPFLARMAREIGNVSYFKIEVPQAAAKLRELIELGGDAIVGPWD
IQtree-N2                  YAQDLGADMVMVMPPYHGALFRVPEEQIYEFFKAVSEAVDIPIMIQDAPVSGVDLSAPFLARMAREIENVSYFKIETAGAANKLRELIELGGDAIGGFSG
IQtree-N3                  RAQDMGAAMVMVMPPYHGATFRVPEKQIYEFFKRVSDAVDIPIMIQDAPVSGTPLSAPFLARMAREIENVSYFKIETAGAASKLRELIELGGDAIEGPWD
IQtree-N4                  RAQDAGAAMVMVMPPYHGATFRVPEKQIYEFFKRVSDAIDIPIMIQDAPVSGTPLSAPFLARMAREIENVSYFKIETAGAASKLRELIELGGDAIEGPWD
IQtree-N5                  RAQDAGAAMVMVMPPYHGATFRVPEKGIYEFFKRVSDAIDIPIMIQDAPVSGTPLSAPFLARMAREIANVSYFKIEVPGAASKLRELIELGGDAIEGPWD
IQtree-N6                  RAQDAGAAMVMVMPPYHGATFRVPEKGIVEFFKRVSDAIDIPIMIQDAPVSGTPLSAPLLARMAREIANVSYFKIEVPGAASKLRTLIELGGDAIEGPWD
IQtree-N7                  RAQDMGAAMVMVMPPYHGATFRVPEAQIYEFFKRVSDAVDIPIMIQDAPVSGTPLSAPFLARMAREIEHVSYFKIETAGAASKLRELIELGGDAIEGPWD
IQtree-N8                  RAQDMGAAMVMVMPPYHGATFRVPEAQIYEFFKRVSDAVDIPIMIQDAPVSGTPLSAPFLARMAREIEHVSYFKIETAGAASKLRELIELGGDAIEGPWD
IQtree-N9                  RAQDMGAAMVMVMPPYHGATFRVPEAQIYEFYARVSDAVDIPIMIQDAPASGTVLSAPFLARMAREIEHVAYFKIETAGAASKLRELIRLGGDAIEGPWD
IQtree-N10                 RAQDMGAAMVMVMPPYHGATFRVPEAQIYEFYARVSDAIDIPIMIQDAPASGTVLSAPFLARMAREIEHVAYFKIETPGAASKLRELIRLGGDAIEGPWD
IQtree-N11                 RAQQLGAAMVMAMPPYHGATFRVPEAQIFEFYARVSDAIDIPIMIQDAPASGTVLSAPFLARMAREIEQVAYFKIETPGAANKLRELIRLGGDAIEGPWD
IQtree-N12                 FAKDLGADIVMMMPPYHGALLKGTAEQTFEQFKAVGE-VGIPIMVQDAPLSGVDLPVPLLVRMAREIEMVKLFKIECPRAANKLRALIEQGGDAIEGPFD
IQtree-N13                 FAKDLGADIVMMMPPYHGALLKGTPEQSFEQFKAVGE-VGIPIMVQDAPLSGVDLPVPLLVRMAREIEMVKLFKIECPRAANKLRALIEQGGDAIEAPFD
IQtree-N14                 FAKDLGADIVMMMPPYHGALLKGTPEQSFEQFEAVGE-VGIPIMVQDAPLSGVDLPVPLLVRMAREIEAVKLFKIECPRAANKIRALIEQGGDAIEAPFD
IQtree-N15                 FAKDLGADIVMMMPPYHGALLKGTAEQTFEQFRAVGE-VGIPIMLQDAPLSGVDLPVPLLVRMAREIEMLKLFKIECPQAAMKLRALIEQGGDAIEGPFD

KdaD_Cupriavidus-necator   GEEAITLMADLEAGATGAMTG--AGFPDGIRQILDAWQAGDAELAAQRYQQWLPLINYENRQGWLATSKVLMQAGGVIASDAVRHPLQPMHPATREGLLK
IQtree-N2                  GEEVI--LPALLAGATGAMTGTSGVIPDLIRPIIDAFLAGDREKAVALYQQVLPLINYENRQCGLSTCKAAMQEGGVIKSDACRHPFPPLHPATREGLLE
IQtree-N3                  GEEAITLLPDLDAGATGAMTG--GGYPDGIRPIIDAYLAGDREKAVALYQQWLPLINYENRQCGLSTCKALMQEGGVIKSDAVRHPFPPLHPATREGLLE
IQtree-N4                  GEEAITLLPDLDAGATGAMTG--GGYPDGIRQIIDAYLAGDREKAVALYQQWLPLINYENRQCGLSTCKALMQEGGVIKSDAVRHPLPPLHPATREGLLE
IQtree-N5                  GEEAITLLADLDAGATGAMTG--GGYPDGIRQIIDAYLAGDREKAVAAYQQWLPLINYENRQCGLATCKVLMQEGGVIKSDAVRHPLQPLHPATREGLLE
IQtree-N6                  GEEAITLLADLDAGATGAMTG--GGYPDGIRQIIDPYLAGDREKAVAAYQRWLPLINYENRQCGLATCKVLMQEGGVIKSEAVRHPLQPLHPATREGLLE
IQtree-N7                  GEEAITLLPDLDAGATGAMTG--GGYPDGIRPIIDAHLAGDREKAVALYQQWLPLINYENRQCGILTCKALMQEGGVIASDAARHPFPAMHPATRAGLLE
IQtree-N8                  GEEAITLLPDLDAGATGAMTG--GGYPDGIRPIIEAHRAGDRDKAFALYQQWLPLINYENRQGGILTCKALMQEGGVIACDAPRHPFPAMHPETRAGLLE
IQtree-N9                  GEEAITLLPDLDAGATGAMTG--GGYPDGIRPIIEAHRAGDRDKAFALYQQWLPLINYENRQGGILTCKALMKEGGVIACEAPRHPFPAMHPETRAGLLE
IQtree-N10                 GEEAITLLADLDAGATGAMTG--GGFPDGIRPIIEAHREGDRDKAFALYQQWLPLINHENRQGGILTAKALMKEGGVIACEAPRHPFPAMHPETRAGLLE
IQtree-N11                 GEEAITLLADLHAGATGAMTG--GGFPDGIRPILEAWREGRRDDAFARYQAWLPLINHENRQSGILTAKALMREGGVIACERPRHPMPELHPDTRAELLA
IQtree-N12                 GEEAITLLADLDAGATGTMTS--GMIPDLIKPVVTDFLAGNREEAIAAYARVLPAINHENRQCGFRSCKAAMVEGGVIKSDFCRHPIPPLHPATRERLME
IQtree-N13                 GEEAITLLADLDAGATGSMTS--GMIVDQIKPVLTHHYAGRIEEATAAYGRVAMAINHENRQCGWQSCKAAMVEGGVIKSEFCRHPIPPLHPAIRERLID
IQtree-N14                 GEEAITLLADLDAGATGSMTS--GMIVDQIKPVLTHHYAGRIDEATAAYGRVAMAINHENRQCGWQSCKAAMVEGGVIKSEFCRHPIPPLHPAIRSRLID
IQtree-N15                 GEEAITLLADLDAGATGTMTS--AMIPDLIGPVVRDFLAGNRQAAIDGYARVLPAVNHENRQCGFRSAKAAMVEGGVIKSDFCRHPIPPLHPATRAMLME

KdaD_Cupriavidus-necator   IARRLDPLVLRWAR
IQtree-N2                  LLRRLDPLVLKWGR
IQtree-N3                  IARRLDPLVLRWGR
IQtree-N4                  IARRLDPLVLRWGR
IQtree-N5                  IARRLDPLVLRWGR
IQtree-N6                  IARRLDPLVLRWGR
IQtree-N7                  IARRLDPLVLRWGR
IQtree-N8                  IARRLDPLVLRWGR
IQtree-N9                  IARRLDPLVLRWGK
IQtree-N10                 IARRLDPLVLRWAK
IQtree-N11                 IARRLDPLVLRWAH
IQtree-N12                 LLRPLDPMVLKWGK
IQtree-N13                 LLKPLDPMVLKWGK
IQtree-N14                 LLKPLDPMVLKWGK
IQtree-N15                 LLRPLDPMVLKWGK
```

Figure S32: Multiple sequence alignment of *Cn*KdaD and all KdaD ancestors. The alignment was performed using Promals3D, active site residues are highlighted in green. The alignment was used as input for figure 3.

```
KdgD_Acinetobacter-baylyi   ---MDALELKNIVSDGLLSFPVTDFDQNGDFNAASYAKRLEWLAPYGASALFAAGGTGEFFSLTGDEYSDVIKTAVDACKGSVPIIAGAGGPTRQAILQA
IQ-N45                      ---MNPLELRQTVPSGVLSFPVTPFDADGEFDEDAYRKHVEWLLEDGAAALFAACGTGEFFSLTPDEYPQVVRAAVEATAGRVPVIAGAGYGTAQAIEYA
IQ-N46                      ---MNPQELKQTVSSGLLSFPVTHFDADGEFDEAAYRKHVEWLSGYGAAALFAAGGTGEFFSLTPDEYPQVVRAAVEATAGRVPVIAGAGYGTAQAIEYA
IQ-N47                      ---MNPQELKNIVSSGLLSFPVTDFDANGDFNAASYAKRLEWLAPYGASALFAAGGTGEFFSLTGDEYSEVIKTAVDTCRGSVPIIAGAGGPTRQAIEYA
IQ-N48                      ---MNPQELKNIVSSGLLSFPVTDFDANGDFNAASYAKRLEWLAPYGASALFAAGGTGEFFSLTGDEYSEIIKTAVDTCRGSVPIIAGAGGPTRQAIEYA
IQ-N49                      ---MNPQELKSILSSGLLSFPVTDFDAAGDFNAASYAKRLEWLAPYGASALFAAGGTGEFFSLAADEYSEIIKTAVDTCRGSVPILAGAGGPTRQAIEYA
IQ-N50                      ---MNPQELKSILSSGLLSFPVTDFDAAGDFNAASYAKRLEWLAPYGASALFAAGGTGEFFSLAADEYSEIIKTAVDTCAGSVPILAGVGGPTRQAIEYA
IQ-N51                      ---MNPQELKNIVSSGLLSFPVTDFDANGDFNAKSYAERLEWLAPYGASALFAAGGTGEFFSLTGDEYSEIIKTAVDTCRGKVPIIAGAGGPTRFAIEYA
IQ-N52                      ---MNPQDLKNIVSSGLLSFPVTDFDANGDFNAKSYAERLEWLAPYGASALFAAGGTGEFFSLTGDEYPQIIKTAVDTCRGKVPIIAGAGGPTRFAIACA
IQ-N53                      ---MTPQDLKNVMSSGLLSFPVTDFDANGDFNAKGYAERLEWLAPYGASALFAAGGTGEYFSLTGDEYPQIIKTAVDTCRGKVPIIAGAGGPTRFAIACA
IQ-N54                      ---MDALELKNIVSDGLLSFPVTDFDQNGDFNAASYAKRLEWLAPYGASALFAAGGTGEFFSLTGDEYSEVIKTAVDTCRGSVPIIAGAGGPTRQAIEQA
IQ-N55                      ---MNPQELKQTVGSGLLSFPVTHFDADLEFDEAAYRKHVEWLSGYDAAALFAAGGTGEFFSLTPDEVPQVVRAAVEATAGRVPVIAGAGYGTAIAIEIA
IQ-N56                      MSRYSPQELAQTVGSGLLSFPVTHFDADLEFDEAAYRKHIEWLSGYDAAGLFAAGGTGEFFSLTPDEVPQVVRAAVEATAGRVPVIAGAGYGTAIAIEIA
IQ-N57                      MSRYSPQEMAQTIGSGLLSFPVTHFKADLSFDEAAYRANIDWLSGYDAAGLFAAGGTGEFFSLTPAEVPQVVRAAVEETAGRVPVIAGAGYGTAIAIEIA
IQ-N58                      MSRYSPQEMAQTIGSGLLSFPVTHFKADLSFDEAAYRANIDWLSGHDAAGLFAAGGTGEFFSLTPAEVDQVVRAAVEETAGRLPVIAPAGYGTAIAIEMA
IQ-N59                      MSRYSPSEMAQTIGSGLLSFPVTHFKADLSFDEAAYRANLDWLSSHDAAGLFAAGGTGEFFSLTPAEVDRVVRAAVAETAGRLPVIAPAGYGTAIAIEMA
IQ-N60                      MSRYSPSEFAQQIGSGLLSFPVTHFKADLSFDEAAYRANLSWLFSHEAAGLFAAGGTGEFFSLTPAETDRVVRAAVAETAGRLPVIAPAGYGTAMAIEYC
IQ-N61                      MTTASPQEIAHKLGSGLLSFPVTHFRADHSFDEAAYRENIGWLGGHDAAGLFAAGGTGEFFSLTPAEVEQVVRAAVQEAPDGLPVIAPAGYGTATAIEMA
IQ-N62                      ---MNPQELKKAVGSGLLSFPVTHFDADLKFDEASYRRHVEWLSGYDAAALFAAGGTGEFFSLTPDEVPQVVRAA-KEAAGNVPIISGAGYGTAIAVEIA
IQ-N63                      ---MNPQELKTAVGSGLLSFPVTHFDADLKFNEASYRRHVEWLSGYDAAALFAAGGTGEFFSLTPDEVPQVVRAA-KEAAGNVPIISGCGYGTEIAVEIA
IQ-N64                      ---MSPQELKTRVGSGLLSFPVTHFNADFKLNLASYRRHVEWLSGYDAAALFAAGGTGEFFSLTPDEVGQVTRAA-KEAAGNVPIISGCGYGTELAVEIA
IQ-N65                      ---MNPQDLKTALGSGLLSFPVTHFDADGRFNEASYRQHVEWLAGYDAAALFAAGGTGEFFSLAPDEIPAIVRAA-KEAAGNTPIISGCGYGTEIAVEIA
IQ-N66                      ---MNPQDIKTALGSGLLSFPVTHFDADGRFNAESYRQHVEWLAGYDAPVLFAAGGTGEFFSLAPDEIPAIVSAA-KEAAGKTAIVSGCGYGTEIAVEIA
IQ-N67                      -MTSAPLELRQTVPSGVLSFPVTAFDADGGFDEDAYRTHVERRLEAGAAALFAACGTGEFFSLTPDEYPQVVRAAVEETAGRVPVIAGAGYGTAQAIRYA

KdgD_Acinetobacter-baylyi   QEAERLGAHGILLMPHYLTEASQEGLVEHVKQVCNAVNFGVIFYNRSVSKLNVDSLQQLVESCPNLIGFKDSSGQIDMMTEVVQTLGDRLSYLGGLPTAE
IQ-N45                      QLAEKAGADGILVLPPYLTEASQEGLYAHVKAVAESTNLPVIVYNRDNAVLTPETIARLAE-CPNLVGLKDGVGDLDLMTRIVALLGDRFTYFNGLPTAE
IQ-N46                      QAAEKAGADGILLLPPYLTEASQEGLYAHVKAVCESTNLGVIVYNRDNAVLTPDTIARLAERCPNLVGFKDGVGDIELMTRITAKLGDRFTYLGGLPTAE
IQ-N47                      QEAERLGAHGILLLPHYLTEASQEGLIAHVKAVCKSVNFGVIVYNRNVCRLTPDSLAKLAERCPNLIGFKDGVGDIELMTSITQKLGDRFSYLGGLPTAE
IQ-N48                      QEAERLGAHGILLLPHYLTEASQEGLIAHVEAVCKSVNFGVIVYNRNVCRLTPDSLAKLAERCPNLIGFKDGVGDIELMVSIRQKLGDRFSYLGGLPTAE
IQ-N49                      QEAERLGAKGILLLPHYLTEASQEGLVAHVEAVCKSVNFGVVVYNRNVCRLTPDSLEKLAERCPNLIGFKDGVGDIELMVSIRRRLGDRFSYLGGLPTAE
IQ-N50                      QEAERLGAKGLLLLPHYLTEASQEGVAAHVEAVCKSVNIGVVVYNRNVCRLTPALLEKLAERCPNLIGYKDGLGDIELMVSIRRRLGDRFSYLGGLPTAE
IQ-N51                      QEAERLGAHGILLLPHYLTEASQEGLIAHVEAVCKSVKFGVIVYNRNVCRLTPDSLAILAERCPNLIGFKDGVGDIETMVSIRQKLGDRFSYLGGLPTAE
IQ-N52                      QEAERLGAHGILLLPHYLTEAGQEGLIAHVEAVCKSVKFGVIVYNRNVCRLTPESLAILAERCPNLIGFKDGVGNIETMSSIFMKMGDRFSYLGGLPTAE
IQ-N53                      QEAERLGAHGILLLPHYLTEAGQEGLIAHVEAVCKSVKFGVIVYNRDVTRFTPESLAILAERCPNLVGFKDGVGNIETMSSIFMKMGDRFAYLGGLPTAE
IQ-N54                      QEAERLGAHGILLMPHYLTEASQEGLIEHVKQVCNSVNFGVIFYNRSVSRLNLDSLQKLTESCPNLIGFKDSSGQIDMMTAVTQTLGDRLSYLGGLPTAE
IQ-N55                      QAAEKAGADGILLLPPYLTEASQEGLYAHVKAVCESTNLGVIVYNRDNAVLTPDTIARLAERCPNLVGFKDGVGDIELMTRITAKLGDRLTYLGGLPTAE
IQ-N56                      QAAEKAGADGILLLPPYLTEASQEGLYAHVKAVCESTNLGVIVYNRDNAVLTPDTIARLAERCPNLVGFKDGVGDIELMTRITAKLGDRLTYLGGLPTAE
IQ-N57                      QAAEKAGADGILLLPPYLTEASQDGLAAHVEAVCAATNLGVIVYNRDNAVLTPDTLARLAERCPNLVGFKDGVGDIELMTRIYAKLGDRLTYVGGLPTAE
IQ-N58                      QAAERAGADGILLLPPYLTEASQDGLAAHVEQVCAATNLGVIVYNRANAVLTEDALARLAERCPNLVGFKDGVGDIELMTRIYARLGDRLTYVGGLPTAE
IQ-N59                      QAAERAGADGILLLPPYLTEASADGVAAHVEQVCKATNLGVIVYNRANAVLDEDALARLAERCPNLVGFKDGVGDIELMTRIYARLGDRLTYVGGLPTAE
IQ-N60                      QAAEAAGADGILLLPPYLTEASADGVAAHVEQVCKSTKLGVIVYNRANQVLDENHLERLAERCPNLVGFKDGVGDLELMTRIYARLGDRFTYVGGLPTAE
IQ-N61                      RAAERAGAHGILLLPPYLTEASQDGLVAHVKQVCAATNLGVIVYSRANAVYTEAAVAELADRCPNLVGFKDGVGNIEQMTRIYASLGDRLTYVGGLPTAE
IQ-N62                      KAAEKAGADGILLLPPYLMEAPQEGLYAHVKAVCESTGIGVIVYNRDNAVLNADTIARLADACPNLVGFKDGVGDIDLVTRITAKLGDRLTYLGGMPTHE
IQ-N63                      KAAEKAGADGILLLPHYLMEAPQEGLYAHVKAVCDSTGIGVIVYNRDNSVLNADTIARLADACPNLVGFKDGTGDIDLVRRITAKLGDRLTYLGGMPTHE
IQ-N64                      KAAEQAGADGILLLPHYLMEAPQEGIYAHVKAVCDSTGLGVIVYNRANSVANADTVARLADACPNLVGFKDGTGQIDLVRRITAKLGDRLSYIGGMPTHE
IQ-N65                      KAAEKAGADGILLLPHYLIDAPQEGLYAHIKKVCDSVGIGVMVYNRDNSVLQADTLARLCDACPNLVGFKDGTGDIGLVRQITAKMGDRLTYLGGMPTAE
IQ-N66                      KAAEKAGADGILLLPHYLIDAPQEGLYAHIKKVCDSVGIGVMVYNRDNSVLQADTLARLCDACPNLVGFKDGTGDIGLVRQITAKMGDRLIYLGGMPTAE
IQ-N67                      RLAEKAGADGLLVMPPYLTAASQEGLVAHVKAVAASTPLPVIVYQRDNAVFTPESVARLA-RTPGVVGLKDGLGDLDLMQRIVALPGDEFLYFNGLPTAE
```

```
KdgD_Acinetobacter-baylyi   IFAAPYKALGSPVYSSAVFNFIPKTAMEFYNALRNDDFATTQRLIRDFFLPLIKIRNRKSGYAVSMVKAGAKIVGHDAGPVRPPLSDLTPQDYEDLAALI
IQ-N45                      TTALPYLALGVTSYSSAVFNFVPELALDFYRALREGDDATVHRLLRDFFLPFVAIRNRGKGYAVSLVKAGMKLMGLDAGPVRPPLTDLTEEELEQLAALI
IQ-N46                      TYALPYLALGVTTYSSAVFNFVPELALDFYRAVRAGDHATVHRLLRDFFLPFVAIRNRKKGYAVSIVKAGMKVIGHDAGPVRPPLTDLTEEELEELAALI
IQ-N47                      VYAAPYKALGVPVYSSAVFNFIPKTAMDFYHAVRSDDHATVHRLIRDFFLPYLAIRNRKAGYAVSIVKAGAKIVGHDAGPVRPPLTDLTPAEMEELAALI
IQ-N48                      VYAAAYKALGVPVYSSAVFNFIPKTAMDFYHAVASDDHATVHRLIRDFFLPYLAIRNRKAGYAVSIVKAGAKIVGHDAGPVRPPLTDLTPAEMEELAALI
IQ-N49                      VYAAAYKALGVPVYSSAVFNFIPKTAMDFYHAVASDDHATVGRLIDDFFLPYLDIRNRKAGYAVSIVKAGAKIVGHDAGPVRAPLTDLTPDEMEQLAALI
IQ-N50                      VYAAAYKALGVPVYSSAVFNFIPKTAMDFYHAVASDDHATVGRLIDDFFLPYLDIRNRKAGYAVSIVKAGAKIVGHDAGPVRAPLTDLTPDEYEQLAALI
IQ-N51                      VYAAAYKALGVPVYSSAVFNFIPKTAMDFYHAVASDDHATVHRLIRDFFLPYLAIRNRNAGYAVSIVKAGAKIVGHDAGPVRPPLTDLKPAEMEELAALI
IQ-N52                      VYAAAYKALGTPVYSSAVFNFIPKTAMDFYHAVASDDLATQHRLLRDFFMPYLAIRNRNAGYAVSIVKAGAKIVGHDAGPVRPPLTDLKPAEMEELAALI
IQ-N53                      VYAAAYKALGTPVYSSAVFNFIPKTAMAFYEAVRSDDLATQHRLLKDFFMPYLKIRNRNAGYAVSIIKAGAKIVGHDAGPVRAPLTDLKPAEMEELAALI
IQ-N54                      VFAAPYKALGCPVYSSAVFNFIPKTAMEFYNALRSDDFATTHRLIRDFFLPLIKIRNRKSGYAVSMVKAGAKIVGHDAGPVRPPLSDLTPADYEDLAALI
IQ-N55                      TYALPYLAMGVTTYSSAVFNFVPELALDFYRAVRAGDHATVHRLLRDFFLPFVAIRNRKKGYAVSIVKAGMKVIGHDAGPVRPPLTDLTEEELAELAALI
IQ-N56                      TFALPYLAMGVTTYSSAVFNFVPEFALDFYAAVRAGDHATVHRLLRDFFLPFVAIRNRKKGYAVSIVKAGMKVIGRDAGPVRPPLTDLTEEELAELAALI
IQ-N57                      TFALPYLAMGVTTYSSAVFNFVPEFALDFYAAVRAGDHATVHRALNDFVLPLIALRNRKKGYAVSIVKAGMKVIGRDAGPVRPPLTDLTEEELAELTALV
IQ-N58                      TFALPYLAMGVTTYSSAIFNFVPEFALDFYAAVRAGDHATVYRALNDFVLPYIALRNRKRGYAVSIVKAGMKVIGRPAGPVRPPLTDLTEAELAELTALV
IQ-N59                      TFALPYLAMGVTTYSSAIFNFVPEFALDFYAAVRAGDHATVYRALNDFVLPYIALRNRKRGYAVSIVKAGMKVIGRPAGPVRAPLTDLTEAELAELTALV
IQ-N60                      TFALPYLTMGVTTYSSAIFNFVPKFALDFYAAVRAADHAKVYKMLNDFVLPYIALRNRKRGYAVSIVKAGMKVIGRSAGPVRAPLTDLTEAELAELSALV
IQ-N61                      MFALPYLALGVTTYSSAIFNFVPQFALDFYNAVRSGDNAFVLNALNDFVIPYCNLRNRKQGYAVSIIKAGMKVIGRPAGPVRPPLTDLDDAELAELADLV
IQ-N62                      LFAQAYLAAGVTTYSSAVFNFVPELALRFYRALRAGDRATVERILRDFFFPFVAIRNRKKGYAVSIIKAGVRVIGHDPGPVRPPLTDLTPEELAALAALI
IQ-N63                      LFAQAYLGAGVTTYSSAVFNFVPELALRFYRALRAGDRATVERILRDFFFPFVAIRNRKKGYAVSIIKAGVRLIGHDPGPVRPPLTDLTPEELAMLAALI
IQ-N64                      LFAQGFNGVGVTTYSSAVFNFVPELALRFYRALRAGDRATMERILHSFFFPFAAIRDRKKGYAVSIIKAGVELIGHAPGPVRPPLTDLTPEEREMLQALI
IQ-N65                      LFAEAYLGAGFTTYSSAVFNFVPELAVRFYRALRAGDRATCERILRDFFYPFMAIRNRRKGYAVSAIKAGVRLQGYDAGPVRAPLTDLTSEEMAMLAALI
IQ-N66                      LFAEAYLGAGFTTYSSAVFNFVPGLAVEFYRALRAGDRATCERILRDFFYPFMAIRNRRKGYAVSAIKAGVRLQGFDAGPVRAPLTDLTSEEMAMLDALI
IQ-N67                      TTQLPYRALGVTSYSSAVFNFAPEIAVAFYRALREGDDATVHRLLRDFYLPFVELRNRGRGYAVSLVKAGVRLRGLDVGPVRTPLTDPTEEELEQLAALI

KdgD_Acinetobacter-baylyi   ATLGPQ-----
IQ-N45                      KKAGVR-----
IQ-N46                      KKAGVR-----
IQ-N47                      KKLGPQ-----
IQ-N48                      KKLGPQ-----
IQ-N49                      KKLGPQ-----
IQ-N50                      KKLGPQ-----
IQ-N51                      KKLGPQ-----
IQ-N52                      KKLGPQ-----
IQ-N53                      KKLGPQ-----
IQ-N54                      ATLGPQ-----
IQ-N55                      KKAGV------
IQ-N56                      KKAG-------
IQ-N57                      KKVA-------
IQ-N58                      KKVA-------
IQ-N59                      ARVA-------
IQ-N60                      ARVADVQKLAA
IQ-N61                      KKVS-------
IQ-N62                      ETAKR------
IQ-N63                      ETAKR------
IQ-N64                      ETARR------
IQ-N65                      GTVKR------
IQ-N66                      GTVKR------
IQ-N67                      KKGQVLVEEGA
```

Figure S33: Multiple sequence alignment of *Ab*KdgD and all KdgD ancestors. The alignment was performed using Promals3D, active site residues are highlighted in blue. The alignment was used as input for figure 3.

```
N0_1271      ----------LLKLLGLIDVLLTPFEE-- -----DGEVDLEGLEKLCELLIEG-GFYGICIFGNFGEQFVLTEEERKEFIKLVLEALKG-EVPVAVVT----GHYDTEVAVYEAKLAEELGADGVDVVFPYRGLL-RGPEEGVFEFFKAVKEAC--ELPVLKYDIPAAL
N1__1310     -------PLGLKKLRGVIAVLPTPFDE-- -----DGEVDLEGLKKLVEFLIEQ-GVDGICILGNFGEQFVLTDEERKELIKTVLEAVNG-RVPVIVTT----GHYNTRVAVERAKYAEELGADGVMVVPPYHGPL-RVPEEGIYEFFKAVSEAV--DLPIMIYDIPAAS
N2__1311     -------PSSTKRYRGVFPVAPTPFDE-- -----DGELDLEGLKRVVDFMIDQ-GVDGICILANFSEQFVLSDDEREVLTKTVLEHVAG-RVPVIVTT----SHYSTRVAVERARYAQDLGADMVMVMPPYHGALFRVPEEQIYEFFKAVSEAV--DIPIMIQDAP-VS
N3_1306      --------SSTPRYRGVFPVAPTIFDE-- -----DGELDLEGQKRCVDFMIDA-GSDGLCILANFSEQFVLSDDEREVLTKTVLEHVAG-RVPVIVTT----THYSTRVCAERSRRAQDMGAAMVMVMPPYHGATFRVPEKQIYEFFKRVSDAV--DIPIMIQDAP-VS
N4_1306      --------SSTPPYRGVFPVAPTIFDE-- -----DGELDLEGQKRCIDFMIDA-GSNGLCILANFSEQFVLSDDEREVLTKTVLEHVAG-RVPVIVTT----THYSSRVCAERSRRAQDAGAAMVMVMPPYHGATFRVPEKQIYEFFKRVSDAI--DIPIMIQDAP-VS
N5_1339      ------RPSSTPPYRGVFPVAPTIFDE-- -----QGELDLEGQKRCIDFMIDA-GSHGLCILANFSEQFVLSDDEREVLMKTVLEHVAG-RVPVIVTT----THFSSRVCAERSRRAQDAGAAMVMVMPPYHGATFRVPEKGIYEFFKRVSDAI--DIPIMIQDAP-VS
N6_1312      ------RPSSTRPYRGVFPVAPTIFDE-- -----QGELDLEGQKRCIDFMIDA-GSHGLCILANFSEQFVLTDDEREVLMKTVLEHVAG-RVPVIVTT----THFSSRVCAERSRRAQDAGAAMVMVMPPYHGATFRVPEKGIVEFFKRVSDAI--DIPIMIQDAP-VS
N7_1311      ------TTSSTPRYRGVFPVVPTIFTE-- -----DGELDLEGQKRCVDFMIDA-GSDGLCILANFSEQFVLSDDEREVLTKTILEHVAG-RVPVIVTT----THYSTRVCAERSRRAQDMGAAMVMVMPPYHGATFRVPEAQIYEFFKRVSDAV--DIPIMIQDAP-VS
N8_1311      ------TTSSTPRYRGIFPVVPTTFTE-- -----DGELDLESQKRCVDFMIDA-GSDGLCILANFSEQFVLSDDEREVLTRTILEHVAG-RVPVIVTT----THYSTRVCAERSRRAQDMGAAMVMVMPPYHGATFRVPEAQIYEFFKRVSDAV--DIPIMIQDAP-VS
N9_1311      ------TTSSTPRYRGIFPVVPTTFTE-- -----DGELDLESQKRCVDFMIDA-GSDGLCILANFSEQFVLSDDEREVLTRTILEHVAG-RVPVIVTT----THYSTRVCAERSRRAQDMGAAMVMVMPPYHGATFRVPEAQIYEFYARVSDAV--DIPIMIQDAP-AS
N10_1311     ------TTSSTPRYRGIFPVVPTTFTE-- -----DGELDLESQKRCVDFMIDA-GSDGLCILANFSEQFALSDDEREVLTRTILEHVAG-RVPVIVTT----THYSTRVCAERSRRAQDMGAAMVMVMPPYHGATFRVPEAQIYEFYARVSDAI--DIPIMIQDAP-AS
N11_1309     -----MTSSSTPRYRGIFPVVPTTFTD-- -----TGELDLASQKRAVDFMIDA-GSDGLCILANFSEQFAITDDERDVLTRTILEHVAG-RVPVIVTT----SHYSTQVCAARSLRAQQLGAAMVMAMPPYHGATFRVPEAQIFEFYARVSDAI--DIPIMIQDAP-AS
N12_1304     ----------TKTYTGIWPVAPTPFNP-- -----DGTLDLEGMKRVLDCMIDQ-GVDGICILANFSEQFLISDAEREVLTRLSLEHVAG-RVPVIVTI----SHYATQIAVERAQFAKDLGADIVMMMPPYHGALLKGTAEQTFEQFKAVGEV---GIPIMVQDAP-LS
N13_1304     ----------SKTYTGIWPVAPTPFNP-- -----DGTLDLEGMKRVLDCLIDQ-GADGICILANFSEQFLISDAEREVLTRLSVEHIAG-RVPVIVTI----SHYATQIAVERAQFAKDLGADIVMMMPPYHGALLKGTPEQSFEQFKAVGEV---GIPIMVQDAP-LS
N14_1304     ----------SKTYTGIWPVAPTPFNP-- -----DGTLDLEGMKRVLDCLIDQ-GADGICILANFSEQFLISDAEREVLARLSVEHIAG-RVPVIVTI----SHYATQIAVERAQFAKDLGADIVMMMPPYHGALLKGTPEQSFEQFEAVGEV---GIPIMVQDAP-LS
N15_1304     ----------TKTYTGIWPVAPTPFNP-- -----DGTLDLEGMKRVLDCMIDQ-GVDGICILANFSEQFLISDAEREVLTRLSLEHVAG-RVPVIVTI----SHYATPIAVERAQFAKDLGADIVMMMPPYHGALLKGTAEQTFEQFRAVGEV---GIPIMLQDAP-LS
IQN16_1334   -------PLGLKKLRGVIAALPTPFDE-- -----DGEVDLEALRKLVEFLIEQ-GVDGIFVSGTFGESFTLTHEERKELIKTVVEAVNG-RVPVIVGT----GSLNTREAIERAKYAEELGADGVMVVPPYYSP---LSEEGIYQYFKAVAEAV--DLPIMIYDIPAAS
N17_1316     -----TVKLGVKDLRGVVAAIPTPSKPDA ---NAANTVDLEALARLVEALIRD-GVDGILTTGTFGESHTLTWEERKALIGTVVETVNG-RVPVFVGA----STLNTRDTIERARYAEDLGADGVMVGPPMYSP---LSAEGIVQYYKDVAEAV-PEMAIMIYDNPEAF
N18_1327     -----TTKLGVDDIRGVVAIIPTPSKPDA NELGAANTVDLDETARMVEALIRD-GVDGILTTGTFGECATLTWEERQAFIGTVVETVSG-RVPVFVGA----TTLNTRDTIERTRYAEDLGADGVMVGRPMWSP---LDAEGIVQFYRDVAEAV-PEMAIVIYDNPEAF
N19_1323     -----TTRLGADDIKGAWAIIPTPSKPDA SDWRATNTVDLDETARIVEALIDA-GVNGILSMGTFGECATLTWEEKQDFIGTVVETVRG-RVPVFVGT----TTLNTRDTIEQTRYALDIGADGTMLGVPMWSP---LDVEAAVQFYRDVAEAV-PEMAIAIYANPEAF
N20_1330     -----TGLLNADDIKGAWAIIPTPSKPDA SDWRATNTVDLDETARVVEGLIDA-GVNGILSMGTLGECATLTWEEKQDFIKTIVETARG-RVPVFVGT----TTLNTRDTIEQTRYALDIGADGTMLGVPMWCA---PSVDVAVQFYKDVAEAV-PEMNIAIYANPEAF
N21_1329     -----TTRLGAEDINGAWAIMPTPSKPDA SDWRATNTVDLDETARIVEALIDA-GVNGILSMGTFGECATLTWEEKQDFIGTVVETIRG-RVPFFCGT----TALNTREVIRQTRAALDIGADGTMLGVPMWSR---MDVPAAVQFYRDVAEAV-PEAAIAIYANPEAF
N22_1327     -----DTKLGVDDIRGVVAIIPTPSKPGA DELGAADTVDLDETARMVEALIRD-GVDGILTTGTFGECATLTWEERQAFIGTVVETVSG-RIPVFVGA----TTLNTRDTIERTRYLEDLGADGVFVGRPMWSP---LDAEGIVQFYRDVAEAV-PDMAIVIYDNPEAF
N23_1330     -----DTKLTVDDITGVVGIIPTPSKPGA DEPGAADTVDLDETARMVEAMVRG-GVDVLLTTGTFGECATLTWEELQAFIGTVVETVAG-RIPVFVGA----TTLNTRDTIARSRYLEDLGADGLFVGRPMWLP---LDDEGIVQFYRDVAEAV-PDMAIVVYDNPGAF
IQN24_1334   -------PLGLKKLRGVIAALVTPFDE-- -----DGEVDQEALRKLVEFLIEQ-GVDGIFVSGTTGESFTLTHEERKELIKTVVEAVNG-RVPVIAGT----GSLNTREAIELAKYAEELGADGVMVVPPYYSP---LSEEGIYQYFKAVAEAA--DLPIIIYNIPART
N25_1296     -------------LKGIIAALITPFDE-- -----DGEIDQEALRKLVEYLIEQ-GVDGIFVSGTTGESFSLSLEERKELIKTVVEAAKG-RVKVIAHV----GSLNTKEAIELAKYAEELGADGIAAIPPFYFP---LSEEEILQYFKAIAEAA--DLPMIIYNIPALT
N26_1293     -------------LKGIIAALITPFDE-- -----DGEIDQEALRKHVEYLIEQ-GVDGIFVSGTTGEGFSLSLEERKELIKTVVEAAKG-RDKVIVHV----GSLNLKEAIELAKYAEELGADGIAAIPPFYFP---LSEEEILQYFKAIAEAA--DLPMFIYNIPALT
N27_1290     -------------MPEIIAPVITPFKG-- ------GKIDREKFKNHVENLIRK-GIDKVFVAGTTGLGPSLSLQEKMELIKAVSDVAD----KVIVQV----GSLNLEEAIELAKYAKDLGADGIAALPPYYFP--RLSEEQIVKYFKEISEVV--PLPVFIYNYPALT
N28_1291     -------------MPLKIVPVITPFKG-- ------GKIDGEKFKDHCEKLLRN-GIDKVFVAGTTGLGPSLSFQEKMELIKAVSDVAD----KVIVQV----GSLNLEESIELARIAKDMGFYGIAALPPYYFG--GLPEEWIIKYFTEISEVI----PTFIYNYPKLT
N29_1288     -------------MPLKIVPVITPFKG-- ------GKIDGEKFKDHCLKLLRN-GIDKVFVAGTTGLGPSLSFQEKMELIKAVSDVAD----KVIVQV----GSLNLEESIELARIAKDMGFYGIAALPPYYFG--GIPEEWIIKYYTEISEVI----PTFIYNYPKFT
N30_1286     -------------MPEIIAPVITPFKG-- ------GKIDREKFKNHVENLIRK-GIDKVFVAGTTGLGPSLSLQEKMELIKAVSDVAN----KVIVQV----GSLNLDEAIELAKYAKDLGADGIASLPPYYFP--RLSEKQIVKYFKELCEVV--PLPVFLYNYPAAT
N31_1307     --------LRLKKLQGLVAATITPLTE-- -----DGEINLSVIGQYVDYLIEEQGVKSIFVNGTTGEGFSLSVDERKQLAEAWVQKGKGKLDQVIVHV----GSLSLKESQELARHAAEIGADGIAVISPFYFK---PINKDILRNFLKEVAAAAPALPMYYYHIPALT
N32_1319     --------FPKKKLQGLVAATITPMTE-- -----NGEINFSVIGQYVDYLVEEQGVKNIFVNGTTGEGLSLSLSVSERRQVAEEWVTKGKDKLDQVIIHV----GALSLKESQELAQHAAEIGADGIAVIAPFFLK---PWNKDILINFLKEVAAAAPALPFYYYHIPALT
N33_1296     -------------LKGIIAALITPFDE-- -----DGEIDQEALRKLVEYLIEQ-GVDGIYVSGSTGESFSLSLEERKEIIKTVVEAAKG-RVKVIAHV----GSLNTKEAIELAKYAEELGADAIAAIPPFYFP---LSEEEILQYFKAIAEAA--DLPMIIYNIPALT
N34_1289     -------------LKGIYAALLTPFDE-- -----DGEIDQEALRKLVEYNIEQ-GVDGIYVGGSTGESFLLSLEERKEILKTVAEAAKG-RVKLIAHV----GSLNTKEAIELAKYAEELGYDAISAIPPFYYP---FSFEEILQYYKAIAEAA--DLPMIIYNIPALT
N35_1291     -------------LKGIYAALLTPFDE-- -----DGQIDQEALRKIVEYNIEKQKVDGIYVGGSTGESFLLSTEERKEIFKTVAEAAKG-RVKLIAQV----GSLNVKEAIELAKYAEELGYDAISAITPFYYP---FSFEEIKQYYKTIAEAA--DLPMIIYNIPALT
N36_1289     -------------LKGIYAALLVPFDE-- -----DGQINEEGLRQIVKHNIEKQKVDGLYVGGSTGENFLLSTEEKKEIFKIAKEAAKG-KVKLIAQV----GSLNLKEAIELGKYATELGYDALSAVTPFYYP---FSFEEIKQYYETIIEAT--DNKMIIYSIPALT
N37_1289     -------------LKGIYSALLVSFDE-- -----DGNINEKGLRQIIRHNIDKMKVDGLYVGGSTGENFMLSTEEKKEIFKIAKDEAKG-QVKLIAQV----GSVNLKEAVELGKYATELGYDALSAVTPFYYK---FSFEEIKHYYETIIEAT--DNKMIIYSIPFLT
N38_1293     -------------LKGIFSALLVSFNE-- -----DGSINEKGLRQIIRHNIDKMKVDGLYVGGSTGENFMLSTEEKKEIFRIAKDEAKD-QIALIAQV----GSVNLKEAVELGKYATELGYDSLSAVTPFYYK---FSFPEIKHYYDTIIEAT--GNNMIVYSIPFLT
N39_1289     -------------MKGIYSALLVSFDK-- -----DGNINEKGLREIIRHNIDVCKVDGLYVGGSTGENFMLSTDEKKRIFEIAMDEAKG-QVKLIAQV----GSVNLKEAVELAKFATDLGYDALSAVTPFYYK---FSFEEIKHYYETIINSV--DNKLIIYSIPFLT
N40_1293     -------------LKGLYAALLVPFDE-- -----NGQVNEEGLKQIAKNAIENEELDGLYVNGSSGENFLLNTEQKKQIFKIAKEAVGD-KVKLIAQV----GSLDLNEAIELGKYATELGYDALSAVTPFYYP---FSFEEIKQYYFDIIEAT--QNKMIIYSIPDLT
N41_1289     -------------LKGIYAALLTPFDE-- -----DGEIDQEALRKLVEYNIEQ-GVDGIYVGGSTGEAFLQSLEERKEILKTVAEAAKG-RVKLIAHV----GSLNTKEAIELAKYAEELGYDAISAIPPFYYP---FSFEEILQHYKAIAEAA--DLPMIIYNIPALT
N42_1297     ----------ATNLRGVMAALLTPFDQ-- -----QQALDKASLRRLVQFNIQQ-GIDGLYVGGSTGEAFVQSLSEREQVLEIVAEEGKG-KIKLIAHV----GCVTTAESQQLAASAKRYGFDAVSAVTPFYYP---FSFEEHCDHYRAIIDSA-DGLPMVVYNIPALS
IQN43_1333   -------PLGQMMLRGVITALVTPFDE-- -----DGEVDQEALRKLVEFLIEN-GVDGIFVSGTTGESFTLTHEEHKELIKTVVEAVNG-RVPVIAGT----GSLNTREAIELAKYAEEVGADGVLVVPPYYSK---PSQEGLYQHFKAVAEAA--DLPIIIYNIPGRT
IQN44_1300   ------LELRQTVPSGVLSFPVTPFDA-- -----DGEFDEDALRKHVEFLLED-GAEAIFVACGTGEFFSLTPDEYPQVVRAAVEATAG-RVPVIAGA----GS-NTAQAIEYAQLAEKAGADGILVLPPYLTE---ASQEGLYQHVKAVAEST--NLPVIVYNRD---
IQN45__1302  ---MNPLELRQTVPSGVLSFPVTPFDA-- -----DGEFDEDAYRKHVEWLLED-GAAALFAACGTGEFFSLTPDEYPQVVRAAVEATAG-RVPVIAGA----GY-GTAQAIEYAQLAEKAGADGILVLPPYLTE---ASQEGLYAHVKAVAEST--NLPVIVYNRD---
IQN46_1303   ---MNPQELKQTVSSGLLSFPVTHFDA-- -----DGEFDEAAYRKHVEWLSGY-GAAALFAAGGTGEFFSLTPDEYPQVVRAAVEATAG-RVPVIAGA----GY-GTAQAIEYAQAAEKAGADGILLLPPYLTE---ASQEGLYAHVKAVCEST--NLGVIVYNRD---
IQN47_1303   ------QELKNIVSSGLLSFPVTDFDA-- -----NGDFNAASYAKRLEWLAPY-GASALFAAGGTGEFFSLTGDEYSEVIKTAVDTCRG-SVPIIAGA----GG-PTRQAIEYAQEAERLGAHGILLLPHYLTE---ASQEGLIAHVKAVCKSV--NFGVIVYNRN---
IQN48_1303   ------QELKNIVSSGLLSFPVTDFDA-- -----NGDFNAASYAKRLEWLAPY-GASALFAAGGTGEFFSLTGDEYSEIIKTAVDTCRG-SVPIIAGA----GG-PTRQAIEYAQEAERLGAHGILLLPHYLTE---ASQEGLIAHVEAVCKSV--NFGVIVYNRN---
IQN49_1303   ------QELKSILSSGLLSFPVTDFDA-- -----AGDFNAASYAKRLEWLAPY-GASALFAAGGTGEFFSLAADEYSEIIKTAVDTCRG-SVPILAGA----GG-PTRQAIEYAQEAERLGAKGILLLPHYLTE---ASQEGLVAHVEAVCKSV--NFGVVVYNRN---
IQN50_1303   ------QELKSILSSGLLSFPVTDFDA-- -----AGDFNAASYAKRLEWLAPY-GASALFAAGGTGEFFSLAADEYSEIIKTAVDTCAG-SVPILAGV----GG-PTRQAIEYAQEAERLGAKGLLLLPHYLTE---ASQEGVAAHVEAVCKSV--NIGVVVYNRN---
IQN51_1303   ------QELKNIVSSGLLSFPVTDFDA-- -----NGDFNAKSYAERLEWLAPY-GASALFAAGGTGEFFSLTGDEYSEIIKTAVDTCRG-KVPIIAGA----GG-PTRFAIEYAQEAERLGAHGILLLPHYLTE---ASQEGLIAHVEAVCKSV--KFGVIVYNRN---
IQN52_1303   ------QDLKNIVSSGLLSFPVTDFDA-- -----NGDFNAKSYAERLEWLAPY-GASALFAAGGTGEFFSLTGDEYPQIIKTAVDTCRG-KVPIIAGA----GG-PTRFAIACAQEAERLGAHGILLLPHYLTE---AGQEGLIAHVEAVCKSV--KFGVIVYNRN---
IQN53_1303   ------QDLKNVMSSGLLSFPVTDFDA-- -----NGDFNAKGYAERLEWLAPY-GASALFAAGGTGEYFSLTGDEYPQIIKTAVDTCRG-KVPIIAGA----GG-PTRFAIACAQEAERLGAHGILLLPHYLTE---AGQEGLIAHVEAVCKSV--KFGVIVYNRD---
IQN54_1303   ------LELKNIVSDGLLSFPVTDFDQ-- -----NGDFNAASYAKRLEWLAPY-GASALFAAGGTGEFFSLTGDEYSEVIKTAVDTCRG-SVPIIAGA----GG-PTRQAIEQAQEAERLGAHGILLMPHYLTE---ASQEGLIEHVKQVCNSV--NFGVIFYNRS---
IQN55_1302   ---MNPQELKQTVGSGLLSFPVTHFDA-- -----DLEFDEAAYRKHVEWLSGY-DAAALFAAGGTGEFFSLTPDEVPQVVRAAVEATAG-RVPVIAGA----GY-GTAIAIEIAQAAEKAGADGILLLPPYLTE---ASQEGLYAHVKAVCEST--NLGVIVYNRD---
IQN56_1304   ----SPQELAQTVGSGLLSFPVTHFDA-- -----DLEFDEAAYRKHIEWLSGY-DAAGLFAAGGTGEFFSLTPDEVPQVVRAAVEATAG-RVPVIAGA----GY-GTAIAIEIAQAAEKAGADGILLLPPYLTE---ASQEGLYAHVKAVCEST--NLGVIVYNRD---
IQN57_1304   ----SPQEMAQTIGSGLLSFPVTHFKA-- -----DLSFDEAAYRANIDWLSGY-DAAGLFAAGGTGEFFSLTPAEVPQVVRAAVEETAG-RVPVIAGA----GY-GTAIAIEIAQAAEKAGADGILLLPPYLTE---ASQDGLAAHVEAVCAAT--NLGVIVYNRD---
IQN58_1304   ----SPQEMAQTIGSGLLSFPVTHFKA-- -----DLSFDEAAYRANIDWLSGH-DAAGLFAAGGTGEFFSLTPAEVDQVVRAAVEETAG-RLPVIAPA----GY-GTAIAIEMAQAAERAGADGILLLPPYLTE---ASQDGLAAHVEQVCAAT--NLGVIVYNRA---
IQN59_1304   ----SPSEMAQTIGSGLLSFPVTHFKA-- -----DLSFDEAAYRANLDWLSSH-DAAGLFAAGGTGEFFSLTPAEVDRVVRAAVAETAG-RLPVIAPA----GY-GTAIAIEMAQAAERAGADGILLLPPYLTE---ASADGVAAHVEQVCKAT--NLGVIVYNRA---
IQN60_1311   ----SPSEFAQQIGSGLLSFPVTHFKA-- -----DLSFDEAAYRANLSWLFSH-EAAGLFAAGGTGEFFSLTPAETDRVVRAAVAETAG-RLPVIAPA----GY-GTAMAIEYCQAAEAAGADGILLLPPYLTE---ASADGVAAHVEQVCKST--KLGVIVYNRA---
IQN61_1304   ----SPQEIAHKLGSGLLSFPVTHFRA-------DHSFDEAAYRENIGWLGGH-DAAGLFAAGGTGEFFSLTPAEVEQVVRAAVQEAPD-GLPVIAPA----GY-GTATAIEMARAAERAGAHGILLLPPYLTE---ASQDGLVAHVKQVCAAT--NLGVIVYSRA---
IQN62_1301   -----PQELKKAVGSGLLSFPVTHFDA-------DLKFDEASYRRHVEWLSGY-DAAALFAAGGTGEFFSLTPDEVPQVVRAA-KEAAG-NVPIISGA----GY-GTAIAVEIAKAAEKAGADGILLLPPYLME---APQEGLYAHVKAVCEST--GIGVIVYNRD---
IQN63_1301   -----PQELKTAVGSGLLSFPVTHFDA-------DLKFNEASYRRHVEWLSGY-DAAALFAAGGTGEFFSLTPDEVPQVVRAA-KEAAG-NVPIISGC----GY-GTEIAVEIAKAAEKAGADGILLLPHYLME---APQEGLYAHVKAVCDST--GIGVIVYNRD---
IQN64_1301   ---MSPQELKTRVGSGLLSFPVTHFNA-------DFKLNLASYRRHVEWLSGY-DAAALFAAGGTGEFFSLTPDEVGQVTRAA-KEAAG-NVPIISGC----GY-GTELAVEIAKAAEQAGADGILLLPHYLME---APQEGIYAHVKAVCDST--GLGVIVYNRA---
IQN65_1301   ---MNPQDLKTALGSGLLSFPVTHFDA-------DGRFNEASYRQHVEWLAGY-DAAALFAAGGTGEFFSLAPDEIPAIVRAA-KEAAG-NTPIISGC----GY-GTEIAVEIAKAAEKAGADGILLLPHYLID---APQEGLYAHIKKVCDSV--GIGVMVYNRD---
IQN66_1301   ---MNPQDIKTALGSGLLSFPVTHFDA-------DGRFNAESYRQHVEWLAGY-DAPVLFAAGGTGEFFSLAPDEIPAIVSAA-KEAAG-KTAIVSGC----GY-GTEIAVEIAKAAEKAGADGILLLPHYLID---APQEGLYAHIKKVCDSV--GIGVMVYNRD---
IQN67_1309   -----PLELRQTVPSGVLSFPVTAFDA-------DGGFDEDAYRTHVERRLEA-GAAALFAACGTGEFFSLTPDEYPQVVRAAVEETAG-RVPVIAGA----GY-GTAQAIRYARLAEKAGADGLLVMPPYLTA---ASQEGLVAHVKAVAAST--PLPVIVYQRD---
N68_1302     -----ELGQM--MFRGVITALVTPFDE-------DGEVDYEALRKLVEFLIEN-GVDGIVVCGTTGESPTLTHEEHKELIKTVVEAVNG-RVPVIAGT----GSNNTREAIELTKHAEEVGADGVLVVTPYYNK---PSQEGLYQHFKAVAEAA--DLPIILYNIPGRT
N69_1304     ----PLLELGAMNFGRVITAMVTPFDE-------DGNVDFEALQKLVDYLIDN-GSDGLVVCGTTGESPTLTHEEKIELFKTVVEAVNG-RVPVIAGT----GSNNTREAIELTKKAEEVGADGVLVVTPYYNK---PSQEGLYQHFKAVAEAA--DLPVMLYNIPGRT
N70_1304     ----PLLELGAMNFGRVLTAMVTPFDG-------DGNVDFEAAQKLADYLVDN-GSDGLVVCGTTGESPTLTHEEKIELFKTVREAVNG-RVPVIAGT----GSNNTREAIELTKKAEEVGADGVLVVTPYYNK---PPQEGLYQHFKAVAEAA--DLPVMLYNIPGRT
N71_1302     -----PELGAM-SFGRVLTAMVTPFDG-------DGNVDFAAAQKLADYLVDH-GSDGLVVCGTTGESPTLSWEEKYELFETVREAVNG-RAKVIAGT----GSNSTREAIEATKKAAEVGADGVLVVTPYYNK---PPQEGLYQHFRAVAQAA--DLPVMLYNIPGRT
N72_1295     ---------GAAHFGRVLTAMVTPFDG-------DGSLDFDAAQKLADYLVDN-GSDGLVVSGTTGESPTLTDEEKIELVRAVREAVGG-RVPVIAGT----GSNDTRHAIELTKKAEEAGADGVLVVTPYYNK---PPQEGLYAHFKAVAEAA--ELPVMLYDIPGRT
N73_1302     ----GTFDTGAAHFGRVLTAMVTPFDG-------DGSLDFDAAQKLADYLVDN-GSDGLVVSGTTGESPTTTDEEKIELVRAVREAVGG-RVPVIAGT----GSNDTRHAIELAKKAEEAGADGVLVVTPYYSK---PPQEGLYAHFKAVAEAA--ELPVMLYDIPGRT
N74_1302     ----GTFDTGAAHFGRVLTAMVTPFTG-------DGSLDLDAAQRLADHLVDK-GCDGLVVSGTTGESPTTTDAEKIELVRAVREAVGD-RAPVIAGV----GTNDTRHSIELAKAAEEAGADGLLVVTPYYSK---PPQEGLYAHFTAVADAA--ELPVMLYDIPGRS
N75_1301     -----GFDTGAAHFGTVLTAMVTPFTG-------DGSLDIAAAQRLADHLVDK-GCDGLVVSGTTGESPTTTDAEKIELLRAVREAVGD-RARVIAGV----GTNDTRHSIELAKAAAAAGADGLLVVTPYYSK---PPQEGLYAHFTAVADAT--ELPVMLYDIPGRS
N76_1300     -----GFDV-AARLGTLLTAMVTPFSG-------DGSLDTATAARLANHLVDQ-GCDGLVVSGTTGESPTTTDGEKIELLRAVLEAVGD-RARVIAGA----GTYDTAHSIRLAKACAAEGAHGLLVVTPYYSK---PPQRGLQAHFTAVADAT--ELPMLLYDIPGRS
N77_1299     ----G---TGAAPFGRVLTAMVTPFTG-------DGSLDLDGAQRLADHLVDK-GCDGLVVSGTTGESPTTTDAEKIELVRAVREAVGD-RAPVVAGV----GTNDTRHSIELAKKAEEKAGADGLLVVTPYYSK---PPQEGLYAHFTAVADAA--GLPVMLYDIPGRS
N78_1291     -----------MNFGKVITAMVTPFDE-------DGNVDFEALQKLVDYLIDN-GSDGLVVAGTTGESPTLTHEEKIELFKTVVEAVNG-RVPVIAGT----GSNNTRESIELTKKAEEVGVDGVLVVTPYYNK---PSQEGLYQHFKAVAEAT--DLPVMLYNIPGRT
N79_1295     -----------MNFGKVITAMVTPFDE-------QGNVDFEALSKLVDYLIDN-GSDGLVVAGTTGESPTLTHEEKIALFKTVVEAVNG-RVPVIAGT----GSNNTRASIELTKKAEEVGVDGVMLVTPYYNK---PSQEGLYQHFKAIAEST--SLPVMLYNIPGRT
N80_1295     -----------MNFGKVLTAMVTPFDE-------QGNVDFEALSKLVDYLIDN-GSDGLVVAGTTGESPTLTHEEKIALFEYVVKAVNG-RVPVIAGT----GSNNTRASIELTKKAEEVGVDGVMLVTPYYNK---PSQEGMYQHFKAIAEST--SLPVMLYNVPGRT
N81_1289     -----------MNFGNVLTAMVTPFDE-------KGNVDFEKLSKLVDYLIDN-GTDGLVVAGTTGESPTLTHEEKIALFEYVVKAVNG-RVPVIAGT----GSNNTRASIELTKKAEEVGVDGVMLVTPYYNK---PSQEGMYQHFKAIAEST--SLPVMLYNVPGRT
N82_1290     -----------MNFGNVSTAMITPFDN-------KGNVDFQKLSTLIDYLLKN-GTDSLVVAGTTGESPTLSTEEKIALFEYTVKEVNG-RVPVIAGT----GSNNTKDSIKLTKKAEEAGVDAVMLVTPYYNK---PSQEGMYQHFKAIAAET--SLPVMLYNVPGRT
N83_1292     -----------MDFGNVLTAMVTPFDE-------KGNVDFEKLSKLVDYLIDN-GTDGLVVAGTTGESPTLTHEEKIALFEYVVKVVNK-RVPVIAGT----GSNNTHASIELTKKATEVGVDGVMLVTPYYNK---PSQEGMYQHFKAIAEST--SLPVMLYNVPGRS
N84_1291     -----------MNFGKVITAMVTPFDE-------DGNVDFEALQKLVDHLIDN-GSDGLVVAGTTGESPTLTHEEKIELFKTVVEAVNG-RVPLIAGT----GSNNTRESIEFTKEAAEVGVDAVLVVTPYYNK---PSQEGLYQHFKAVAEAS--DLPVMIYNIPGRT
N85_1302     -----ELGQM--MFRGVITALVTPFKE-------DGEVDYEALRKLVEFQIEN-GVDGIVVCGTTGESPTLTHEEHKELIKTVVEAVNG-RVPVIAGT----GSNNTREAIELTKHAEEVGADGVLVVTPYYNK---PSQEGLYQHFKAVAEAA--DLPIILYNIPGRT
N86_1302     -----ELGQM--MFRGVITALVTPFKE-------DGEVDYEALRKLVEFQIEN-GVDGIVVCGTTGESPTLTHEEHKELIKTVVEAVNG-RVPVIAGT----GSNNTREAIELTKHAAEVGVDGVLVVTPYYNK---PSQEGLYQHFKAVAEAA--DLPIILYNIPGRT
N87_1302     -------ELGQMMFRGVITALVTPFKE-------DGEVDYEALRKLVEFQIEN-GVDGIVVCGTTGESPTLTHEEHKELIKTVVEAVGG-RVPVIAGT----GSNNTREAIELTKHAAEVGVDGVLVVTPYYIK---PSQEGLYAHFKAVAEAA--DLPIILYNIPGRT
N88_1302     -----ELGQM--MFRGVWTALVTPFKE-------DGEVDYEALRKLVEFQIEN-GVDGIVVCGTTGESPTLTHEEHKELIKTVVEAV-G-RVPVIVGT----GGNNTREAIELLKHAAEVGVDGVLVVTPYYIK---PSQEGLYAHFKAVAEAA--DLPIILYNIPGRT
N89_1302     ------PDPGQPMFRGIWIPLVTPFRD-------DGEVDYEALRKLVEHQLEA-GVDGLVVCGTTGEAATLTEEEQKALIKAVVEAVG--RVPVIVGL----GGNNTREAIELLKRLAERGVDGVLVPAPYYIR---PSQAGLLAHFAAVAEAA--ALPIILYNIPYRT
N90_1301     ------PDPGQPMFRGIWIPLVTPFR--------DGEVDYEALRKLVEHYLEA-GVDGLVVCGTTGEAATLTEAEQKALIKAVVEAVG--RLPVIMGL----SGNNTREVIELLARLAERPIDGVLVPAPYYIR---PSQAGLLAHFAAVADAA--ALPIILYNIPYRT
N91_1294     ------PYPGQPSFRGIWIPLVTPFR--------DGEVDYPALRKLVKHYLEA-GVDGLVVCGTTGEAAALTEAEQLAVLDAVLEAVG--ALPVVMGL----SGNNLREVLELLARLAERPIAGVLVPAPYYIR---PSQAGLLAYFAAVADAA--AVPIILYDIPYRT
N92_1291     -----MQYPGQPSFRGIWIPLVTPFR--------DGAVDYPALRKLVKHYRAA-GVDGLVACGSTGEAAALTEAEQLAVLDAVLEAAG--ALPVVMGL----SGNNLGHVLERLARLAERPLAGVLVPAPYYVR---PSQAGLLAYFAALADAS--AVPLILYDIPYRT
N93_1312     -----ELGQM--MFRGVITALVTPFKE-------DGEFDYEALRKLVEFQIEN-GVDGIVVCGTTGESPTLTHEEHKELIKTVVEAVGG-RVPVIAGT----GSNNTREAIELTKHAAEVGVDGVLVVTPYYIK---PSQEGLYAHFKAVAEAG--DLPIILYNVPGRT
N94_1365     LEDKNRTNTDDIKSLRVITAIKTPYLP-------DGRFDLEAYDDLVNIQIEN-GAEGVIVGGTTGEGQLMSWDEHIMLIGHTVNCFGG-RIKVIGNT----GSNSTREAIHATEQGFAVGMHGALHINPYYGK---TSIEGLNAHFQTVLHMG----PTIIYNVPGRT
N95_1365     LEVKNRTNTDDIKSLRVITAIKTPYLP-------DGRFDLEAYDDLVNIQIEN-GAEGVIVGGTTGEGQLMSWDEHIMLIGHTVNCFGG-SIKVIGNT----GSNSTREAIHATEQGFAVGMHAALHINPYYGK---TSIEGLIAHFQSVLHMG----PTIIYNVPGRT
N96_1365     LEVKNRTSTDDIKSLRLITAIKTPYLP-------DGRFDLEAYDALVNMQIED-GAEGVIVGGTTGEGQLMSWDEHIMLIGHTVNCFGG-SIKVIGNT----GSNSTREAIHATEQGFAVGMHAALHINPYYGK---TSIEGLISHFESVLPMG----PTIIYNVPSRT
N97_1373     -----RTSTDDIKSLRLITAVKTPYLP-------DGRFDLEAYDSLINMQIEG-GAEGVIVGGTTGEGHLMSWDEHIMLIGHTVNCFGA-NIKVIGNT----GSNSTREAVHATEQGFAVGMHAALHINPYYGK---TSVEGLISHFEAVLPMG----PTIIYNVPSRT
N98_1373     -----RTSTDDITSLRLITAVKTPYLP-------DGRFDLEAYDSLINMQIEG-GAEGVIVGGTTGEGHLMSWDEHIMLIGHTVNCFGA-KIKVIGNT----GSNSTREAVHATEQGFAVGMHAALHINPYYGK---TSVEGLISHFEAVLPMG----PTIIYNVPSRT
N99_1304     ----GGGELGQMMFRGVYTALVTPFKE-------DGEVDYEALRKLVEFQIEN-GVDGIVVCGTTGESPTLTHEEHKELIKTVVEAVNG-RVPVIAGT----GSNNTREAIELTKHAAEVGVDGVLVVTPYYNK---PSQEGLYQHFKAVAEAA--DLPIILYNIPGRT
N100_1292    ------------MFKGVITALVTPFKE-------DGEVDYEALRKLVEFQIEN-GVDGIVVCGTTGESPTLTHEEHKEVIKIVVEAVNG-RVPVIAGT----GSNNTREAIELTKHAEEVGADAVLVVTPYYNK---PSQEGLYQHFKAIAEAV--DIPIILYNVPGRT
N101_1292    ------------MFEGVIPALVTPFKE-------DGEVDYEGLRKNVEFLIEN-GVNGIVPCGTTGESPTLSHEEHKEVIKIVVEAVNG-RVPVIAGT----GSNNTREAIELTKHAEEVGADAVLLVTPYYNK---PSQEGLYQHFKKIAEAV--DIPIILYNVPSRT
N102_1290    ------------MFEGVIPALVTPFKE-------DGEVDYEGLRKNVEFLEEN-GVNGIVPCGTTGESPTLSHEEHKEVIKIVVEAV---RVPVIAGT----GSNNTREAIELTKHAEEVGADAVLLVTPYYNK---PSQEGLYQHFKKIAEAV--DIPIILYNVPSRT
N103_1292    ------------MFEGVIPALITPFKE-------DKEVDYEGLRENVEFLIEN-GVNGIVPCGTTGESPTLSHEEHKKVIEIVVEAVNG-RVPVIAGA----GSNSTREAIELSTYAEEVGADAVLSVTPYYNK---PSQEGLYQHFKKIAEAV--DIPIILYNVPSRT
N104_1291    ------------MFKGSITALVTPFKE--------GEVDYEALRKLVEFQIEN-GTDAIVVCGTTGESPTLTHEEHKEVIKIVVEAVNG-RVPVIAGT----GSNNTREAIELTKHAEEVGADAVLVVTPYYNK---PSQEGLYQHFKAIAEAV--DIPIILYNVPGRT
N105_1291    ------------MFKGSITALVTPFKE--------GEVDYEALRKLVEFQIEN-GTDAIVVCGTTGESPTLTHEEKKEVIKIVVEAVNG-RVPVIAGT----GSNNTREAIELSKHAEEVGADAVLVVTPYYNK---PSQEGLYQHFKAIAEAV--DIPIILYNVPGRT
N106_1291    ------------MFKGSGVALVTPFKE--------GEVDYEALRKLVEFHIEN-GTDAIVVCGTTGESPTLTHEEKKEVIKIVVEAVNG-RVPVIAGT----GSNNTREAIELSKHAEEVGADAVLVVTPYYNK---PSQEGLYQHFKAIAEAV--DIPIILYNVPGRT
N107_1291    ------------MFKGSGVALVTPFTE--------GEVDYEALRKLVEFHIEN-NTDAIVVCGTTGESPTLTHEEKKEVIKIVVEVVNK-RVPVIAGT----GTNNTKESIELSKHAEEVGADAVLVITPYYNK---TSQKGLYQHFKAIADAV--DIPIILYNVPSRT
N108_1294    ----------THMFEGVGVALATPFT--------NNEVDFEALKRHVNFLLEN-NAQAIIVNGTTAESPTLTTEEKEKVLKTVVDLVNK-RVPVIAGT----GTNNTEKSIQASIQAKEIGADAVMLITPYYNK---TNQRGLVQHFEAIADAV--KLPVILYNVPSRT
N109_1297    ------------MFKGSGVALVTPFKE-------DGEVDYEALRKLVEFHIEN-GTDAIVVCGTTGESPTLTHEEKKEVIKIVVEAVNG-RVPVIAGT----GSNNTREAIELSKHAEEVGADAVLSVTPYYNK---PSQEGLYQHFKAIAEAV--DIPIILYNVPGRT
N110_1291    ------------MFKGSITALVTPFKE--------GEVDYEALRKLVEFQIEN-GTDAIVVCGTTGESPTLTHEEHKKVIKIVVETVNG-RVPVIAGT----GSNNTAEAIELTKHAKEVGADAVLVVTPYYNK---PTQEGLYQHFKAIAEAV--DIPIILYNVPGRT
N111_1291    ------------MFKGSITALVTPFTE--------GAVDYEKLRELVEFQIEN-GTDAIVPCGTTGESPTLTHEEHKKVVKIVVETVNG-RVPVIAGT----GSNNTAEAIELTRHAKEAGADAVLVVTPYYNK---PTQEGLYQHFKAIAEAV--DIPIILYNVPGRT
N112_1291    ------------MFKGSITALVTPFKE--------GEVDYEALRKLVEFQIEN-GTDAIVVCGTTGESPTLTHEEHKKVIKIVVETVNG-RVPVIAGT----GSNNTAEAIELTKHAKEVGADAVLVVTPYYNK---PTQEGLYQHFKAIAEAV--DIPIILYNVPGRT
N113_1292    ------------MFKGSITALITPFKE--------GEVDYEALGKLIEFQIEN-GTDAIVVCGTTGESPTLTHEEHKKVIEIAVETVNG-RVPVIAGT----GSNNTHEAVELTKHAKEVGADAVLVVVPYYNK---PTQEGLYEHFKTIAEAV--DIPIILYNVPGRT
N114_1293    ------------MIIGSMTALITPFKK--------GKVDEEAYGRLIKRQIEN-GIDAVVPVGTTGESATLTHEEHRKCIEIAVETCKGSRVKVLAGA----GSNATHEAVGLAKFAKEIGADAILSVAPYYNK---PTQQGLYEHYKTIAQSV--DIPVMLYNVPGRT
N115_1293    ------------MFKGSITALITPFKE--------GEVDYEALGKLIEFQIEN-GTDAIVVCGTTGESPTLTHEEHKKVIEIAVKTVNG-RVPVIAGT----GSNNTHEAVELTKHAKEVGADAVLVVVPYYNK---PTQEGLYEHFKTIAEAV--DIPIIIYNIPGRT
N116_1291    ------------MFKGSITALVTPFKE--------GEVDYEALRKLVEFQIEN-GTDAIVVVGTTGESPTLTHEEHKKVIKLVVETVNG-RVPVIAGT----GSNNTAEAIELTQHAKEVGADAVLVVTPYYNK---PTQEGLYQHFKAIAEAV--DIPIILYNVPGRT
N117_1291    ------------MFKGSITALVTPFKE--------GEVDYKALRKLVEWQIEN-GTDAIVVVGTTGESPTLSHEEHKRVIKLVVETVNG-RVPVIAGA----GSNNTAEAIELAQHAKEVGADAVLVVTPYYNK---PTQEGLYQHFKAIAEAV--DIPIILYNVPGRT
N118_1291    ------------MFKGSITALVTPFKE--------GEVDYKALRKLVEWQIEN-GTDAIVVVGTTGEAPTLSHEEHKLVIKLAVEIVNG-RVPVIAGA----GSNNTAEAIELAQHAKEVGADAVLAVTPYYNK---PTQEGIYQHFKAIAEAV--DLPIILYNVPGRT
N119_1298    ------------WFTGFIPDLPTPFNE-------AGDIDLKALTRLVERQIAS-GASAIVVCGTAGEAPTLSPAEQELVIRAAVDIAGG-RVRVIAGA----GSNATSQAIELARRAQAAGADAVLAVVPYYNK---PTQEGILAHFRAIAGAI--ELPVILHDVPSRT
N120_1291    ------------MFKGSITALVTPFKD--------GEVDEKALRALVEWQIEE-GTHGLVPVGTTGESPTLSHEEHKRVVELCVETANG-RVPVIAGA----GSNNTAEAIELAQHAKEVGADAVLVVTPYYNK---PTQEGLYQHFKAIAEAV--DIPIIVYNVPGRT
N121_1292    -------------MFKGSITALVTPFTD--------GRVDEKALRALVEWQIEE-GTHGLVPVGTTGESPTLSHEEHKRVVELCVETANG-RVPVIAGA----GSNNTAEAIELAQHAQEAGADAVLVVTPYYNK---PTQEGLYQHFKAIAEAV--DIPIIVYNIPGRS
N122_1292    -------------MFKGSITALVTPFTD--------GRVDEKALRALVEWQIEE-GTHGLVPVGTTGESPTLSHEEHKRVVELCVETANG-RVPVIAGA----GSNNTAEAIELAQHAQEAGADAVLVVTPYYNK---PTQEGLYQHFKAVAEAV--DIPIIVYNIPPRS
N123_1293    -------------MFKGSITALVTPFTD-------NGRVDEKAFRALVEWQIGE-GTNGLVPVGTTGESPTLSHDEHKRVVELCIEVANG-RVPVIAGA----GSNNTAEAIELAQHAQEAGADAVLVVTPYYNK---PTQKGLYEHFSAVAEAV--KLPIIIYNIPPRS
N124_1292    -------------MFTGSIVALVTPMNE-------DGEVDYESLKKLVDFHIEN-GTDAIVAVGTTGESATLTVEEHIKVIKQVVEQVNG-RIPVIAGT----GANSTAEAIELTQRAKEVGADACLIVTPYYNK---PTQEGLYQHFKAIAEAV--DIPQILYNVPGRT
N125_1292    -------------MFTGSLVALVTPMNE-------DGEVDYESLKKLVDFHIEN-GTDAIVAVGTTGESATLTVEEHIKVIKQVVEQVNG-RIPVIAGT----GANSTAEAIELTQRAKEVGADACLIVTPYYNK---PTQEGLYQHFKAIAEAV--DIPQILYNVPGRT
N126_1292    -------------MLTGSLVALVTPMNE-------DGRVDYESLKKLVDFHIEN-GTDAIVAVGTTGESATLDVEEHIKVIKQVVEQVKG-RIPVIAGT----GANSTAEAIELTQRAKEVGADACLLVTPYYNK---PTQEGLYQHFKAIAEAV--DIPQILYNVPGRT
N127_1292    -------------MLTGSLVALVTPMNE-------DGRVDYEALKKLVDFHIEN-GTDGIVAVGTTGESATLDVEEHIAVIKAVVEHVKG-RVPVIAGT----GANSTAEAIELTQRAKEVGADACLLVTPYYNK---PTQEGLYQHFKAIAEAV--DIPQILYNVPGRT
N128_1291    -------------MLTGSLVALVTPMNE-------DGRVDYEALKKLVDFHIEN-GTDGIVAVGTTGESATLSVEEHIAVIKAVVEHVKG-RVPVIAGT----GANSTAEAIELSQRAKEVGADACLSVVPYYNK---PTQEGIYQHFKAIAEAT--SIPMILYNVPGRT
N129_1292    -------------MIAGSMVALVTPFDA-------QGRLDWDSLAKLVDFHLQE-GTNAIVAVGTTGESATLDVEEHIQVIRRVVDQVKG-RIPVIAGT----GANSTREAVALTEAAKSGGADACLLVTPYYNK---PTQEGMYQHFRHIAEAV--AIPQILYNVPGRT
N130_1292    -------------MFTGSIVALVTPMNE-------DGEVDYASLKKLVDFHIDN-GTDAIVAVGTTGESATLTVEEHVKVVKQTVELADG-RIPVIAGT----GANATAEAIELTQRLNEVGIAGCLTVTPYYNK---PTQEGLYQHFKAIAEAT--DIPQILYNVPGRT
N131_1292    -------------MFTGSIVALITPMNP-------DGEVDYASLKKLVDFHIDN-GTDAIVAVGTTGESATLTVEEHVKVVAQTVEFADG-RIPVIAGT----GANATAEAIELTQRLNEVGIAGCLSVTPYYNK---PTQEGLFQHYKAIAEAT--DIPQILYNVPGRT
N132_1292    -------------MFTGSIVALVTPMNE-------DGEVDYASLKKLIDYHIAN-GTSAIVAVGTTGESATLTVEEHVKVVKQTLELADG-RIPVIAGT----GANATAEAIELTQRFNEVGIAGCLTVTPYYNR---PTQEGLYQHFKAIAEHT--DIPQILYNVPGRT
N133_1226    ----------LLKLLKLID--LTALK---------DDATEEDIDKLCQQAIEG-GFYAICIY-------------PRFIPLAREALKTPEVRVATVTGFPLGHNDTEVAVYEAKVAIEYGADEVDVVFPYRALM-AGNEEVVFELIKAVKEAC--AAAVLKVIIETAL
N134_1226    -------------ELNKYID--HTALK---------PDATEEDIDKLCQEAIEY-GFYAVCVN-------------PSYVPLAREALKNTDVKVCTVIGFPLGHNATEVKVYEAKVAIENGADEIDMVINIGAFK-AGKYDYVLEEIRAVKEAC--AGAVLKVIIETAL
N135_1226    -------------ELNKYID--HTALK---------PDATEEDIDKLCQEAIEY-GFYAVCVN-------------PSYVPLAREALKNTDVKVCTVIGFPLGHNATEVKVYEAKVAIENGADEIDMVINIGAFK-AGKYDYVLEEIRAVKEAC--AGAVLKVIIETAL
N136_1221    -------------ELAKYID--HTALK---------PDATEEDIAKLCQEAIEY-GFYAVCVN-------------PSYVPLAREALKNTDVKVCTVIGFPLGHNATEVKAYEAKVAIENGADEIDMVINIGALK-AGKYDYVQEEIRAVKEAC--AGAVLKVIIETAL
N137_1218    ------------MELAAHID--HTLLK---------PDATEEDVAKLVQEAIEY-GFYAVCVS-------------PSYVPLAREALKNTGVKVCTVIGFPLGHNATEVKAYEAAVAIENGADEIDMVINIGAAK-AGDYDYVQAEIRAVKEAA--AGAVLKVIIETAL
N138_1218    ------------MELAAHID--HTLLK---------PDATEEDVAKLVQEAIEY-GFYAVCVS-------------PSYVPLVREALKNTGVKLCTVIGFPLGHNATEVKAYEAAVAIENGADEIDMVIHLGAAK-AGDYDYVQAEIRAVKEAA--AGAVLKVILETAL
N139_1221    ------------MELAKYID--HTALK---------PDATEEDIAKLCQEAIEY-GFYSVCVN-------------PSYVPLAREELKGTDVKVCTVIGFPLGANATEVKAYEAKVAIENGADEIDMVINIGALK-AKKWDYVQEDIRAVVEAC--AGAVLKVIIETCL
N140_1220    ------------MELANYID--HTALK---------PDATEEDIAKLCQEAREY-RFYSVCVN-------------PSYVPLAREELKGTDVKVCTVIGFPLGANATETKAYEAKVAIENGADEIDMVINIGALK-AKEWDYVQEDIRAVVEAV--AGAVVKVIIETCL
N141_1223    -------------ELNKYID--HTALK---------PDATEEDIDKLCQEAIEY-GFYAVCVN-------------PSYVPLAREILKNTDVKVCCVIGFPLGHNATEVKVYEAKVAIENGADEIDMVINIGAFK-AGKYDYVLEEIRAVKKAC--AGAVLKVIIETAL
```

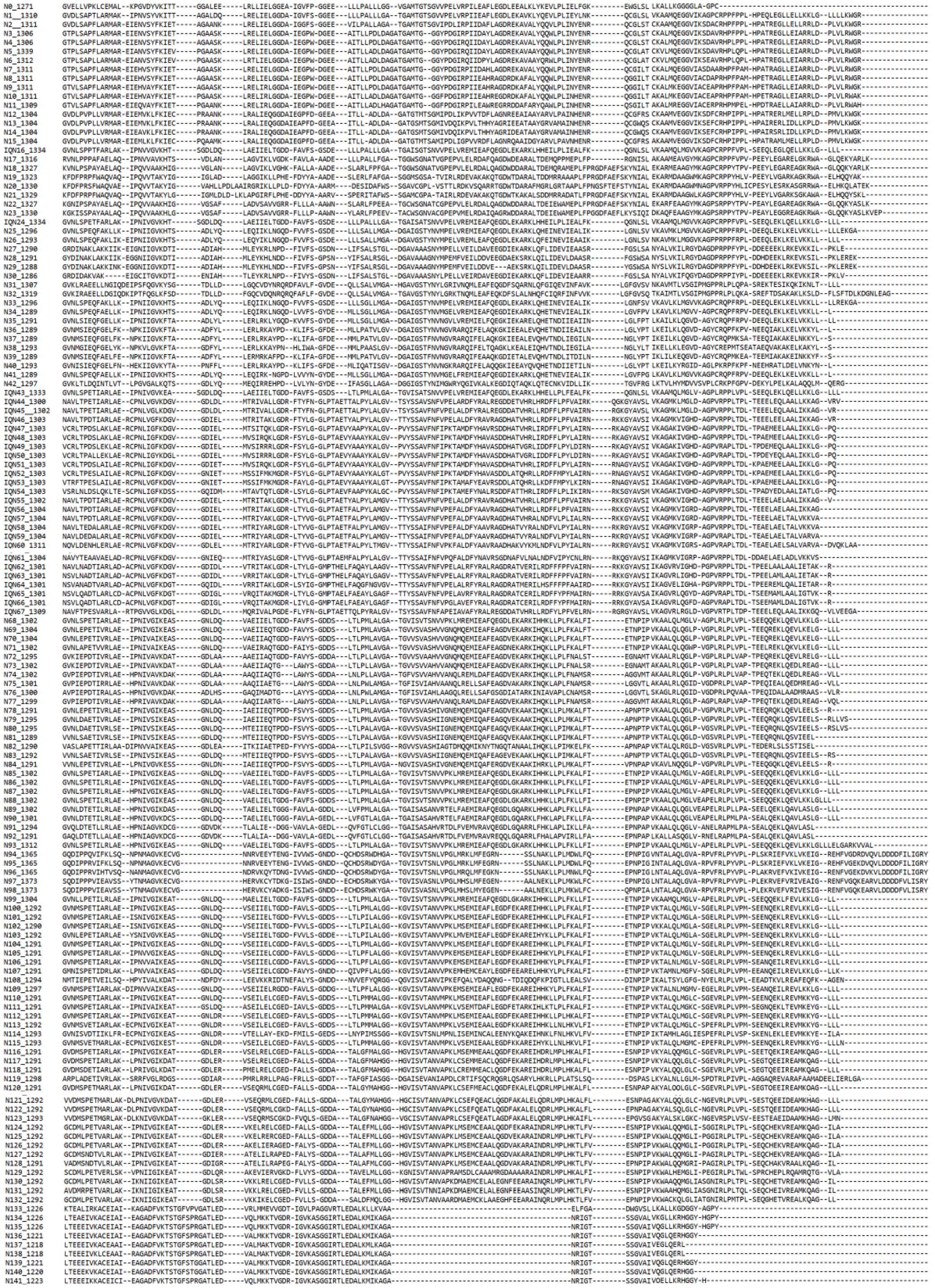

Figure S34: Multiple sequence alignment with the reconstructed sequences from the phylogenetic tree using the GRASP tool. The alignment was performed with promals3D. The alignment was cut at the N-terminus due to long regions with low homology.

Melting point determination via Thermofluor assay:

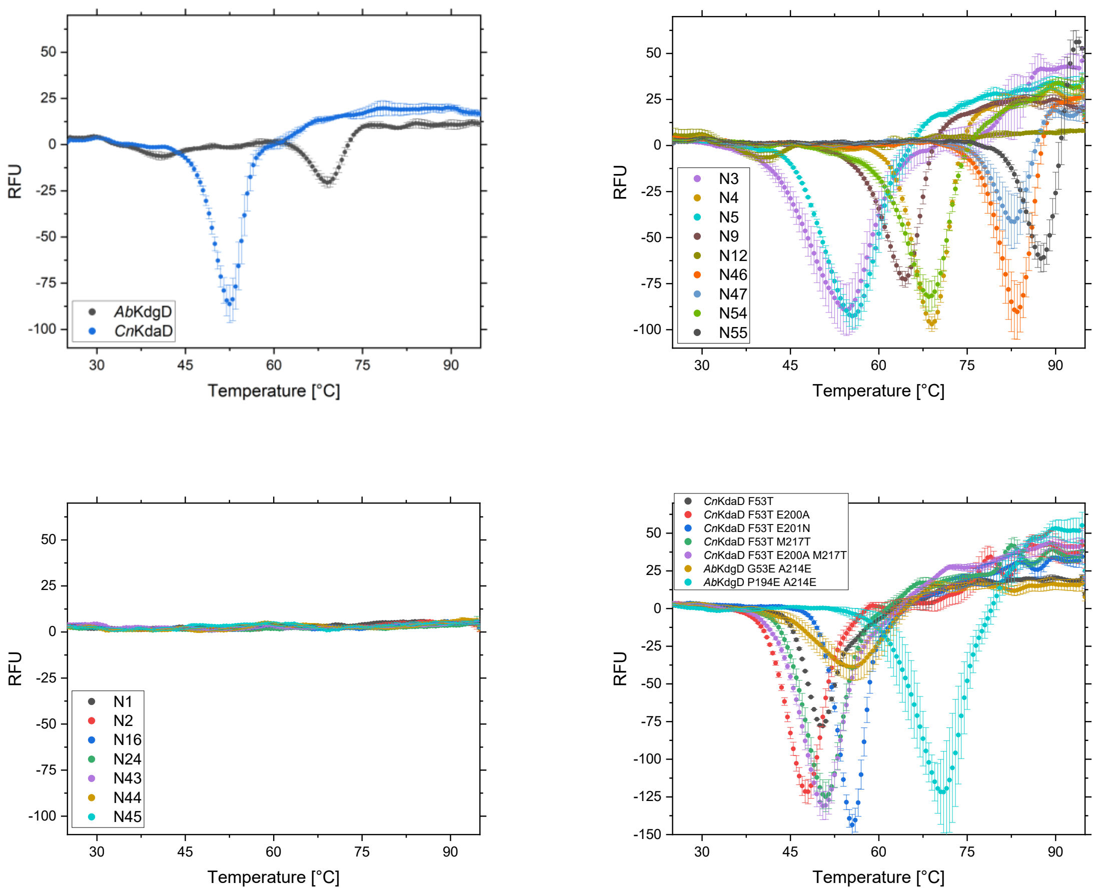

Figure S35: Melting curves of the purified enzymes of this study. The curves were measured using SPYRO orange as a dye for the Thermoflur assay. Shown are the curves for the KdaD and KdgD wildtypes (top left), the soluble Ancestors (top right), the Ancestors that were expressed with an MBP-tag, after cleavage of the MBP-tag (bottom left) and the generated variants of *Cn*KdaD and *Ab*KdgD (bottom right).

Table S8: Melting points of the enzymes determined via Thermoflur assay. Measurement was performed in triplicates with the listed error being calculated from the standard deviation

**Enzyme**	**Melting point [°C]**
*Cn*KdaD	52.3 ± 0.24
*Ab*KdgD	69.0 ± 0
N1	-
N2	-
N3	56.5 ± 0
N4	69.0 ± 0
N5	55.7 ± 0.47
N9	64.3 ± 0.24
N12	40.3 ± 0.47
N16	-
N24	-
N43	-
N44	-
N45	-
N46	83.3 ± 0.24

N47	82.8 ± 0.24
N54	68.5 ± 0
N55	87.7 ± 0.24
*Cn*KdaD F53T	50.5 ± 0
*Cn*KdaD F53T E200A	47.8 ± 0.24
*Cn*KdaD F53T E201N	55.2 ± 0.24
*Cn*KdaD F53T M217T	51 ± 0
*Cn*KdaD F53T E200A M217T	50.7 ± 0.24
*Ab*KdgD G53E A214E	55.7 ± 0.94
*Ab*KdgD P194E A214E	70.5 ± 1.41

HPLC Analysis:

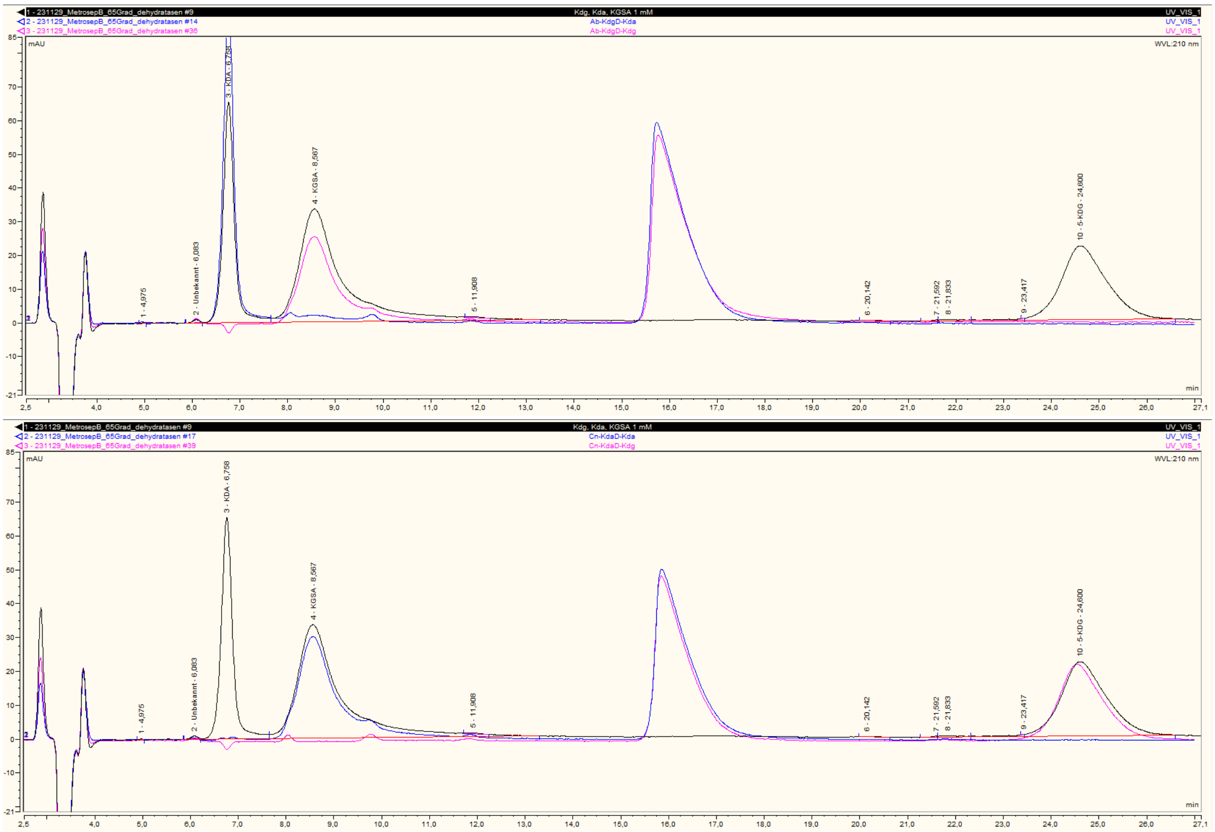

Figure S36: HPLC analysis of the reaction of *Ab*KdgD and *Cn*KdaD with 5-Keto-4-deoxy glucarate (Kdg) and 2-Keto-3-deoxy-L-arabinonate (Kda). The reaction was analyzed on the Metrosep Anion exchange column with an isocratic flow of 30 mM ammonium bicarbonate pH 10.4 at 65 °C column temperature. The analysis shows that the enzymes only form the product, KGSA, from their native substrate. The peak at t = 16 min corresponds to the used buffer, HEPES.

pK_a Predictions of *Ab*KdgD and the generated variants

Table S9: pK_a predictions of *Ab*KdgD and the characterized variants generated using PropKa in the APBS biomolecular solvation software suite with the amber force field [22].

Residue	Position	*Ab*KdgD	*Ab*KdgD P194E	*Ab*KdgD G53E A214E	*Ab*KdgD P194E A214E
ASP	2	3.19	3.20	3.4	3.76
ASP	12	3.82	3.05	2.7	3.71
ASP	21	4.1	4.00	4.14	4.02
ASP	23	3.3	2.93	3.02	3.26
ASP	27	2.94	3.27	3.32	2.9
ASP	63	3.99	3.98	3.99	3.99
ASP	67	4.47	4.47	4.52	4.46
ASP	74	2.68	2.94	3.21	2.91
ASP	151	3.86	3.86	3.88	3.82
ASP	168	4.59	4.75	4.77	4.49
ASP	174	4.23	4.18	4.19	4.23
ASP	185	3.84	3.67	3.7	3.65
ASP	233	3.03	3.28	3.25	3.39
ASP	234	2.74	2.85	2.76	2.75
ASP	244	3.63	3.92	3.91	3.59
ASP	274	3.44	3.37	3.46	3.41
ASP	284	5.48	5.67	5.63	4.99
ASP	289	2.11	2.24	2.26	2.22
ASP	292	2.69	2.67	2.78	2.75
GLU	5	4.84	4.71	4.66	4.65
GLU	38	4.69	4.71	4.72	4.63
GLU	53			4.77	
GLU	56	5.13	5.17	5.16	5.43
GLU	64	3.81	3.56	3.68	3.56
GLU	99	2.45	2.52	2.46	2.48
GLU	101	4.38	4.62	4.38	4.35
GLU	117	3.94	5.45	3.54	4.82
GLU	121	3.19	3.19	3.23	3.2
GLU	125	4.24	3.99	4.21	4.21
GLU	158	4.17	4.61	4.69	4.72
GLU	178	5.54	4.58	5.48	4.65
GLU	194		4.80		5.41
GLU	197	6.82	6.80	6.93	6.87
GLU	214			7.6	7.4
GLU	225	4.72	4.45	4.36	4.79
GLU	291	4.78	4.81	4.79	4.79
HIS	106	5.15	5.28	5.23	5.19
HIS	113	4.16	4.06	4.18	4.15
HIS	126	2.76	2.87	2.78	2.83
HIS	273	5.1	5.15	5.03	5.22
CYS	76	11.1	11.31	11.31	11.3
CYS	131	16.81	16.79	16.78	16.73

CYS	160	6.31	6.24	7.01	6.33
TYR	33	13.76	13.80	13.86	13.83
TYR	43	11.49	11.31	11.4	11.41
TYR	65	13.8	13.65	13.75	13.7
TYR	114	13.43	13.31	13.88	13.43
TYR	141	11.63	8.13	14.13	9.06
TYR	189	13.04	13.03	12.93	12.96
TYR	203	15.79	15.32	15.44	15.85
TYR	211	15.43	15.57	15.55	15.41
TYR	227	11.46	11.26	11.71	11.66
TYR	259	12.11	12.26	12.23	12.12
TYR	290	10.68	10.62	10.76	10.66
LYS	7	9.53	9.79	9.76	9.51
LYS	35	10.47	10.48	10.38	10.48
LYS	70	11.38	11.43	11.21	11.52
LYS	77	10.71	10.43	10.46	10.51
LYS	128	10.68	10.67	10.68	10.7
LYS	147	10.07	10.08	9.98	10.04
LYS	167	6.16	9.18	6.94	9.09
LYS	204	10.66	10.54	10.57	10.83
LYS	221	10.56	10.57	10.58	10.59
LYS	251	10.6	11.37	10.54	11.31
LYS	256	11.21	11.47	11.4	11.11
LYS	265	9.34	9.31	9.32	9.35
LYS	269	10.96	10.95	10.98	10.98
ARG	36	12.86	12.85	12.77	12.79
ARG	91	12.22	12.28	12.18	12.98
ARG	102	14.14	14.10	14.11	14.11
ARG	143	13.6	13.49	16.72	13.21
ARG	186	11.51	11.57	11.56	11.54
ARG	231	12.55	13.80	13.89	12.64
ARG	240	12.81	13.00	13.11	12.6
ARG	243	12.64	12.12	12.17	12.71
ARG	253	10.81	11.64	11.34	12.22
ARG	255	14.35	14.33	14.34	14.33
ARG	279	12.95	12.97	12.97	12.27

pK_a Predictions of *Cn*KdaD and the generated variants

Table S10: pK_a predictions of *Cn*KdaD and the characterized variants generated using PropKa in the APBS biomolecular solvation software suite with the amber force field [22].

**Residue**	**Position**	***Cn*KdaD**	***Cn*KdaD F53T**	***Cn*KdaD F53T E200A**	***Cn*KdaD F53T E201N**	***Cn*KdaD F53T M217T**	***Cn*KdaD F53T E200A M217T**
ASP	22	2.86	2.79	2.85	2.87	2.86	2.83
ASP	28	3.8	3.66	3.85	3.88	3.87	3.86
ASP	30	3.46	2.7	3.55	3.43	3.49	2.83

ASP	37	2.77	2.76	2.82	2.8	2.73	2.74
ASP	41	3.65	3.66	3.72	3.65	3.67	3.69
ASP	61	1.39	1.54	2.49	3.07	1.21	3.73
ASP	62	4.46	4.51	4.31	4.3	4.29	4.49
ASP	76	3.83	3.84	3.83	3.83	3.83	3.83
ASP	102	4.01	4.05	4.05	4.05	3.99	4.05
ASP	135	4.08	4.09	4.09	4.09	4.09	4.11
ASP	138	3.3	3.59	3.55	3.55	3.44	3.6
ASP	145	5.51	5.46	5.48	5.48	5.52	5.44
ASP	191	3.9	3.9	3.9	3.9	3.9	3.9
ASP	198	10.24	10.16	9.93	10.1	10.24	10.31
ASP	208	6.35	6.4	6.4	6.34	6.36	6.24
ASP	224	5.2	5.21	5.18	5.21	5.24	5.33
ASP	231	3.2	3.23	3.25	3.18	3.16	3.17
ASP	237	3.31	3.34	3.38	3.33	3.37	3.25
ASP	277	3.46	3.51	3.58	4.25	3.64	3.67
ASP	302	3.65	3.79	3.8	3.75	3.74	3.72
GLU	55	5.2	5.22	5.27	5.23	5.16	5.15
GLU	63	3.07	3.19	3.23	3.16	3.21	3.38
GLU	73	4.54	4.59	4.47	4.54	4.56	4.57
GLU	95	4.65	4.59	4.68	4.82	4.72	4.53
GLU	123	4.92	5.46	5.37	5.43	5.47	5.4
GLU	128	2.23	2.22	2.17	2.49	2.23	2.27
GLU	164	4.02	3.95	3.5	3.54	3.41	3.41
GLU	174	4.1	3.89	4.08	4.67	3.86	3.88
GLU	184	5.26	5.28	5.27	5.25	5.29	5.3
GLU	187	4.57	4.58	4.6	4.59	4.59	4.59
GLU	200	5.2	4.39		4.12	4.54	
GLU	201	7.55	7.13	5.91		7.06	6.15
GLU	210	4.69	4.22	4.21	4.38	4.24	4.45
GLU	239	2.91	2.95	3.2	2.99	3.14	3.83
GLU	255	8.99	9.03	8.98	7.99	8.93	9.04
GLU	292	4.54	4.62	4.59	4.58	4.61	4.6
HIS	45	4.88	4.93	4.94	4.9	4.92	4.9
HIS	74	6.5	6.44	6.39	6.45	6.41	6.41
HIS	87	3.07	2.29	2.85	1.71	2.65	2.08
HIS	115	3.57	3.44	3.27	3.41	3.45	3.66
HIS	281	6.03	5.98	6.05	5.96	6.01	6.04
HIS	287	5.69	5.64	5.88	5.81	5.72	5.75
CYS	35	12.44	12.42	12.42	12.41	12.44	12.44
CYS	48	11.41	11.4	11.41	11.4	11.21	11.2
CYS	93	11.44	11.44	11.43	11.42	11.45	11.32
TYR	10	10.93	10.94	10.93	10.96	10.94	10.97
TYR	114	13.15	13.28	12.83	12.78	13.35	12.86
TYR	127	11.42	11.27	11.27	11.3	11.25	11.27
TYR	170	11.63	11.54	11.57	11.57	11.59	11.56
TYR	245	13.93	13.91	13.91	13.9	13.97	13.96

TYR	254	12.56	12.34	12.3	12.21	12.38	12.33
LYS	69	10.45	13.92	10.67	10.43	10.37	10.41
LYS	172	11.34	12.32	11.51	11.12	11.55	11.53
LYS	181	10.09	10.41	10.07	10.07	10.09	10.07
LYS	265	9.69	11.49	9.66	9.66	9.68	9.63
LYS	296	11.04	10.07	11.1	10.96	10.99	10.97
LYS	69	10.45	9.66	10.67	10.43	10.37	10.41
LYS	172	11.36	11.09	11.52	11.11	11.57	11.53
LYS	181	10.07	10.4	10.07	10.08	10.1	10.08
LYS	265	9.67	11.5	9.66	9.67	9.69	9.63
LYS	296	11.01	10.05	11.09	10.99	10.97	11.01
ARG	3	12.47	12.47	12.47	12.48	12.48	12.48
ARG	11	12.26	12.13	12.25	12.34	12.11	12.17
ARG	33	12.71	13.37	12.68	12.79	12.75	13.39
ARG	34	12.31	12.34	12.26	12.27	12.16	12.14
ARG	64	9.09	10.2	9.17	10.05	9.45	9.16
ARG	78	13.15	13.21	13.17	13.18	13.21	13.15
ARG	91	13.25	13.18	12.64	12.3	13.17	13.18
ARG	96	12.35	12.48	12.28	12.35	12.33	12.29
ARG	98	12.49	12.43	12.38	12.39	12.57	12.41
ARG	120	10.73	10.75	10.73	10.63	10.69	10.7
ARG	124	13.47	13.43	13.46	13.41	13.45	13.47
ARG	160	12.98	12.65	13.39	13.28	13.4	13.43
ARG	163	12.21	12.21	12.23	12.21	12.21	12.21
ARG	183	11.75	11.73	11.9	11.7	11.88	11.78
ARG	227	13.17	13.11	13.09	13.19	13.21	13.2
ARG	244	12.17	12.2	12.2	12.19	12.17	12.19
ARG	257	13.82	14.22	11.05	13.75	14.02	11.12
ARG	280	12.65	12.6	13.04	12.85	12.58	12.62
ARG	291	13.09	13.02	13.13	12.47	12.9	12.97
ARG	299	12.25	12.22	12.26	12.28	12.21	12.2
ARG	300	13.28	13.27	12.98	13.29	13.01	13.25
ARG	307	12.9	12.69	12.66	12.72	12.73	12.79
ARG	310	12.45	12.39	12.4	12.54	12.38	12.35
